# An Accelerated Method for Investigating Spectral Properties of Dynamically Evolving Nanostructures

**DOI:** 10.1021/acs.jpclett.3c00395

**Published:** 2023-04-20

**Authors:** Yibin Jiang, Abhishek Sharma, Leroy Cronin

**Affiliations:** School of Chemistry, University of Glasgow, University Avenue, Glasgow G12 8QQ U.K.

## Abstract

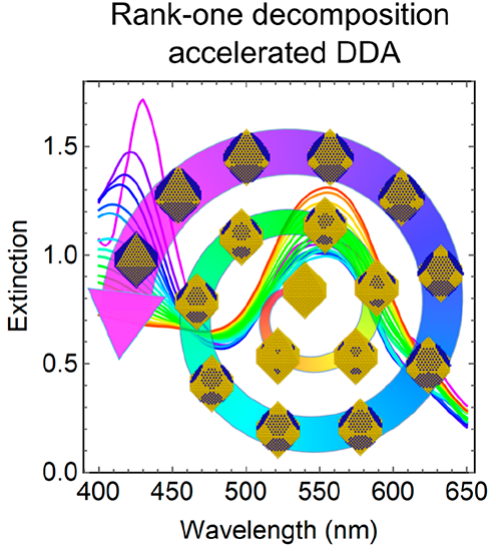

The discrete-dipole approximation (DDA) is widely applied to study the spectral properties of plasmonic nanostructures. However, the high computational cost limits the application of DDA in static geometries, making it impractical for investigating spectral properties during structural transformations. Here we developed an efficient method to simulate spectra of dynamically evolving structures by formulating an iterative calculation process based on the rank-one decomposition of matrices and DDA. By representing structural transformation as the change of dipoles and their properties, the updated polarizations can be computed efficiently. The improvement in computational efficiency was benchmarked, demonstrating up to several hundred times acceleration for a system comprising ca. 4000 dipoles. The rank-one decomposition accelerated DDA method (RD-DDA) can be used directly to investigate the optical properties of nanostructural transformations defined by atomic- or continuum-scale processes, which is essential for understanding the growth mechanisms of nanoparticles and algorithm-driven structural optimization toward enhanced optical properties.

Plasmonic nanomaterials are attractive due to their unique optical properties and applications in surface-enhanced Raman spectroscopy,^1^ nanomedicines,^2^ catalysis,^3^ and optical computation.^4^ This is primarily due to the tunability of the light–matter interactions of metallic nanoparticles, which depend on various factors including geometry, composition, and the surrounding environment.^5^ Various approaches, including bottom-up chemical synthesis^6^ and top-down lithography,^7^ have been used to create nanomaterials with diversified structural features to facilitate unique and application-specific spectral properties.^8−11^ To understand light–matter interactions such as extinction spectra, many computational methods, including finite-difference time-domain (FDTD),^12^ boundary element method (BEM),^13^ and discrete-dipole approximation (DDA),^14−16^ were implemented and have shown good consistency to experimental observations.^17−20^ Among these methods, DDA is widely applied to calculate the extinction spectra and local field distributions of arbitrarily shaped nanomaterials^20−25^ by discretizing the target geometry into lattices of polarizable dipoles to numerically compute the solution for the Maxwell equations.

The growth and etching mechanisms of nanoparticles^23,26−28^ are complex and usually require *in situ* imaging techniques such as transmission electron microscopy (TEM)^23,28^ and atomic force microscopy (AFM).^29^ Spectroscopy techniques like UV–vis–NIR are more cost-efficient compared to *in situ* imaging and are often used to hypothesize mechanisms of morphological transformations. However, the spectroscopic readout related to kinetic processes arises from an ensemble of nanoparticles within a finite volume with variations in geometries, orientations, etc. Additionally, due to the nonuniqueness of the UV–vis–NIR spectrum to a specific geometry and composition, inferring mechanisms describing structural transformations from spectroscopic analysis becomes complicated and empirical. Given an initial structure, an effective and efficient theoretical method is required to investigate structural transformations by comparing simulated spectra from the proposed kinetic model with experimentally observed spectra. Despite the development of DDA, an efficient strategy to track the spectral properties of dynamically evolving nanostructures that captures variations at the atomic scale is still lacking. Temporal structural transformation derived from kinetic models^28,30^ can be at the atomic scale, which generates trajectories comprising a large number of intermediates. Spectral simulations over a complete trajectory by direct implementation of DDA on the emerging intermediates are computationally expensive. This is primarily due to the requirement of solving numerous large linear systems with dense matrices and hence limits its application to only a few selected intermediate structures to validate the proposed model.^23^ A cost-efficient method to simulate the spectral properties of the complete trajectory can offer a more detailed tracking of spectral features due to structural transformations to validate kinetic models with experiments.

Here we implemented a general and computationally efficient method based on DDA to calculate the spectral properties of all the intermediate structures along a trajectory representing the structural transformation. In the original DDA method, a continuum geometry representing a nanostructure is discretized into a finite array of dipoles, whose polarization **P** is solved according to **AP** = **E**, where **E** is the incident electric field and **A** is a matrix whose elements depend on the geometry and the compositions of the dipoles. We hypothesize that if the resolution of dipoles defining the geometry is sufficiently high, then the structural transformation within the nanostructure can be resolved by the addition and removal of dipoles or by updating the properties of existing dipoles.

The proposed method, rank-one decomposition accelerated discrete-dipole approximation (RD-DDA), is based on the rank-one decomposition of matrices. It states that if an invertible matrix **A**′ can be constructed from another invertible matrix **A** and a rank-one matrix **B** so that **A**′ = **A** + **B**, its inverse matrix can be calculated via , where *g* is the trace of **BA**^–1^. Using the DDA formulation, the structural transformation that is represented by the change of the coefficient matrix (from **A** to **A**′) due to addition, removal, and polarizability change of dipoles can be described by the addition of a series of rank-one matrices. Hence, in our approach, **A**^–1^ only needs to be calculated for the initial structure, and the spectral properties such as the extinction of the emerging intermediates can be calculated using an iterative approach employing rank-one decomposition. This approach avoids tediously solving a large linear system comprising dense matrices independently for each intermediate. The comparison between the conventional DDA and iterative RD-DDA approaches is shown in Figure 1a. The rank-one decomposition approach accelerates the computation dramatically by accessing the new solution via only matrix multiplication and addition without matrix inversion. As an example, the computational efficiency was benchmarked by generating a trajectory describing the growth of a 1 nm thin layer of Ag on the Au octahedral surface, resulting in a Au@Ag core–shell octahedron with an edge length of 20 nm. In this case, with the geometry comprising ca. 4000 dipoles, we observed 46- to 250-fold accelerations to simulate the spectra of all the intermediates along the generated trajectory when the direct implementation of DDA and RD-DDA were in the same 64-digit or 128-digit precisions (Figure 1b,c). While a high numeric precision (128-digit) is not necessary for the direct implementation of DDA, 24- and 33-fold accelerations were observed when the direct DDA was implemented under 64-digit precision and RD-DDA under 128-digit precision (Figure S7). The complete information with additional benchmark examples is available in Supporting Information (SI) Section 1.

**Figure 1:**
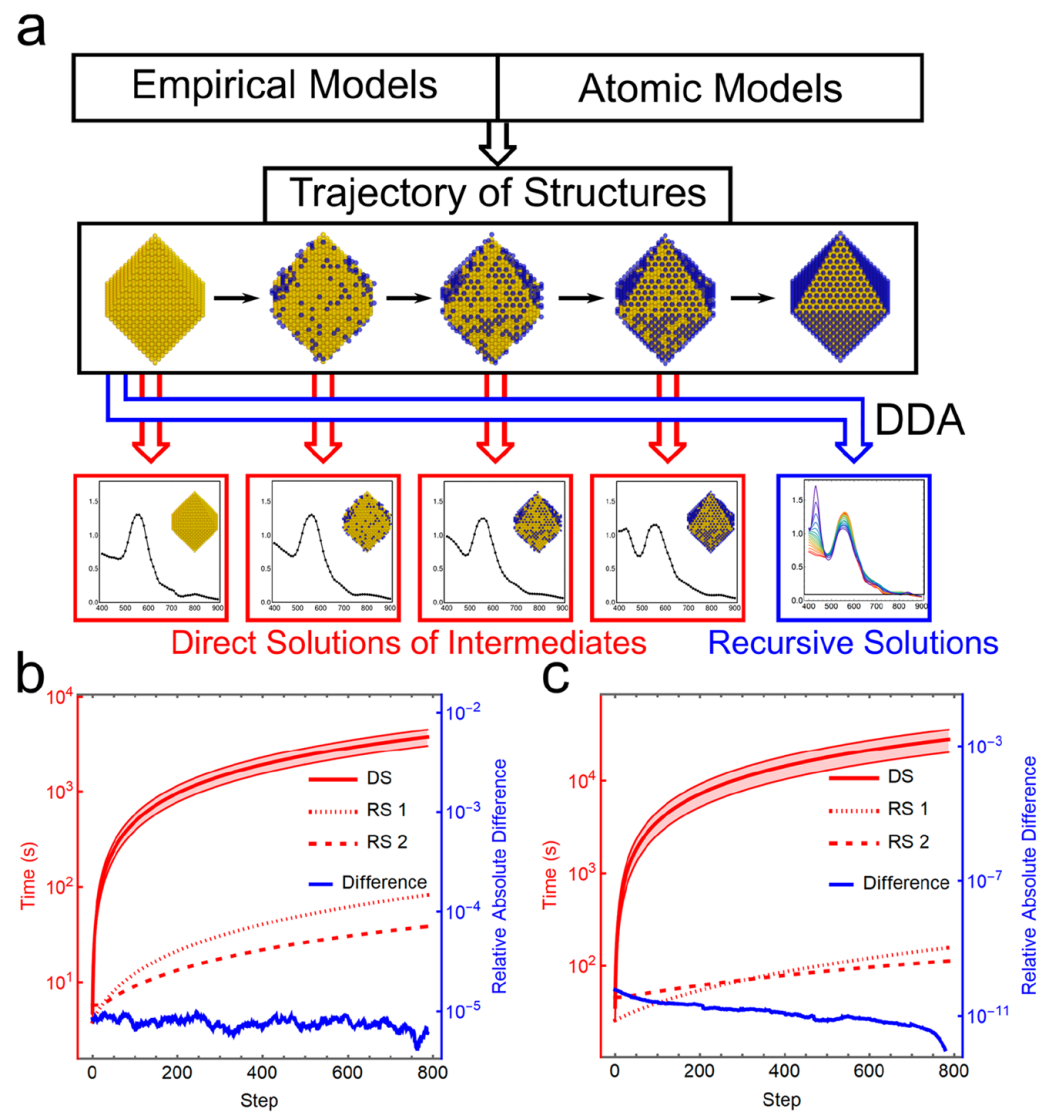
Benchmark of the direct solutions or iterative solutions based on matrix rank-one decomposition for the spectral properties of intermediates in a trajectory. (a) Scheme of the direct solutions of the intermediates and iterative solutions to simulate the spectral properties of nanostructures. The deposition of a single Ag layer on the surface of a Au octahedron with a random growth process is shown here, forming a Au@Ag octahedron with an edge length of 20 nm. (b) Computational time of direct solutions (DS) and iterative solutions with the rank-one decomposition by adding dipoles to the system (RS 1) or changing the polarizability of dipoles (RS 2) when a thin layer of Ag grows on the Au octahedron surface. The relative absolute difference between the extinction cross sections in RS 1 and RS 2 is shown by the blue line. The dipole numbers of initial and final structures are 4089 and 3303, respectively, with 786 etching steps in total. The edge length of the final Au@Ag octahedron is 20 nm. (c) Computational time of various methods for the same system as (b) when a 128-digit complex number is used. It should be noted that 128-digit precision is generally not necessary for direct solutions. For both cases, the dipole length is 1 nm. The time cost of direct solutions is estimated by solving the initial smallest system and the final largest system. All of the computational time is averaged among 51 wavelengths from 400 to 900 nm with an interval of 10 nm for generality.

In the general formulation of DDA, for a given geometry discretized into an array comprising *N* polarizable dipoles, the polarization vector due to the interaction with the external electric field can be approximated by a linear set of equations given by **AP** = **E**,^14^ where **E** is a 3*N* vector describing the local electric field of the incident wave at each dipole position in three-dimensional space, **A** is a 3*N* × 3*N* symmetric matrix with elements depending on the geometry of the structure and its composition, and **P** is a 3*N* vector representing the polarization of the *N* dipoles in the *X*, *Y*, and *Z* directions. **A** is composed of a series of 3 × 3 matrices **A**_*j,k*_ as shown in eq 1:
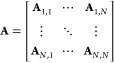
1Within the coefficient matrix **A**, any matrix **A**_*j*,*j*_ at the diagonal position is defined by the inverse of the polarizabilities (α_*j*,*X*_, α_*j*,*Y*_, α_*j*,*Z*_) of the *j*th dipole in the *X*, *Y*, and *Z* directions (eq 2):
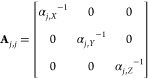
2They are assumed to be the same for simplicity in the later formulation (α_*j*,*X*_ = α_*j*,*Y*_ = α_*j*,*Z*_ = α_*j*_). However, the later-derived formulation is also valid for anisotropic polarization.

For the off-diagonal matrices **A**_*j*,*k*≠*j*_, the elements describe the pairwise dipole–dipole interaction, which is dependent on the relative distance between the *j*th and *k*th dipoles. They are independent of the polarizability of the dipoles and stay constant when the properties of dipoles such as complex refractive index are changed.

To calculate the local electric field, extinction, or scattering spectrum, the polarization (**P**) can be solved via **P** = **A**^–1^**E**. When a dipole (labeled as *j*) is added or deleted from the array of dipoles, the process can be approximated by changing the polarizability of the dipole between the medium (τ, which approximates 0) and the material (α_*j*_) without changing the size of the **A** matrix. During the replacement process, the polarizability would be changed from the original material (α_*j*_) to the new material (α_*j*_^′^) calculated from the refractive index of the material. Thus, due to the change in a single dipole, only the (3*j* – 2)th to (3*j*)th diagonal elements of the symmetric matrix **A** are changed. Since the change in the polarizability of the dipole needs to be updated along the *X*, *Y*, and *Z* directions, the updated coefficient matrix **A**′ can be written as
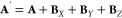
3where **B**_*X*_, **B**_*Y*_, and **B**_*Z*_ are the three sparse rank-one matrices with the (3*j* – 2)th, (3*j* – 1)th, and (3*j*)th diagonal elements equal to α_*j*_^′–1^ – α_*j*_^–1^, respectively.

The inverse matrix of the linear coefficients after adding **B**_*X*_ using rank-one decomposition can be written directly as
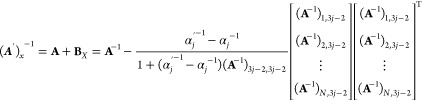
4and its corresponding induced dipoles can be written as
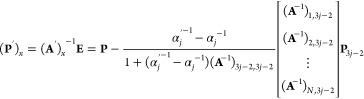
5where **P** is the original polarization (a vector of 3*N* elements) and **P**_3*j*–2_ indicates the (3*j* – 2)th element in vector **P**. By applying this relation (eqs 4 and 5) to sequentially update the inverse matrix and the polarization after adding **B**_*X*_, **B**_*Y*_, and **B**_*Z*_, we can efficiently calculate the new polarizations due to structural transformation defined by the variation at a single dipole. It can also approximate the solution of the system when dipoles are added/removed by assigning very low polarizability (τ ≈ 0) to the dipoles composed of medium, while 128-digit precision is needed when the polarizability of a medium is changed from τ to the polarizability of a material (α_*j*_) for numerical stability. Furthermore, strict analytical solutions for the cases describing the addition, removal, and replacement of a dipole are derived in SI Section 1. The numerical stability and accuracy of the approximation using eqs 4 and 5 and various τ values when the addition or removal of dipoles happens is discussed and validated with the strict formulation, demonstrating low computational cost and negligible errors (see SI Sections 1.5 and 1.6). If not mentioned otherwise, Tait–Bryan angles with 10 sampling points in each angle dimension to represent the uniform distribution of different orientations of the nanostructure were used to calculate an orientation-averaged spectrum through this work.

Using the RD-DDA method, we investigate the time-evolved extinction spectra due to the structural transformation of nanoparticles by the addition or removal of dipoles. Additionally, the optical properties of ensembles composed of these nanostructures can also be simulated thanks to the efficient tracking of the extinction spectra of individual nanostructures. Here we consider the growth of silver on the surface of a gold nanoparticle defined by a discrete process of attaching a single dipole at each step. We used an octahedral gold nanoparticle with an edge length of 19 nm as a substrate and a dipole length of 1 nm.

The extinction spectra of growth of a single layer of Ag were calculated with a final edge length of Ag@Au octahedron of 20 nm in the wavelength range of 400–650 nm. Three distinct modes of Ag growth, including random growth, growth from face centers, and growth from tips, were used to generate the trajectories, as shown in Figure 2a. The spectra corresponding to the intermediate structures in different growth modes were calculated, showing their peculiar spectral features due to the distinct Ag distribution, as shown in Figure 2b–d and Videos S1–S3. The simulation details are available in SI Section 2.1.

**Figure 2:**
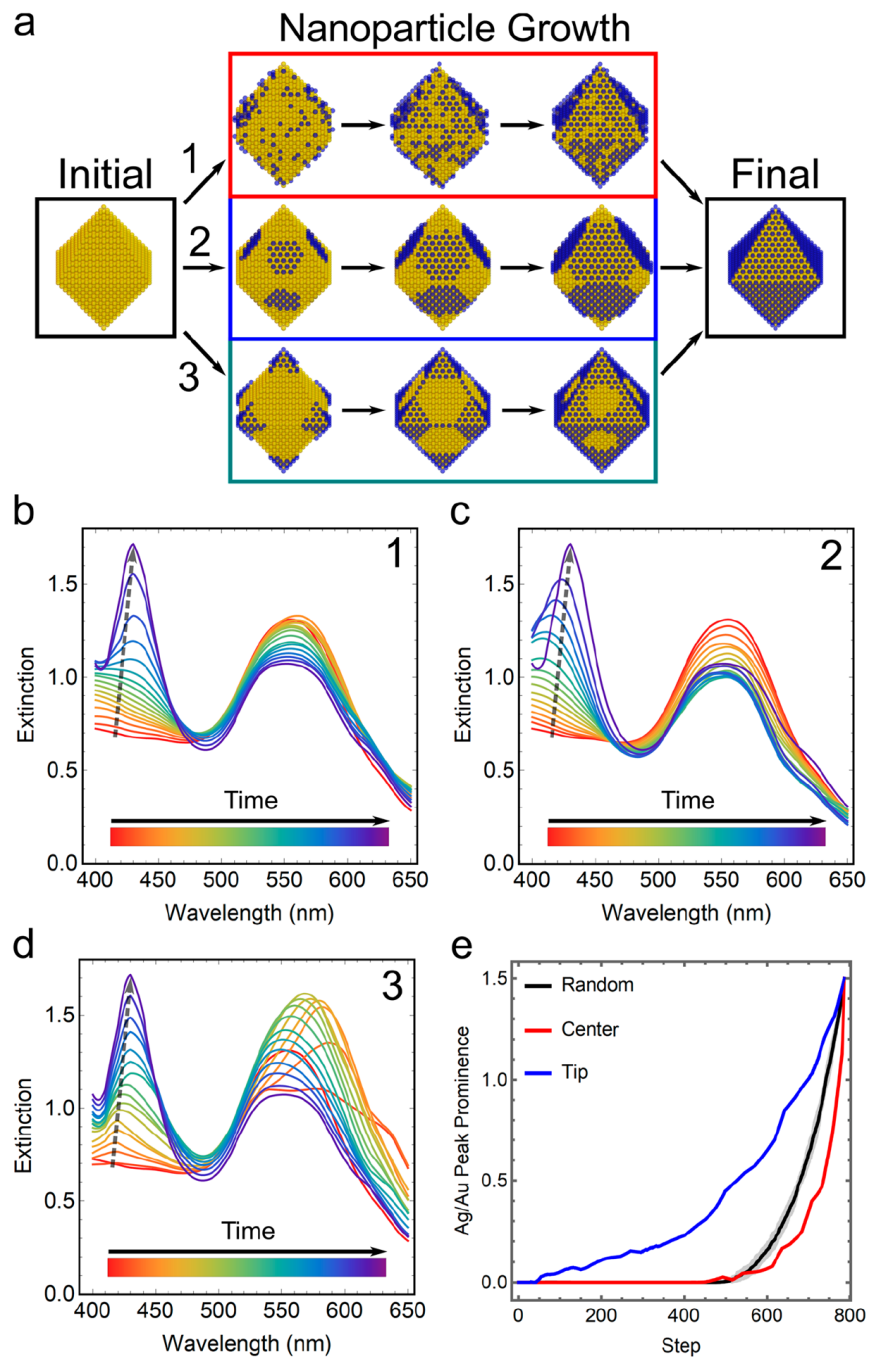
Coating of a thin layer of Ag on the surface of a Au octahedron. (a) Three different growth modes, including (1) random growth, (2) growth from face centers, and (3) growth from tips. (b–d) Extinction spectra of the intermediates in (b) random growth, (c) growth from the face center, and (d) growth from tips. (e) Relative peak prominence of the Ag and Au feature peaks from different growth modes. The spectra shown here were sampled every 50 steps, with the initial and final spectra. The dipole length was 1 nm, and the extinction spectra were calculated from 400 to 650 nm with an interval of 10 nm. The random growth was repeated 10 times to give statistically significant results. The edge length of the final Au@Ag octahedron was set as 20 nm.

During the growth process in each mode, the peak position in the extinction spectra corresponding to the Au composition with octahedral geometry was maintained around 550 nm with a minor shift. However, a new peak within the range of 400–450 nm emerges due to the surface coating of Ag. The variations in peak position and prominence of the Au peak along the trajectory show distinct features of different modes of Ag growth. In the first mode with a random coating process, the Au peak was monotonically red-shifted together with a decrease in the extinction; in the second mode, the extinction of the Au peak decreases first and then increases; and in the third mode, the changes are erratic with Au peak red-shifted after initial blue shift and decrease in extinction at the start followed by an increase and decrease later. During the growth process, the relative peak prominence between the Ag and Au peaks demonstrates different behavior even when the same amount of Ag was coated on the surface due to the varied surface Ag distribution (Figure 2e). The growth mode with coating starting at the tips shows the fastest increase of the prominence of the Ag peak. This result demonstrates that given the initial structure, the spectroscopic variations due to different modes in growth can be captured and potentially used to understand the growth mechanisms at the nanoscale.

On the other hand, RD-DDA is an efficient tool to scan the UV–vis spectra of a series of evolving structures, which can be used to study the optical properties of the ensembles composed of these intermediates. To simulate the UV–vis spectrum of the ensemble for the given structure distribution, every structure’s spectral pattern should be calculated, which previously was computationally inefficient. However, if the involved structures can be correlated to each other through replacement, growth, and etching, their spectra can be estimated all together through RD-DDA. For demonstration, here we consider an ensemble of the Ag@Au nanostructures where the coating of Ag happens starting at the specific sites (Figure 2c,d). The surface coverage percentage of Ag can vary in the ensemble due to possible stochastic effects like nucleation time or local concentration in the growth, which influences the population distribution of final nanostructures. The UV–vis spectra of the ensembles with various distributions can be calculated via eq 6:
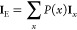
6where *P*(*x*) is the population percentage of structure *x* in the ensemble and **I**_*x*_ is its UV–vis spectrum.

As an example, we used the ensembles consisting of Ag@Au nanostructures where the Ag sites are preferably grown on the tips of the original Au octahedra. The structures in the ensemble, whose surface Ag coverages range from 0 to 1, were generated from the case whose spectra are shown in Figure 2d. Six Gaussian distributions with different means of Ag coverage and variances were used to create the ensembles, whose spectra were calculated through eq 6. The relative ratios of the individual nanostructures are proportional to the probability density function of the Gaussian distributions. They are further normalized so that the summation of the population probability is equal to 1 (Figure 3a). As shown in Figure 3b–d, the ensembles composed of nanostructures with deviations in Ag coverage showed different optical spectra compared to their counterparts with only a single nanostructure. Although trivial, the variances of the peak intensities and broadness were observed in three cases, which can be further amplified by increasing the deviations of the ensembles. This example illustrates that RD-DDA’s capability to efficiently simulate multiple UV–vis spectra of evolving nanostructures can be used not only for kinetic studies but also to formulate the spectral patterns of ensembles.

**Figure 3:**
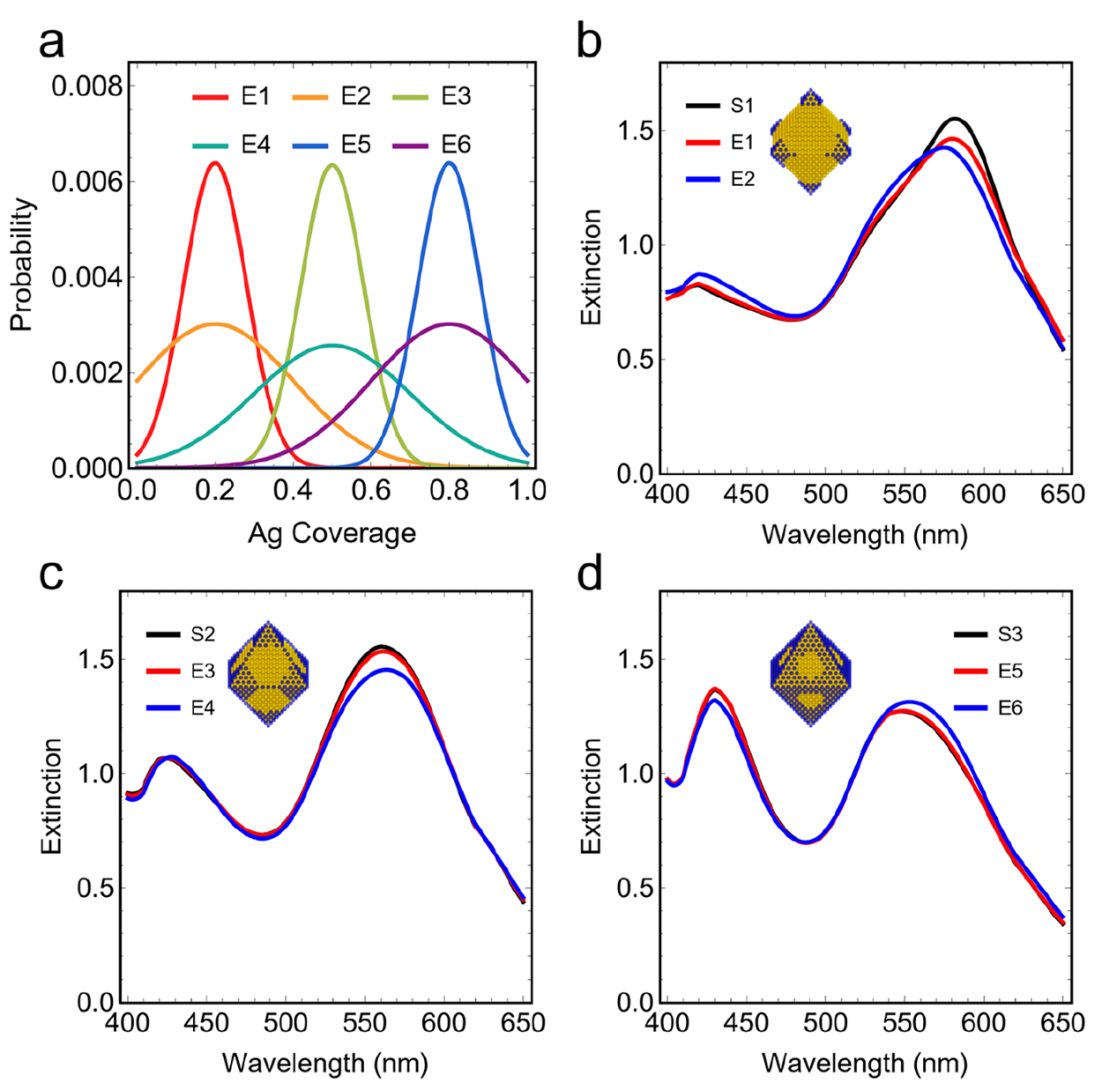
Optical spectra of the ensembles with various Ag surface coverage where Ag is preferably coated on Au octahedra starting from tips. (a) Six Gaussian distributions were used to generate the nanostructure distributions in six ensembles (E1–E6). The mean and standard deviation pairs are (0.20, 0.08), (0.20, 0.20), (0.50, 0.08), (0.50, 0.20), (0.80, 0.08), and (0.80, 0.20), respectively. (b) UV–vis spectra for the nanostructure with 0.2 surface coverage alone (S1) and the simulated spectra for ensembles 1 and 2 with a mean of 0.2 and standard deviations of 0.08 (E1) and 0.20 (E2). (c) UV–vis spectra for the nanostructure with 0.5 surface coverage (S2) and ensembles 3 and 4. (d) UV–vis spectra for the nanostructure with 0.8 surface coverage (S3) and ensembles 5 and 6. The corresponding structures of S1 to S3 are also shown in the same figure.

Empirical models can be a powerful tool to understand and approximate the growth process of nanostructures. Here we study the extinction spectra during the transformation of Au arrow-headed rods into Au octahedra using an empirical model with different growth rates for various crystallographic surfaces. The original arrow-headed rods were enclosed by two crystallographic surfaces, (110) and (111). Due to the relatively slow growth of the (111) surface, the geometry was transformed to octahedra with an increased size of arrowheads. The growth rate of individual surfaces determines the intermediate structures that the geometry must undergo through the deposition of perfect atomic layers on the previous structure. This growth process with fine resolution is equivalent to the simultaneous formation of a perfect atomic layer, which is an ideal process. However, the sequential addition of atoms on a crystallographic plane to form a single layer could be an outcome of a stochastic process. These stochastic effects and their influences on the optical properties can be captured through RD-DDA by approximating the growth process by adding dipoles representing the deposition at the atomic scale. Here the dipoles with a length of 0.41 nm (equivalent to the lattice constant of Au) were sequentially added to link the intermediates from the growth process, mimicking the island growth mode and introducing stochasticity.^31^ The intermediate structures with a perfect layered growth of dipoles are shown in Figure 4a. A set of 10 trajectories that describe the transformation of arrow-headed nanorods with a width of ca. 4 nm and a length of ca. 13 nm to Au octahedra with an edge length of ca. 9.4 nm were generated (see Video S4 for an example and SI Section 2.2 for more details).

**Figure 4:**
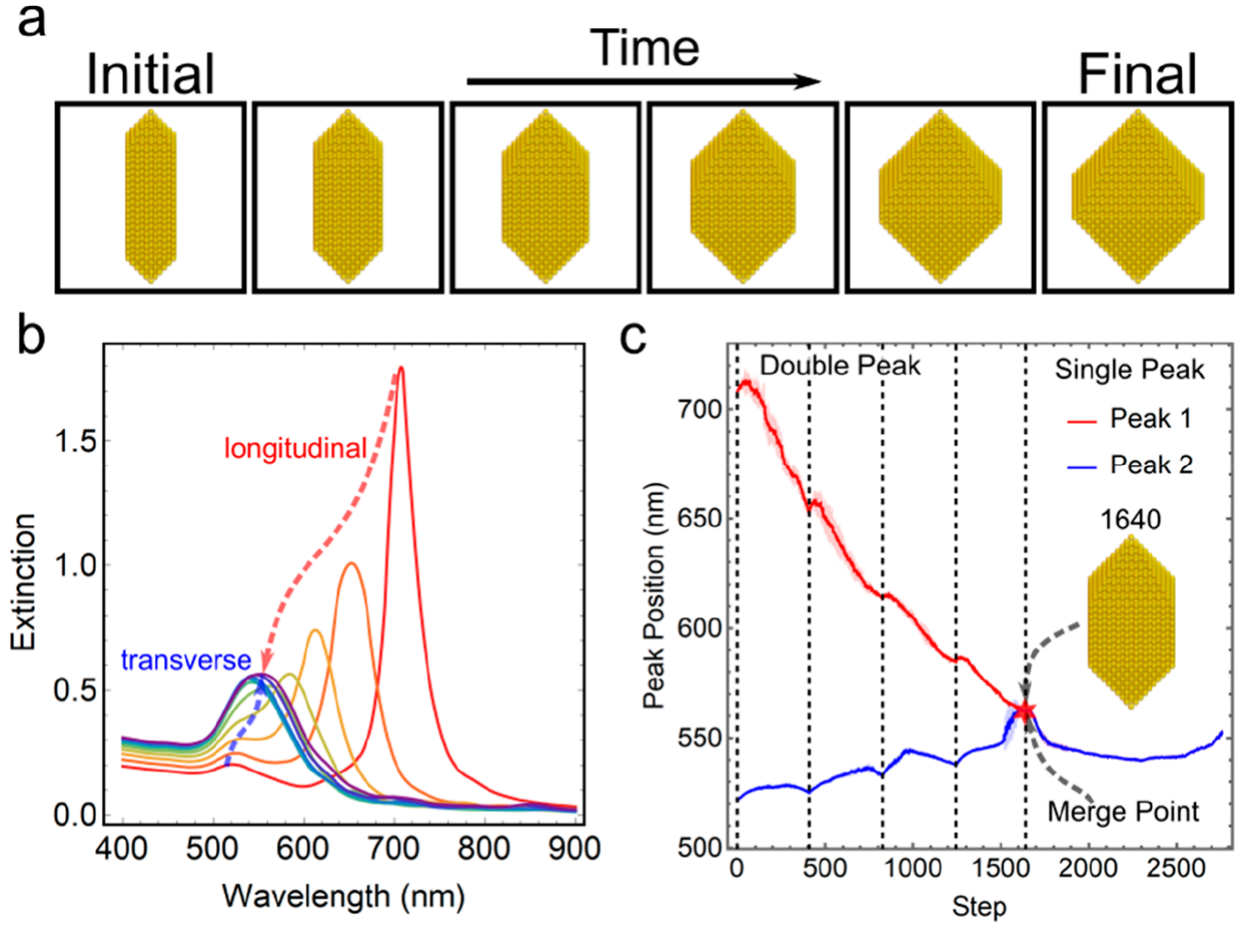
Growth of Au arrow-headed rods to Au octahedra in an empirical model. (a) Generated growth trajectory from an empirical model with stochastic effects at the atomic scale. (b) Extinction spectra corresponding to the growth process when a complete layer is formed. The spectra shown here were sampled after adding a complete layer of dipoles on the crystallographic surfaces, with the ones corresponding to the initial and final structures. The extinction spectra were calculated from 400 to 900 nm with an interval of 10 nm. (c) Shift and merging of the peak positions that initially correspond to the longitudinal (peak 1) and transverse (peak 2) modes of arrow-headed nanorods.

The corresponding changes of the UV–vis spectra were recorded for these trajectories (Figure 4b), where the steps corresponding to the intermediates with a complete layer of dipoles were labeled with a dashed line in Figure 4c. Considering the stochastic effects due to the order of dipole addition to create a complete layer along a crystallographic surface, the shift of the peaks shows variance, but the overall tendency remains consistent, as governed by the layer growth. At the earlier stage of the growth kinetics on the surface of the arrow-headed nanorods, two peaks corresponding to the longitudinal (ca. 710 nm) and transverse (ca. 520 nm) modes are observed, which is a typical characteristic of an anisotropic shape. The transformation toward isotropic octahedra leads to the addition of 2760 Au dipoles with a length of 0.41 nm, where the two peaks converge to a single peak. We divided the overall evolution of the spectra into two distinct phases corresponding to the double-peak phase and a single-peak phase. After step 1640, an intermediate with perfect layers of dipoles is formed and guarantees the merging of the two peaks at the wavelength of ca. 560 nm after the deposition of a complete layer. However, in the trajectories where stochastic effects are considered, the steps where the merging happens are different, as demonstrated by the variance of peak positions in Figure 4c around step 1500. This example demonstrates the capability of our method to predict the tendency of spectral fingerprints with the slight nanoscale changes for a given nanostructure as well as to estimate the expected bounds on the spectral variance due to the stochastic nature of the growth process.

In general, the crystal growth process can be represented by two distinct types of models based on underlying spatiotemporal scales: continuum models representing a rapid growth process leading to the formation of a complete layer on a crystallographic plane or discrete atomic-scale models tracking individual atomic processes during the structural transformation. To further demonstrate the capability of our method to calculate the spectral properties during the structural transformation defined by an atomic model, a kinetic Monte Carlo simulation was employed to describe the morphological evolution of Au nanostructures^28,30^ (see SI Section 2.3 for complete details).

The model captures both specific structural changes and the stochastic effects during the growth process. For face-centered cubic (FCC) crystals, at each step, the acceptance probability to add or remove the atom was calculated by considering (1) the binding energy of an atom to its nearest-neighbor atoms and (2) the difference between the chemical potentials of the nanocrystal and the solution phase.^28^ Previously, similar Monte Carlo simulations have been performed to investigate the evolution from Au octahedra to ultrasmooth Au nanospheres.^30^ Here we combined the proposed RD-DDA with kinetic Monte Carlo simulations to track the spectral properties during the etching process of Au octahedra to form nanospheres. Furthermore, we expand the atomic model from a single metal system such as pure Au nanostructures to the bimetallic Au/M nanostructures, where M represents the second metal type. The *in situ* tracking of the spectral properties of emerging structures not only proves the generality of our method but also offers insights into the unique spectral signals of the intermediates that were not possible to observe directly.

First, we implemented the kinetic Monte Carlo model to study the etching process of Au octahedra. The Au octahedra have an initial edge length of ca. 9.3 nm and are enclosed with eight (111) surfaces. The atomic model considers the addition or removal of a single Au atom (see SI Section 2.3), while the resolution of the structural transformation for the scattering simulation is coarse-grained to a single FCC lattice (length of 0.41 nm) representing a dipole. If the lattice is fully occupied, the dipole volume represents the Au dipole; otherwise it is considered to be a medium dipole. To capture the effects of stochasticity, three repeats of each simulation with the same parameters were performed by varying the random seed. In all three runs, the sharp edges/tips of the octahedron were smoothed out, and a cuboctahedral–spherical nanostructure was formed, with a further reduction in the size and increase of the surface roughness (see Figure 5a). The RD-DDA was coupled with the Monte Carlo simulation to track the UV–vis change in this process (see Figure 5b for an example). The simulation revealed a consistent shift in the peak position and a decrease in the extinction coefficients among all three repeats due to the reduction in the size of the nanoparticle (see Figure 5c). However, local maxima of peak positions and the extinction factor were also observed during the etching process, where the geometry distortion and small features that emerged on the surface can shift the peak position and enhance the extinction coefficient (see Videos S6 and S7 for an example of the overall trajectory and the corresponding extinction spectra).

**Figure 5:**
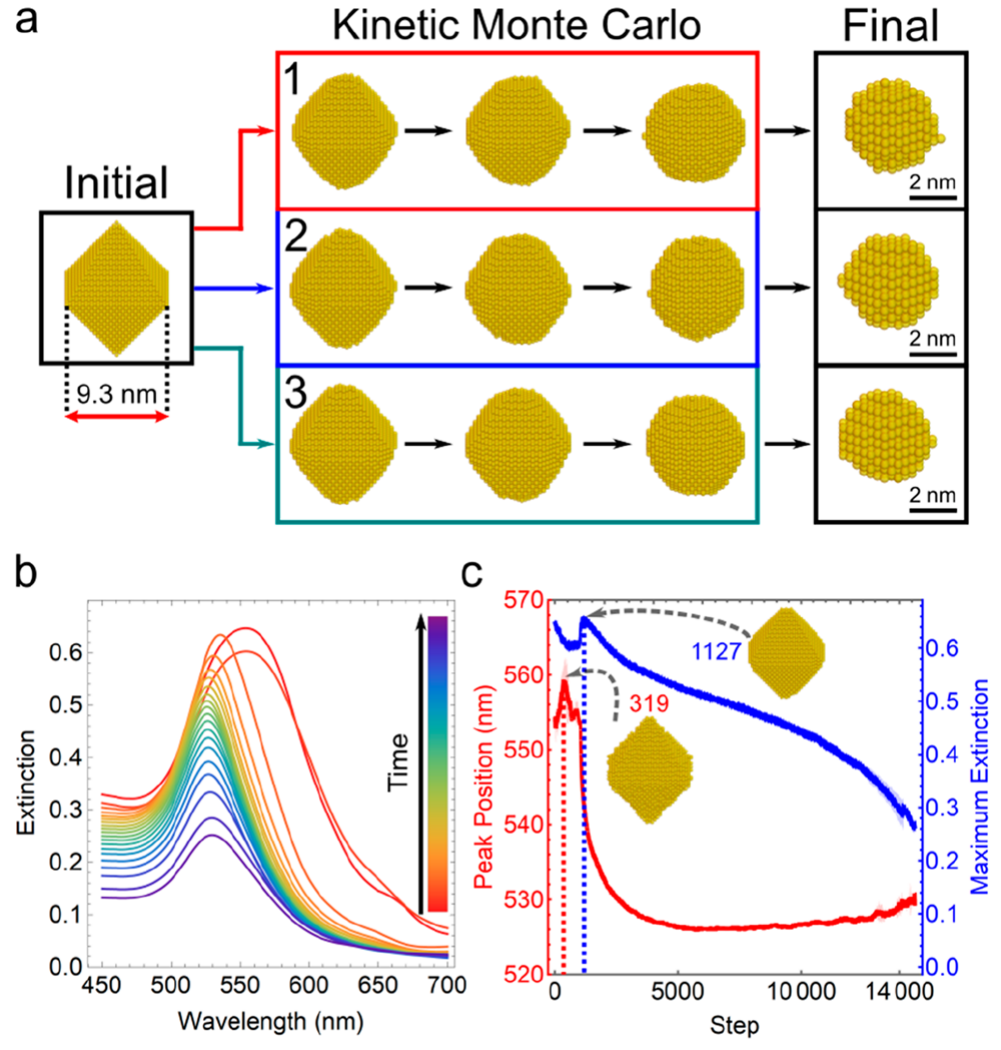
Etching of Au octahedra to Au nanospheres in the kinetic Monte Carlo simulation. (a) Three repeatedly generated etching trajectories from the kinetic Monte Carlo model with the same parameters. The dipole length is set the same as the lattice constant of Au (0.41 nm). (b) Extinction spectra during the etching process corresponding to the first trajectory. The spectra were sampled every 800 steps together with the initial and final spectra. (c) Shift of the peak position and variation of the peak intensity from the three trajectories. The average values of the peak positions and the corresponding extinction coefficients among the three trajectories are shown in red and blue lines. The boundaries defined by mean ± standard deviation are shown in the transparent lines. During the structural transformation in the first trajectory, at step 319 and 1127, the maximum peak position and the maximum extinction signal are observed, respectively.

To further demonstrate the generality of RD-DDA in coupling with more complex atomic models, we explored the structural transformation of Au nanorods with a second type of metal M under various growth conditions. By varying the chemical potentials of Au and M as well as the relative Au–M and M–M binding energies with respect to the Au–Au bond, we observed growth, etching, and equilibrium processes leading to various bimetallic structures, including rods, bipyramids, and ellipsoids with different surface structures (see Figure 6a for examples). Principal component analysis (PCA) shows the large variance of the surface structures on the different kinetic parameters (Figure 6b–d), indicating that the optical properties of the final structures can be very distinct from each other as well as compared to the initial Au nanorods. Similar to the single metal system, the temporal evolution of the spectral signal under the kinetic Monte Carlo simulation can be captured using RD-DDA. The atomic distribution and the physical properties of the second metal M also influence the optical properties of the bimetallic nanostructures.

**Figure 6:**
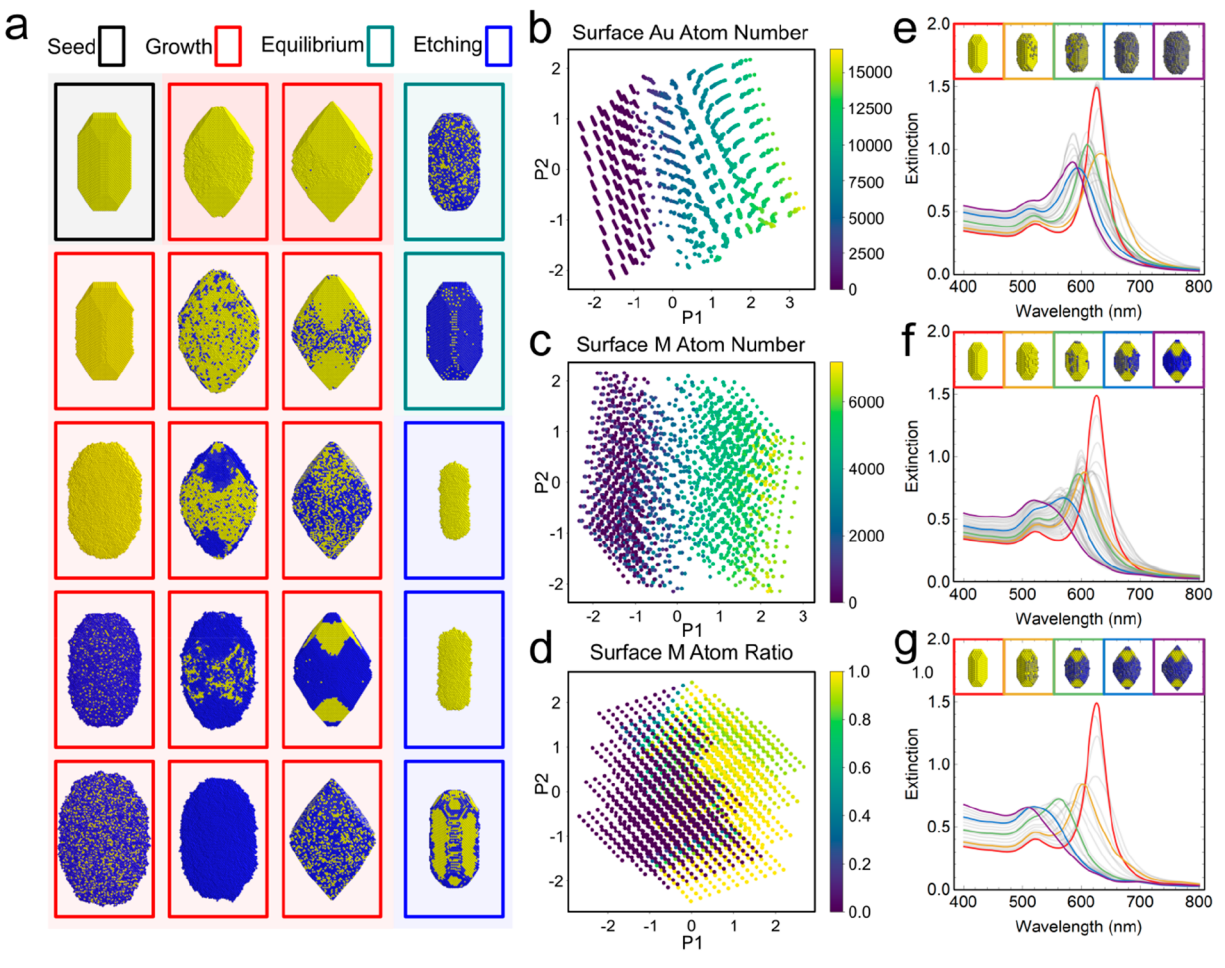
Kinetic Monte Carlo simulation on Au–M bimetallic system. (a) Initial Au nanorod seed (black) and emerged structures after 500,000 kinetic Monte Carlo steps with different binding energies and chemical potentials. Au and M are represented by yellow and blue spheres. The new structure can form from the overgrowth (red), etching (blue) and equilibrium (green) with the original seed. (b, c) Numbers of surface Au and M atoms and (d) percentages of M on the surface under various kinetic parameters after the principal component analysis (PCA) with two components (labeled as P1 and P2). PCA analysis was performed on the data including the kinetic parameters and the interested surface property to show structural variance. The nanostructures that are completely etched away after 500,000 steps were not analyzed here. (e–g) Simulated spectral evolution during the formation of homogeneously mixed nanorods (e) and bipyramids with different phase mixing degrees on the surface (f, g) by attributing the second metal M as Ag.

As a demonstration, we selected three trajectories, including the formation of a homogeneously mixed nanorod and two bipyramids with different atomic distributions of the two metals on the surface. The RD-DDA simulations were performed by assigning the second metal as Ag. For coarse-graining from atoms to dipoles, a dipole length was set to 1.5 times the lattice constant of FCC Au. If all the atom sites within the dipole are fully occupied, its polarizability is calculated by the weighted average refractive index of Au and M atoms within the dipole; otherwise it is set to the polarizability of the medium. During the formation of the homogeneously mixed nanorods, the longitudinal peak from the original nanorod seed is blue-shifted due to the decrease of the aspect ratio and the increase of the Ag coverage on the surface (Figure 6e). In the case of the formation of bipyramids, Ag atoms can selectively deposit on the (100) surface, causing the geometry to transform from rods to bipyramids and increasing the Ag surface coverage. These two factors induced the blue shift of the longitudinal peak and the final merge of the transverse and longitudinal peaks. Despite similar spectral tendencies in forming bipyramids, the UV–vis change in the growth process can be distinct by different distributions of the atoms, as indicated by the gray lines in Figure 6f,g. Furthermore, due to the larger amount of Ag after the growth, the final peak in Figure 6g is more blue-shifted without a shoulder peak. These results demonstrate that RA-DDA is a versatile and powerful tool to capture and quantify the influence of nanoscale transformations on the optical signals in atomic growth models.

We have developed and implemented a fast and computationally efficient rank-one decomposition accelerated discrete-dipole approximation (RD-DDA) method to investigate the optical properties of dynamically evolving nanostructures. This method, scalable to large systems, was benchmarked against the standard DDA and has shown an acceleration in the performance by a factor of several hundreds for a system consisting of ca. 4000 dipoles. We applied RD-DDA to efficiently track the extinction spectra during nano/atomic-scale structural transformations in the case of custom-built trajectories capturing the variance in the spectral features of different growth modes during the formation of Ag@Au octahedra. This was further expanded to track the spectral changes over ensembles with varying population distributions of nanostructures. As a key application, RD-DDA was combined with continuum and atomic-scale methods to investigate the influence of nanostructural morphological changes and stochasticity on the extinction spectra. The proposed method is versatile and can be employed for a range of applications such as application-specific morphological optimization of nanostructures and understanding growth mechanisms and kinetics at the nanoscale when combined with experimental data.

## Data Availability

The data used in this work are available upon reasonable request to the corresponding author via Lee.Cronin@glasgow.ac.uk. The developed Python package and the original codes to generate the data in this work are available at https://github.com/croningp/RD-DDA. The basic implementation of DDA (directly solving the linear equations) was based on our previous work.^32^

## Supporting Information Available

Random growth of a layer of Ag on the surface of Au octahedra and its corresponding extinction spectra (Video S1); growth of a layer of Ag from the face centers on the surface of Au octahedra and its corresponding extinction spectra (Video S2); growth of a layer of Ag from the tips on the surface of Au octahedra and its corresponding extinction spectra (Video S3); growth of Au arrow-headed rods to Au octahedra using an empirical model (Video S4); extinction spectra in the growth of Au arrow-headed rods to Au octahedra (Video S5); etching of Au octahedra to Au nanospheres using a kinetic Monte Carlo model (Video S6); extinction spectra in the etching of Au octahedra to Au nanospheres (Video S7) (ZIP)
Full information about the derivation of the rank-one decomposition method with validations of accuracy and computational efficiency (Section 1) and details to generate the trajectories, simulate the spectral properties of the intermediates, and analyze the extinction spectra (Section 2) (PDF)
Transparent Peer Review report available (PDF)
