# Supplementary material for: An Accelerated Method for Investigating Spectral Properties of Dynamically Evolving Nanostructures
Source: J Phys Chem Lett. 2023 Apr 20;14(16):3929–38. doi: 10.1021/acs.jpclett.3c00395 (PMC10150391; doi:10.1021/acs.jpclett.3c00395)
Supplement: Supplementary file 2 — jz3c00395_si_002.pdf [file jz3c00395_si_002.pdf]

Supplementary Information

An Accelerated Method for Investigating Spectral Properties of Dynamically Evolving Nanostructures

Yibin Jiang[1†], Abhishek Sharma[1†], and Leroy Cronin[1]*

[1]School of Chemistry, University of Glasgow, University Avenue, Glasgow G12 8QQ, UK.

[†]Equal contribution. *Corresponding author email: Lee.Cronin@glasgow.ac.uk

Table of Contents

1. Discrete-dipole approximation ... 3

1.1. Method ... 3

1.2. Formulating new solutions when the polarizability of a dipole is changed ... 6

1.3. Formulating new solutions when a dipole is added ... 10

1.3.1. Problem ... 10

1.3.2. Method ... 12

1.4. Formulating new solutions when a dipole is removed ... 15

1.4.1. Problem ... 15

1.4.2. Method ... 17

1.5. The validation and numerical approximation for replacement, growth, and etching ... 19

1.6. The benchmark of the computational time for the direct solutions from DDA and the iterative solutions from RD-DDA ... 29

1.7. Estimating the time cost of the direct solution method and RD-DDA ... 33

2. Efficient tracking of UV-Vis spectra using the RD-DDA ... 36

2.1. Custom-built trajectories ... 36

2.2. An empirical crystallographic surface growth model ... 36

2.3. Atomic-scale models of nanostructural transformation using kinetic Monte Carlo simulation 40

2.3.1. Au nanostructures ... 40

2.3.2. Au-M nanostructures ... 42

3. References..45

Supplementary Video S1: The random growth of a layer of Ag on the surface of Au octahedra and its corresponding extinction spectra.

Supplementary Video S2: The growth of a layer of Ag from the face centres on the surface of Au octahedra and its corresponding extinction spectra.

Supplementary Video S3: The growth of a layer of Ag from the tips on the surface of Au octahedra and its corresponding extinction spectra.

Supplementary Video S4: The growth of Au arrow-headed rods to Au octahedra using an empirical model.

Supplementary Video S5: The extinction spectra in the growth of Au arrow-headed rods to Au octahedra.

Supplementary Video S6: The etching of Au octahedra to Au nanospheres using a kinetic Monte Carlo model.

Supplementary Video S7: The extinction spectra in the etching of Au octahedra to Au nanospheres.

1. Discrete-dipole approximation

1.1. Method

In the discrete-dipole approximation (DDA)[1–3], the geometry is discretized into N polarizable cubic lattices which represent the point dipoles. Every point dipole's polarizability is associated with its local dielectric constant. The polarization of the jth dipole is induced by the electric field from the incident beam and also the rest $(N-1)$ dipoles (Eq. (1)-(2)).

$$\mathbf{P}_j = \boldsymbol{\alpha}_j \mathbf{E}_j \tag{1}$$

where $\mathbf{P}_j$ is the polarization of the jth dipole, $\boldsymbol{\alpha}_j$ is the jth dipole's polarizability and $\mathbf{E}_j$ denotes the electric field at the jth dipole's position $\boldsymbol{r}_j$ which is defined in Eq. (2):

$$\mathbf{E}_j = \mathbf{E}_{j,inc} + \sum_{k \neq j}^{N} \mathbf{E}_k \tag{2}$$

where $\mathbf{E}_{j,inc}$ is the electric field of the incident beam at the location $\boldsymbol{r}_j$ and $\mathbf{E}_k$ is the contribution from the kth dipole located at $\boldsymbol{r}_k$. They can be formulated as below:

$$\mathbf{E}_{j,inc} = \mathrm{E}_{inc} e^{i\boldsymbol{k}\cdot\boldsymbol{r}_j} \tag{3}$$

$$\mathbf{E}_k = -\frac{e^{ikr_{j,k}}}{{r_{j,k}}^3}\left\{k^2\boldsymbol{r}_{j,k} \times \left(\boldsymbol{r}_{j,k} \times \mathbf{P}_k\right) + \left(\frac{1 - ikr_{j,k}}{{r_{j,k}}^2}\right) \times \left[{r_{j,k}}^2\mathbf{P}_k - 3\boldsymbol{r}_{j,k}\left(\boldsymbol{r}_{j,k} \cdot \mathbf{P}_k\right)\right]\right\} \tag{4}$$

where i is the imaginary number, E_{inc} is the amplitude of the incident beam, $\boldsymbol{k}$ is the wavenumber with the same direction of the wave propagation and its amplitude is defined as $k = \frac{2\pi}{\lambda}$, where λ is the wavelength of the incident beam, $\boldsymbol{r}_{j,k} \equiv \boldsymbol{r}_j - \boldsymbol{r}_k$, $r_{j,k} \equiv \left|r_{j,k}\right|$, and $\mathbf{P}_k$ is the polarization of the kth dipole.

To calculate the polarizations in a self-consistent manner, the system can be described by simplifying the Maxwell equations into a set of linear equations (Eq. (5)).

$$\mathbf{AP} = \mathbf{E} \tag{5}$$

where $\mathbf{E}$ is a $3N$ vector describing the local electric field of the incident wave in every dipole position, $\mathbf{A}$ is a $3N \times 3N$ symmetric matrix depending on the geometry and materials of the geometry. $\mathbf{P}$ is a $3N$ vector representing the polarizations of the dipoles. $\mathbf{A}$ is composed of a series of 3×3 matrices ($\mathbf{A}_{j,k}$) as shown in Eq. (6).

$$\mathbf{A} = \begin{bmatrix} \mathbf{A}_{1,1} & \cdots & \mathbf{A}_{1,N} \\ \vdots & \ddots & \vdots \\ \mathbf{A}_{N,1} & \cdots & \mathbf{A}_{N,N} \end{bmatrix} \tag{6}$$

For any matrix $\mathbf{A}_{j,j}$ at the diagonal position, it is defined by the inverse of the polarizabilities ($\alpha_{j,X}$, $\alpha_{j,Y}$, $\alpha_{j,Z}$) of the jth dipole in X, Y and Z directions (Eq. (7)). For simplicity, it is generally assumed that the dipole is isotropic in the X, Y and Z directions, so that $\alpha_{j,X} = \alpha_{j,Y} = \alpha_{j,Z} = \alpha_j$.

$$\mathbf{A}_{j,j} = \begin{bmatrix} {\alpha_{j,X}}^{-1} & 0 & 0 \\ 0 & {\alpha_{j,Y}}^{-1} & 0 \\ 0 & 0 & {\alpha_{j,Z}}^{-1} \end{bmatrix} \tag{7}$$

For the matrix $\mathbf{A}_{j,k\neq j}$ that is off-diagonal, it is defined by the relative distance between the jth and k^{th} dipoles (Eq. (8)).

$$\mathbf{A}_{\mathrm{j,k}} = \frac{\exp(ikr_{j,k})}{r_{j,k}} \left(k^2 \left(\hat{\boldsymbol{r}}_{\boldsymbol{j,k}} (\hat{\boldsymbol{r}}_{\boldsymbol{j,k}})^{\mathbf{T}} - \mathbf{1_3} \right) + \frac{(1 - ikr_{j,k})}{{r_{j,k}}^2} \left(3\hat{\boldsymbol{r}}_{\boldsymbol{j,k}} (\hat{\boldsymbol{r}}_{\boldsymbol{j,k}})^{\mathbf{T}} - \mathbf{1_3} \right) \right) \tag{8}$$

where $\hat{\boldsymbol{r}}_{\boldsymbol{j,k}} = \frac{r_j - r_k}{|r_{j,k}|}$ and $\mathbf{1_3}$ is the 3×3 identity matrix. It should be noted these off-diagonal matrices are independent of the polarizability of the dipoles, and thus are kept constant when the properties of the dipoles are changed.

After calculating $\mathbf{P}$, we can further evaluate the extinction and absorption cross-sections via the following Eq. (9) and Eq. (10), respectively.

$$\mathrm{C}_{ext} = \frac{4\pi k}{|\mathrm{E}_{inc}|^2} \sum_{j}^{N} \mathrm{Im}\left({\mathbf{E}_{inc,j}}^{*} \cdot \mathbf{P}_j \right) \tag{9}$$

where C_{ext} is the extinction cross-section, $\mathrm{Im}(x)$ denotes the imaginary part of x and x^* is the conjugate of x.

$$\mathrm{C}_{abs} = \frac{4\pi k}{|\mathrm{E}_{inc}|^2} \sum_{j}^{N} \left\{ \mathrm{Im}\left[\mathbf{P}_j \cdot \left({\alpha_j}^{-1} \right)^{*} {\mathbf{P}_j}^{*} \right] - \frac{2}{3} k^3 \mathbf{P}_j \cdot {\mathbf{P}_j}^{*} \right\} \tag{10}$$

where C_{abs} is the absorption cross-section and α_j is the polarizability of the jth dipole.

The "filtered coupled dipole" (FCD)[4,5] method was implemented to calculate the polarizability of a dipole in this work (Eq. (11)-(13)).

$$\alpha_j = \frac{\alpha_{j,CM}}{1 + D} \tag{11}$$

where $\alpha_{j,CM}$ is the Clausius-Mossotti polarizability[2] defined as below:

$$\alpha_{j,CM} = \frac{3d^3}{4\pi} \frac{\left(m_j{}^2 - 1\right)}{\left(m_j{}^2 + 2\right)} \tag{12}$$

where m_j is the complex refractive index of the jth dipole and d is the dipole length. The D term in Eq. (11) is defined as:

$$D = \frac{\alpha_{j,CM}}{d^3} \left[\frac{4}{3} (kd)^2 + \frac{2}{3\pi} \ln \left(\frac{\pi - kd}{\pi + kd} \right) (kd)^3 + \frac{2}{3} i (kd)^3 \right] \tag{13}$$

where d is the dipole length. If not mentioned, the refractive indexes used in this work were all from reference[6].

Once C_{ext} and C_{abs} are calculated, the extinction and absorption efficiency factors can be calculated from the corresponding cross-sections via Eq. (14).

$$Q_{ext/abs} = \frac{C_{ext/abs}}{\pi r_{eff}{}^2} \tag{14}$$

where r_{eff} is the effective radius of a sphere with the same volume as the geometry, which can be defined by $\frac{4\pi r_{eff}{}^3}{3} = Nd^3$, where N is the number of dipoles and d is the dipole length.

During the study of the spectral properties of nanostructures, the extinction efficiency factors by uniformly sampling 1000 possible orientations of the simulated nanoparticle with respect to the incident beam are calculated and averaged to give the final UV-Vis spectrum. To enable accuracy, the validation criteria (Eq. (15)) should always be satisfied.

$$|m|kd < 0.5 \tag{15}$$

where m is the complex refractive index of the material, k is the wavenumber of the incident beam and d is the dipole length.

Based on the DDA described above, how the polarizations will change when the replacement/addition/removal of the dipoles in the geometry happens will be discussed below, as indicated in **Figure S1**.

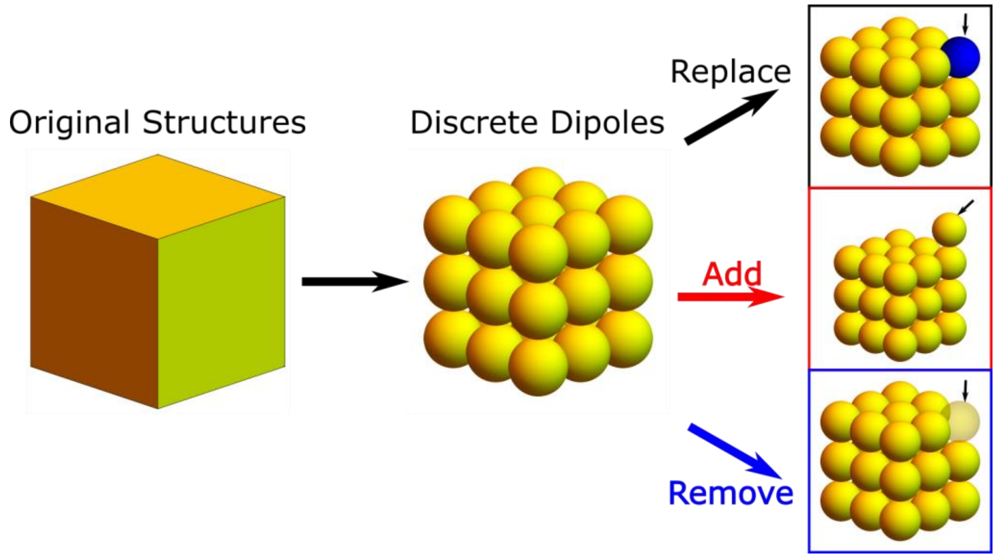

Figure S1. The scheme for the discrete-dipole approximation. The original geometry is represented by a series of discrete dipoles. The replacement (indicated by changing the colour of a dipole from yellow to blue), addition or removal (indicated by making one dipole transparent) of the dipoles from the system will change the polarizations of the dipoles.

1.2. Formulating new solutions when the polarizability of a dipole is changed

A replacement reaction within the nanostructure can be represented by the change in the properties of the dipoles. Here, the relationship between the new solution of dipole polarizations with the original solution before the replacement is derived. Eq. (5) can be reformulated as below to calculate the polarizations:

$$\mathbf{P} = \mathbf{A}^{-1}\,\mathbf{E} \tag{16}$$

where $\mathbf{A}^{-1}$ is the inverse of $\mathbf{A}$. Changing the property of a dipole is equivalent to updating its polarizability tensor $\boldsymbol{\alpha}$, where the diagonal elements of $\mathbf{A}$ are updated. The off-diagonal elements are kept the same as indicated by Eq. (8). The diagonal part of $\mathbf{A}$ that is only dependent on the polarizability and regardless of the geometry can be expressed as follows:

$$\mathbf{A} = \begin{bmatrix} \alpha_1{}^{-1} & 0 & 0 & & & & & & \\ 0 & \alpha_1{}^{-1} & 0 & & \cdots & & & \cdots & \\ 0 & 0 & \alpha_1{}^{-1} & & & & & & \\ & & & \alpha_j{}^{-1} & 0 & 0 & & & \\ & \vdots & & 0 & \alpha_j{}^{-1} & 0 & & \vdots & \\ & & & 0 & 0 & \alpha_j{}^{-1} & & & \\ & & & & & & \alpha_N{}^{-1} & 0 & 0 \\ & \cdots & & & \cdots & & 0 & \alpha_N{}^{-1} & 0 \\ & & & & & & 0 & 0 & \alpha_N{}^{-1} \end{bmatrix} \tag{17}$$

The off-diagonal blocks are independent of polarizability tensors, thus they are not shown here. Also,

for simplicity, it is assumed the dipoles are isotropic in the following derivation. However, the formulation is general and similar expressions can be derived for anisotropic dipoles where only the diagonal elements exist but their values are different.

Next, assume the property of dipole $\mathbf{P}_j$ is changed so that it has a new polarizability $\alpha_j{}'$, then the new matrix of $\mathbf{A}'$ will be:

$$\mathbf{A}' = \begin{bmatrix} \alpha_1{}^{-1} & 0 & 0 & & & & & & \\ 0 & \alpha_1{}^{-1} & 0 & & \cdots & & & \cdots & \\ 0 & 0 & \alpha_1{}^{-1} & & & & & & \\ & & & \alpha_j{}'^{-1} & 0 & 0 & & & \\ & \vdots & & 0 & \alpha_j{}'^{-1} & 0 & & \vdots & \\ & & & 0 & 0 & \alpha_j{}'^{-1} & & & \\ & & & & & & \alpha_N{}^{-1} & 0 & 0 \\ & \cdots & & & \cdots & & 0 & \alpha_N{}^{-1} & 0 \\ & & & & & & 0 & 0 & \alpha_N{}^{-1} \end{bmatrix} \quad (18)$$

where $\alpha_j{}'$ is the new polarizability of dipole $\mathbf{P}_j$.

Calculating the inverse matrix of $\mathbf{A}'$ directly is straightforward but computationally inefficient. Instead, a method which can analytically describe the relationship between the original solution and the updated solution should be derived. The new inverse matrix $\mathbf{A}'^{-1}$ would be written as a function of the original inverse matrix $\mathbf{A}^{-1}$. To do so, **Lemma 1**[7] was introduced:

Lemma 1. If **A** and **A**+**B** are invertible, and **B** has rank one, then let $g = \mathrm{tr}(\mathbf{B}\mathbf{A}^{-1})$. If $g \neq -1$, we have $(\mathbf{A} + \mathbf{B})^{-1} = \mathbf{A}^{-1} - \frac{1}{1+g}\mathbf{A}^{-1}\mathbf{B}\mathbf{A}^{-1}$.

By comparing Eq. (17) and (18), we can write:

$$\mathbf{A}' = \mathbf{A} + \mathbf{B}_X + \mathbf{B}_Y + \mathbf{B}_Z$$

$$= \mathbf{A} + \begin{bmatrix} 0 & 0 & 0 & & & & & & \\ 0 & 0 & 0 & & \cdots & & & \cdots & \\ 0 & 0 & 0 & & & & & & \\ & & & \alpha_j{}'^{-1} - \alpha_j{}^{-1} & 0 & 0 & & & \\ & \vdots & & 0 & \alpha_j{}'^{-1} - \alpha_j{}^{-1} & 0 & & \vdots & \\ & & & 0 & 0 & \alpha_j{}'^{-1} - \alpha_j{}^{-1} & & & \\ & & & & & & 0 & 0 & 0 \\ & \cdots & & & \cdots & & 0 & 0 & 0 \\ & & & & & & 0 & 0 & 0 \end{bmatrix}$$

$$
\begin{aligned}
&= \mathbf{A} + \begin{bmatrix}
0 & 0 & 0 & & & & & & \\
0 & 0 & 0 & & \cdots & & & \cdots & \\
0 & 0 & 0 & & & & & & \\
 & & & {\alpha_j{}'}^{-1} - {\alpha_j}^{-1} & 0 & 0 & & & \\
 & \vdots & & 0 & 0 & 0 & & \vdots & \\
 & & & 0 & 0 & 0 & & & \\
 & & & & & & 0 & 0 & 0 \\
 & \cdots & & & \cdots & & 0 & 0 & 0 \\
 & & & & & & 0 & 0 & 0
\end{bmatrix} \\
&+ \begin{bmatrix}
0 & 0 & 0 & & & & & & \\
0 & 0 & 0 & & \cdots & & & \cdots & \\
0 & 0 & 0 & & & & & & \\
 & & & 0 & 0 & 0 & & & \\
 & \vdots & & 0 & {\alpha_j{}'}^{-1} - {\alpha_j}^{-1} & 0 & & \vdots & \\
 & & & 0 & 0 & 0 & & & \\
 & & & & & & 0 & 0 & 0 \\
 & \cdots & & & \cdots & & 0 & 0 & 0 \\
 & & & & & & 0 & 0 & 0
\end{bmatrix} \\
&+ \begin{bmatrix}
0 & 0 & 0 & & & & & & \\
0 & 0 & 0 & & \cdots & & & \cdots & \\
0 & 0 & 0 & & & & & & \\
 & & & 0 & 0 & 0 & & & \\
 & \vdots & & 0 & 0 & 0 & & \vdots & \\
 & & & 0 & 0 & {\alpha_j{}'}^{-1} - {\alpha_j}^{-1} & & & \\
 & & & & & & 0 & 0 & 0 \\
 & \cdots & & & \cdots & & 0 & 0 & 0 \\
 & & & & & & 0 & 0 & 0
\end{bmatrix}
\end{aligned}
\tag{19}
$$

By writing so, we can define $\mathbf{A}'$ as the summation of $\mathbf{A}$ and three rank-one matrices:

$$
\mathbf{A}' = \mathbf{A} + \mathbf{B}_X + \mathbf{B}_Y + \mathbf{B}_Z \tag{20}
$$

where $\mathbf{B}_X$, $\mathbf{B}_Y$ and $\mathbf{B}_Z$ correspond to the sparse matrices with only one non-zero element of ${\alpha_j{}'}^{-1} - {\alpha_j}^{-1}$ in Eq. (19). For anisotropic dipoles where only the diagonal elements exist but with different values, the elements of $\mathbf{B}_X$, $\mathbf{B}_Y$ and $\mathbf{B}_Z$ should be calculated in every corresponding direction of X, Y and Z respectively.

With **Lemma 1**, the next step is to add the first rank-one matrix ($\mathbf{B}_X$), whose physical significance is to change the polarizability of the jth dipole from α_j to $\alpha_j{}'$ in the X direction. Its corresponding inverse matrix ${(\mathbf{A}')_x}^{-\mathbf{1}}$ can be calculated as:

$$
\begin{aligned}
(\mathbf{A}')_x{}^{-\mathbf{1}} &= \mathbf{A}^{-\mathbf{1}} - \frac{1}{1+\mathrm{g}}\mathbf{A}^{-\mathbf{1}}\mathbf{B}_X\mathbf{A}^{-\mathbf{1}} \\
&= \mathbf{A}^{-\mathbf{1}} - \frac{1}{1+\mathrm{g}}\mathbf{A}^{-\mathbf{1}}
\begin{bmatrix}
0 & 0 & 0 & & & & & & \\
0 & 0 & 0 & & \cdots & & & \cdots & \\
0 & 0 & 0 & & & & & & \\
 & & & \alpha_j{}'^{-1} - \alpha_j{}^{-1} & 0 & 0 & & & \\
 & \vdots & & 0 & 0 & 0 & & \vdots & \\
 & & & 0 & 0 & 0 & & & \\
 & & & & & & 0 & 0 & 0 \\
 & \cdots & & & \cdots & & 0 & 0 & 0 \\
 & & & & & & 0 & 0 & 0
\end{bmatrix}
\mathbf{A}^{-\mathbf{1}} \\
&= \mathbf{A}^{-\mathbf{1}} - \frac{\alpha_j{}'^{-1} - \alpha_j{}^{-1}}{1 + \left(\alpha_j{}'^{-1} - \alpha_j{}^{-1}\right)(\mathbf{A}^{-\mathbf{1}})_{3j-2,3j-2}}
\begin{bmatrix}
(\mathbf{A}^{-\mathbf{1}})_{1,3j-2} \\
(\mathbf{A}^{-\mathbf{1}})_{2,3j-2} \\
(\mathbf{A}^{-\mathbf{1}})_{3,3j-2} \\
\vdots \\
(\mathbf{A}^{-\mathbf{1}})_{N,3j-2}
\end{bmatrix}
\begin{bmatrix}
(\mathbf{A}^{-\mathbf{1}})_{1,3j-2} \\
(\mathbf{A}^{-\mathbf{1}})_{2,3j-2} \\
(\mathbf{A}^{-\mathbf{1}})_{3,3j-2} \\
\vdots \\
(\mathbf{A}^{-\mathbf{1}})_{N,3j-2}
\end{bmatrix}^{\mathrm{T}}
\end{aligned}
\tag{21}
$$

where, $(\mathbf{A}^{-\mathbf{1}})_{j,k}$ is the element of $\mathbf{A}^{-\mathbf{1}}$ at the jth row and the kth column. Then for the new solution to the polarizations of dipoles $(\mathbf{P}')_x$, it can be written as:

$$
\begin{aligned}
(\mathbf{P}')_x &= (\mathbf{A}')_x{}^{-\mathbf{1}}\mathbf{E} \\
&= \left(\mathbf{A}^{-\mathbf{1}} - \frac{1}{1+\mathrm{g}}\mathbf{A}^{-\mathbf{1}}
\begin{bmatrix}
0 & 0 & 0 & & & & & & \\
0 & 0 & 0 & & \cdots & & & \cdots & \\
0 & 0 & 0 & & & & & & \\
 & & & \alpha_j{}'^{-1} - \alpha_j{}^{-1} & 0 & 0 & & & \\
 & \vdots & & 0 & 0 & 0 & & \vdots & \\
 & & & 0 & 0 & 0 & & & \\
 & & & & & & 0 & 0 & 0 \\
 & \cdots & & & \cdots & & 0 & 0 & 0 \\
 & & & & & & 0 & 0 & 0
\end{bmatrix}
\mathbf{A}^{-\mathbf{1}} \right) \mathbf{E} \\
&= \mathbf{A}^{-\mathbf{1}}\mathbf{E} - \frac{\alpha_j{}'^{-1} - \alpha_j{}^{-1}}{1 + \left(\alpha_j{}'^{-1} - \alpha_j{}^{-1}\right)(\mathbf{A}^{-\mathbf{1}})_{3j-2,3j-2}}
\begin{bmatrix}
(\mathbf{A}^{-\mathbf{1}})_{1,3j-2} \\
(\mathbf{A}^{-\mathbf{1}})_{2,3j-2} \\
\cdots \\
\cdots \\
(\mathbf{A}^{-\mathbf{1}})_{N,3j-2}
\end{bmatrix}
\mathbf{P}_{3j-2} \\
&= \mathbf{P} - \frac{\alpha_j{}'^{-1} - \alpha_j{}^{-1}}{1 + \left(\alpha_j{}'^{-1} - \alpha_j{}^{-1}\right)(\mathbf{A}^{-\mathbf{1}})_{3j-2,3j-2}}
\begin{bmatrix}
(\mathbf{A}^{-\mathbf{1}})_{1,3j-2} \\
(\mathbf{A}^{-\mathbf{1}})_{2,3j-2} \\
\cdots \\
\cdots \\
(\mathbf{A}^{-\mathbf{1}})_{N,3j-2}
\end{bmatrix}
\mathbf{P}_{3j-2}
\end{aligned}
\tag{22}
$$

where $\mathbf{P}$ is a vector of 3N elements that defines the polarizations of the dipoles in the original system

and $\mathbf{P}_{3j-2}$ indicates the $(3j-2)^{th}$element in the vector **P.**

The change in polarizability tensor along Y and Z directions can be introduced similarly with varied indexes, and solutions for the system with the change of polarizabilities in multiple dipoles can be obtained by applying the same procedure as above iteratively.

By adding a rank-one matrix and calculating the new inverse matrix and dipole solution according to the procedure described above, we can solve the system where the property of either a single dipole or a set of dipoles are changed. Here, we only need to calculate the initial inverse matrix and the rest is only matrix multiplication, which is less computationally expensive compared to solving a linear system and can be accelerated using GPU/multiple CPUs.

Furthermore, the approach described above not only can find a new solution to the system when the polarizability of dipole $\mathbf{P}_j$ is changed, but also can be used as a valid approximation to calculate the new solution of the system when the addition or removal of dipoles happens, which will be discussed later.

1.3. Formulating new solutions when a dipole is added

1.3.1. Problem

The growth of a nanostructure can be represented by the addition of new dipoles to the system. Here, the relationship between the new solution to the polarizations and the original solution is derived using the rank-one decomposition when multiple dipoles are added to the system. Again, it is assumed the original linear system was solved to obtain the corresponding initial $\mathbf{A}^{-1}$ and $\mathbf{P}$. When a new dipole is added, the new inverse matrix ($\mathbf{A'}^{-1}$) can be calculated from them and used to evaluate the new polarizations of the dipoles ($\mathbf{P}'$).

First, when a dipole is added to the system, the corresponding matrix for the linear coefficients in the new system was denoted as $\mathbf{A}'$ and can be written as:

$$\mathbf{A}' = \begin{bmatrix} & \mathbf{A} & & \begin{matrix} (\mathbf{A'}_{N+1,1})^{\mathbf{T}} \\ (\mathbf{A'}_{N+1,2})^{\mathbf{T}} \\ \vdots \end{matrix} \\ \mathbf{A'}_{N+1,1} & \mathbf{A'}_{N+1,2} & \cdots & \boldsymbol{\alpha}_{N+1}{}^{-\mathbf{1}} \end{bmatrix} \tag{23}$$

where $\boldsymbol{\alpha}_{\boldsymbol{N+1}}$ is the polarizability tensor of the newly added dipole and $\mathbf{A}$ is the $3N \times 3N$ matrix which is constructed with the original N dipoles. $\mathbf{A'}_{N+1,k}$ is the 3×3 matrix that describes the dipole interaction between the newly added $(N+1)^{th}$ dipole and the kth dipole (see the general description of DDA and Eq. (7)). Note here the property that both $\mathbf{A}$ and $\mathbf{A}'$ are symmetric are used.

Eq. (23) can be expanded more explicitly with the new elements together with the original **A** matrix

as following:

$$\mathbf{A}' = \left[\begin{array}{ccccccc|ccc} & & & & & & & (\mathbf{A}'_{N+1,1})_{1,1} & (\mathbf{A}'_{N+1,1})_{2,1} & (\mathbf{A}'_{N+1,1})_{3,1} \\ & & & & & & & (\mathbf{A}'_{N+1,1})_{1,2} & (\mathbf{A}'_{N+1,1})_{2,2} & (\mathbf{A}'_{N+1,1})_{3,2} \\ & & & & & & & (\mathbf{A}'_{N+1,1})_{1,3} & (\mathbf{A}'_{N+1,1})_{2,3} & (\mathbf{A}'_{N+1,1})_{3,3} \\ & & & \mathbf{A} & & & & (\mathbf{A}'_{N+1,2})_{1,1} & (\mathbf{A}'_{N+1,2})_{2,1} & (\mathbf{A}'_{N+1,2})_{3,1} \\ & & & & & & & (\mathbf{A}'_{N+1,2})_{1,2} & (\mathbf{A}'_{N+1,2})_{2,2} & (\mathbf{A}'_{N+1,2})_{3,2} \\ & & & & & & & (\mathbf{A}'_{N+1,2})_{1,3} & (\mathbf{A}'_{N+1,2})_{2,3} & (\mathbf{A}'_{N+1,2})_{3,3} \\ & & & & & & & & \vdots & \\ (\mathbf{A}'_{N+1,1})_{1,1} & (\mathbf{A}'_{N+1,1})_{1,2} & (\mathbf{A}'_{N+1,1})_{1,3} & (\mathbf{A}'_{N+1,2})_{1,1} & (\mathbf{A}'_{N+1,2})_{1,2} & (\mathbf{A}'_{N+1,2})_{1,3} & & \alpha_{N+1}{}^{-1} & 0 & 0 \\ (\mathbf{A}'_{N+1,1})_{2,1} & (\mathbf{A}'_{N+1,1})_{2,2} & (\mathbf{A}'_{N+1,1})_{2,3} & (\mathbf{A}'_{N+1,2})_{2,1} & (\mathbf{A}'_{N+1,2})_{2,2} & (\mathbf{A}'_{N+1,2})_{2,3} & \cdots & 0 & \alpha_{N+1}{}^{-1} & 0 \\ (\mathbf{A}'_{N+1,1})_{3,1} & (\mathbf{A}'_{N+1,1})_{3,2} & (\mathbf{A}'_{N+1,1})_{3,3} & (\mathbf{A}'_{N+1,2})_{3,1} & (\mathbf{A}'_{N+1,2})_{3,2} & (\mathbf{A}'_{N+1,2})_{3,3} & & 0 & 0 & \alpha_{N+1}{}^{-1} \end{array}\right] \quad (24)$$

The corresponding vector for the incident beam with the newly added dipole can also be written as:

$$\mathbf{E}' = \begin{bmatrix} \mathbf{E} \\ \mathrm{E}_{N+1,X} \\ \mathrm{E}_{N+1,Y} \\ \mathrm{E}_{N+1,Z} \end{bmatrix} \quad (25)$$

where $\mathbf{E}$ denotes the 3N vector describing the incident beam before adding the new dipole, and $\mathrm{E}_{N+1,X}$, $\mathrm{E}_{N+1,Y}$and $\mathrm{E}_{N+1,Z}$ denotes the incident electric field distribution along the X, Y and Z axis at the $(N+1)^{th}$ dipole's position, respectively.

The influence of the new dipole on the original system is added sequentially for its polarizability in the X, Y and Z directions. The procedure to evaluate the solution of the new system after adding a dipole goes as follows:

1. A $(3N+1)\times(3N+1)$ matrix $\mathbf{A'}_X$ is defined, which takes the top-left $(3N+1)\times(3N+1)$ block of $\mathbf{A}'$, and its inverse matrix $\left(\mathbf{A'}_X{}^{-\mathbf{1}}\right)$ will be evaluated based on $\mathbf{A}^{-\mathbf{1}}$.

2. Once $\mathbf{A'}_X{}^{-\mathbf{1}}$ is solved, another matrix of $\mathbf{A'}_{X,Y}$ which takes the $(3N+2)\times(3N+2)$ top-left block of $\mathbf{A}'$ will be defined. Its inverse matrix $\left(\mathbf{A'}_{X,Y}{}^{-\mathbf{1}}\right)$ will be evaluated based on $\mathbf{A'}_X{}^{-\mathbf{1}}$.

3. Similar to step 2, once $\mathbf{A'}_{X,Y}{}^{-\mathbf{1}}$ is solved, the inverse of $\mathbf{A}'$ will be evaluated based on it.

This procedure expands the original matrix $\mathbf{A}$ to $\mathbf{A}'$ by adding a single row and column every time and calculating their corresponding inverse matrices through matrix rank-one decomposition. The corresponding $\mathbf{A'}_X$, $\mathbf{A'}_{X,Y}$, and $\mathbf{A}'$ are defined below:

$$
\mathbf{A'}_X = \begin{bmatrix} & & & & & & & (\mathbf{A'}_{N+1,1})_{1,1} \\ & & & & & & & (\mathbf{A'}_{N+1,1})_{1,2} \\ & & & & & & & (\mathbf{A'}_{N+1,1})_{1,3} \\ & & & \mathbf{A} & & & & (\mathbf{A'}_{N+1,2})_{1,1} \\ & & & & & & & (\mathbf{A'}_{N+1,2})_{1,2} \\ & & & & & & & (\mathbf{A'}_{N+1,2})_{1,3} \\ & & & & & & & \vdots \\ (\mathbf{A'}_{N+1,1})_{1,1} & (\mathbf{A'}_{N+1,1})_{1,2} & (\mathbf{A'}_{N+1,1})_{1,3} & (\mathbf{A'}_{N+1,2})_{1,1} & (\mathbf{A'}_{N+1,2})_{1,2} & (\mathbf{A'}_{N+1,2})_{1,3} & \cdots & {\alpha_{N+1}}^{-1} \end{bmatrix} \tag{26}
$$

$$
\mathbf{A'}_{X,Y} = \begin{bmatrix} & & & & & & & (\mathbf{A'}_{N+1,1})_{2,1} \\ & & & & & & & (\mathbf{A'}_{N+1,1})_{2,2} \\ & & & & & & & (\mathbf{A'}_{N+1,1})_{2,3} \\ & & & \mathbf{A'}_X & & & & (\mathbf{A'}_{N+1,2})_{2,1} \\ & & & & & & & (\mathbf{A'}_{N+1,2})_{2,2} \\ & & & & & & & (\mathbf{A'}_{N+1,2})_{2,3} \\ & & & & & & & \vdots \\ (\mathbf{A'}_{N+1,1})_{2,1} & (\mathbf{A'}_{N+1,1})_{2,2} & (\mathbf{A'}_{N+1,1})_{2,3} & (\mathbf{A'}_{N+1,2})_{2,1} & (\mathbf{A'}_{N+1,2})_{2,2} & (\mathbf{A'}_{N+1,2})_{2,3} & \cdots & {\alpha_{N+1}}^{-1} \end{bmatrix} \tag{27}
$$

$$
\mathbf{A'} = \begin{bmatrix} & & & & & & & (\mathbf{A'}_{N+1,1})_{3,1} \\ & & & & & & & (\mathbf{A'}_{N+1,1})_{3,2} \\ & & & & & & & (\mathbf{A'}_{N+1,1})_{3,3} \\ & & & \mathbf{A'}_{X,Y} & & & & (\mathbf{A'}_{N+1,2})_{3,1} \\ & & & & & & & (\mathbf{A'}_{N+1,2})_{3,2} \\ & & & & & & & (\mathbf{A'}_{N+1,2})_{3,3} \\ & & & & & & & \vdots \\ (\mathbf{A'}_{N+1,1})_{3,1} & (\mathbf{A'}_{N+1,1})_{3,2} & (\mathbf{A'}_{N+1,1})_{3,3} & (\mathbf{A'}_{N+1,2})_{3,1} & (\mathbf{A'}_{N+1,2})_{3,2} & (\mathbf{A'}_{N+1,2})_{3,3} & \cdots & {\alpha_{N+1}}^{-1} \end{bmatrix} \tag{28}
$$

By adding an extra column and row to the original matrix, the relation between the original inverse matrix and the current inverse matrix will be derived below. Once the relation is derived, it can be applied repeatedly to sequentially solve ${\mathbf{A'}_X}^{-\mathbf{1}}$, ${\mathbf{A'}_{X,Y}}^{-\mathbf{1}}$, and $\mathbf{A'}^{-\mathbf{1}}$ starting from $\mathbf{A}^{-\mathbf{1}}$.

1.3.2. Method

We will begin to derive ${\mathbf{A'}_X}^{-\mathbf{1}}$ based on $\mathbf{A}^{-\mathbf{1}}$, but it should be noted the same procedure can be used to calculate the rest inverse matrices (${\mathbf{A'}_{X,Y}}^{-\mathbf{1}}$ and $\mathbf{A'}^{-\mathbf{1}}$). $\mathbf{A'}_X$ is defined in Eq. (26), which can be decomposed into the summation of $\mathbf{A'}_{X,aux}$ and two rank-one matrices defined below:

$$\mathbf{A'}_{X,aux} = \begin{bmatrix} & & & & & & & 0 \\ & & & & & & & 0 \\ & & & & & & & 0 \\ & & & \mathbf{A} & & & & 0 \\ & & & & & & & 0 \\ & & & & & & & 0 \\ & & & & & & & \vdots \\ 0 & 0 & 0 & 0 & 0 & 0 & \dots & {\alpha_{N+1}}^{-1} \end{bmatrix} \tag{29}$$

where its last row and column are filled with 0s except the diagonal one. Its inverse matrix can be written explicitly based on $\mathbf{A^{-1}}$:

$$\mathbf{A'}_{X,aux}{}^{-1} = \begin{bmatrix} & & & & & & & 0 \\ & & & & & & & 0 \\ & & & & & & & 0 \\ & & & \mathbf{A^{-1}} & & & & 0 \\ & & & & & & & 0 \\ & & & & & & & 0 \\ & & & & & & & \vdots \\ 0 & 0 & 0 & 0 & 0 & 0 & \dots & \alpha_{N+1} \end{bmatrix} \tag{30}$$

The corresponding solution of dipoles, in this case, can also be written as:

$$\mathbf{P'}_{X,aux} = \begin{bmatrix} \mathbf{P} \\ \alpha_{N+1}\mathrm{E}_{N+1,X} \end{bmatrix} \tag{31}$$

With this auxiliary matrix, $\mathbf{A'}_X$ can be decomposed to the auxiliary matrix and two rank-one matrices:

$$\mathbf{A'}_X = \mathbf{A'}_{X,aux} + \begin{bmatrix} & & & \mathbf{0}_{(3N\times3N)} & & & & \mathbf{0}_{(3N\times1)} \\ \left(\mathbf{A'}_{N+1,1}\right)_{1,1} & \left(\mathbf{A'}_{N+1,1}\right)_{1,2} & \left(\mathbf{A'}_{N+1,1}\right)_{1,3} & \left(\mathbf{A'}_{N+1,2}\right)_{1,1} & \left(\mathbf{A'}_{N+1,2}\right)_{1,2} & \left(\mathbf{A'}_{N+1,2}\right)_{1,3} & \dots & 0 \end{bmatrix} + \begin{bmatrix} & \left(\mathbf{A'}_{N+1,1}\right)_{1,1} \\ & \left(\mathbf{A'}_{N+1,1}\right)_{1,2} \\ & \left(\mathbf{A'}_{N+1,1}\right)_{1,3} \\ \mathbf{0}_{(3N\times3N)} & \left(\mathbf{A'}_{N+1,2}\right)_{1,1} \\ & \left(\mathbf{A'}_{N+1,2}\right)_{1,2} \\ & \left(\mathbf{A'}_{N+1,2}\right)_{1,3} \\ & \vdots \\ \mathbf{0}_{(1\times3N)} & 0 \end{bmatrix} \tag{32}$$

where $\mathbf{0}_{(i\times j)}$ indicates a $i \times j$ block matrix composed of only 0s.

For simplicity and generality, we denote the extra row and column except for the last element as **B,** so that:

$$\mathbf{A'}_X = \mathbf{A'}_{X,aux} + \begin{bmatrix} \mathbf{0}_{(3N\times 3N)} & \mathbf{0}_{(3N\times 1)} \\ \mathbf{B} & 0 \end{bmatrix} + \begin{bmatrix} \mathbf{0}_{(3N\times 3N)} & \mathbf{B}^{\mathbf{T}} \\ \mathbf{0}_{(1\times 3N)} & 0 \end{bmatrix} \tag{33}$$

Then after applying **Lemma 1** twice, we will be able to evaluate ${\mathbf{A'}_X}^{-\mathbf{1}}$ based on $\mathbf{A'}_{X,aux}$, which can be explicitly expressed by $\mathbf{A}^{-\mathbf{1}}$ (Eq. (30)) below.

First, the inverse matrix of $\mathbf{A'}_{X,inter} = \mathbf{A'}_{X,aux} + \begin{bmatrix} \mathbf{0}_{(3N\times 3N)} & \mathbf{0}_{(3N\times 1)} \\ \mathbf{B} & 0 \end{bmatrix}$ is calculated:

$${\mathbf{A'}_{X,inter}}^{-\mathbf{1}} = \begin{bmatrix} & & & & 0 \\ & & & & 0 \\ & & & & 0 \\ & & \mathbf{A}^{-\mathbf{1}} & & 0 \\ & & & & 0 \\ & & & & 0 \\ & & & & \cdots \\ -\alpha_{N+1}\mathrm{C}_1 & -\alpha_{N+1}\mathrm{C}_2 & \cdots\ \cdots & -\alpha_{N+1}\mathrm{C}_\mathrm{N} & \alpha_{N+1} \end{bmatrix} \tag{34}$$

where $\mathrm{C_k}$ is the kth element of vector $\mathbf{C}$, which is defined as below:

$$\mathbf{C} = \mathbf{A}^{-\mathbf{1}}\mathbf{B}^{\mathbf{T}} = (\mathbf{A}^{-\mathbf{1}})^{\mathbf{T}}\mathbf{B}^{\mathbf{T}} \tag{35}$$

Note we use the property that $\mathbf{A}^{-\mathbf{1}}$ is symmetric so that $\mathbf{A}^{-\mathbf{1}} = (\mathbf{A}^{-\mathbf{1}})^{\mathbf{T}}$.

The solution of the dipoles ($\mathbf{P'}_{X,inter}$) corresponding to $\mathbf{A'}_{X,inter}$ can be calculated via:

$$\mathbf{P'}_{X,inter} = \mathbf{P'}_{X,aux} - \alpha_{N+1}\begin{bmatrix} 0 \\ 0 \\ 0 \\ \vdots \\ \mathbf{C}^{\mathbf{T}}\cdot\mathbf{E} \end{bmatrix} = \mathbf{P'}_{X,aux} - \alpha_{N+1}\begin{bmatrix} 0 \\ 0 \\ 0 \\ \vdots \\ (\mathbf{B}\mathbf{A}^{-\mathbf{1}})\cdot\mathbf{E} \end{bmatrix} = \mathbf{P'}_{X,aux} - \alpha_{N+1}\begin{bmatrix} 0 \\ 0 \\ 0 \\ \vdots \\ \mathbf{B}(\mathbf{A}^{-\mathbf{1}}\mathbf{E}) \end{bmatrix}$$
$$= \mathbf{P'}_{X,aux} - \alpha_{N+1}\begin{bmatrix} 0 \\ 0 \\ 0 \\ \cdots \\ \mathbf{B}\cdot\mathbf{P} \end{bmatrix} = \begin{bmatrix} \mathbf{P} \\ \alpha_{N+1}\mathrm{E}_{N+1,X} - \alpha_{N+1}\mathbf{B}\cdot\mathbf{P} \end{bmatrix} \tag{36}$$

Then, the inverse matrix of $\mathbf{A'}_X = \mathbf{A'}_{X,inter} + \begin{bmatrix} \mathbf{0}_{(3N\times 3N)} & \mathbf{B}^{\mathbf{T}} \\ \mathbf{0}_{(1\times 3N)} & 0 \end{bmatrix}$ and its corresponding dipole distribution $\mathbf{P'}_X$ can be evaluated by **Lemma 1** as:

$${\mathbf{A'}_X}^{-\mathbf{1}} = {\mathbf{A'}_{X,inter}}^{-\mathbf{1}} + \frac{\alpha_{N+1}}{1-\alpha_{N+1}\mathrm{D}}\begin{bmatrix} \mathbf{C} \\ -\alpha_{N+1}\mathrm{D} \end{bmatrix}\begin{bmatrix} \mathbf{C}^T & -1 \end{bmatrix} \tag{37}$$

$$\mathbf{P'}_X = \mathbf{P'}_{X,inter} - \frac{1}{1-\alpha_{N+1}\mathrm{D}}\left(\alpha_{N+1}\mathrm{E}_{N+1,X} - \alpha_{N+1}\mathbf{B}\cdot\mathbf{P}\right)\begin{bmatrix}\mathbf{C}\\ -\alpha_{N+1}\mathrm{D}\end{bmatrix} \quad (38)$$

where D is defined as $\mathrm{D} = \mathbf{B}\cdot\mathbf{C}$ and $\alpha_{N+1}\mathrm{E}_{N+1,X} - \alpha_{N+1}\mathbf{B}\cdot\mathbf{P}$ is the last element of $\mathbf{P'}_{X,inter}$.

Thus, the inverse matrix and its corresponding dipole distribution of $\mathbf{A'}_X$, which has an extra column and row compared to $\mathbf{A}$, can be solved. The relation defined by Eq. (34)-(38) is general for any symmetric matrices of $\mathbf{A}$ and $\mathbf{A'}_X$, where $\mathbf{A'}_X$ has an extra column and row compared to $\mathbf{A}$.

By applying this relation three times, the inverse matrix of $\mathbf{A'}$ and its corresponding dipole distribution $\mathbf{P'}$ can be obtained. In the case of adding more dipoles, the linear system can be solved by applying the relation multiple times iteratively.

1.4. Formulating new solutions when a dipole is removed

1.4.1. Problem

In the previous section, we formulate the change in the polarizations when dipoles are added to the system, which represents nanostructure growth. Similarly, with the etching of the nanostructure, the equivalent process can be represented by removing the old dipoles from the system. So, through the matrix rank-one decomposition, the relationship between the new solution of polarizations and the original solution in an etching process can be derived. Assuming the original linear system including the old dipoles is solved to obtain the corresponding $\mathbf{A}^{-1}$ and $\mathbf{P}$. Here we assume there is $(N+1)$ dipoles in the original system, with one dipole to be removed. When one dipole is removed, the new inverse matrix ($\mathbf{A'}^{-1}$) can be calculated from $\mathbf{A}^{-1}$ and used to evaluate the new solution of dipoles ($\mathbf{P'}$). Thus, the size of $\mathbf{A}$ is $3(N+1)\times 3(N+1)$, while the size of $\mathbf{A'}$ is $3N \times 3N$.

Again, when a dipole is removed from the system, the matrix for the linear coefficients was denoted as $\mathbf{A'}$ and can be written as a block of the original $\mathbf{A}$ matrix:

$$\mathbf{A} = \begin{bmatrix} & \mathbf{A'} & & (\mathbf{A}_{N+1,1})^{\mathbf{T}} \\ & & & (\mathbf{A}_{N+1,2})^{\mathbf{T}} \\ & & & \vdots \\ \mathbf{A}_{N+1,1} & \mathbf{A}_{N+1,2} & \cdots & \boldsymbol{\alpha}_{N+1}{}^{-1} \end{bmatrix} \quad (39)$$

We can also write it explicitly with the elements:

$$
\mathbf{A}=\begin{bmatrix}
\mathbf{A}' & \begin{matrix}
(\mathbf{A}_{N+1,1})_{1,1} & (\mathbf{A}_{N+1,1})_{2,1} & (\mathbf{A}_{N+1,1})_{3,1}\\
(\mathbf{A}_{N+1,1})_{1,2} & (\mathbf{A}_{N+1,1})_{2,2} & (\mathbf{A}_{N+1,1})_{3,2}\\
(\mathbf{A}_{N+1,1})_{1,3} & (\mathbf{A}_{N+1,1})_{2,3} & (\mathbf{A}_{N+1,1})_{3,3}\\
(\mathbf{A}_{N+1,2})_{1,1} & (\mathbf{A}_{N+1,2})_{2,1} & (\mathbf{A}_{N+1,2})_{3,1}\\
(\mathbf{A}_{N+1,2})_{1,2} & (\mathbf{A}_{N+1,2})_{2,2} & (\mathbf{A}_{N+1,2})_{3,2}\\
(\mathbf{A}_{N+1,2})_{1,3} & (\mathbf{A}_{N+1,2})_{2,3} & (\mathbf{A}_{N+1,2})_{3,3}\\
 & \vdots &
\end{matrix}\\
\begin{matrix}
(\mathbf{A}_{N+1,1})_{1,1} & (\mathbf{A}_{N+1,1})_{1,2} & (\mathbf{A}_{N+1,1})_{1,3} & (\mathbf{A}_{N+1,2})_{1,1} & (\mathbf{A}_{N+1,2})_{1,2} & (\mathbf{A}_{N+1,2})_{1,3} & \\
(\mathbf{A}_{N+1,1})_{2,1} & (\mathbf{A}_{N+1,1})_{2,2} & (\mathbf{A}_{N+1,1})_{2,3} & (\mathbf{A}_{N+1,2})_{2,1} & (\mathbf{A}_{N+1,2})_{2,2} & (\mathbf{A}_{N+1,2})_{2,3} & \cdots\\
(\mathbf{A}_{N+1,1})_{3,1} & (\mathbf{A}_{N+1,1})_{3,2} & (\mathbf{A}_{N+1,1})_{3,3} & (\mathbf{A}_{N+1,2})_{3,1} & (\mathbf{A}_{N+1,2})_{3,2} & (\mathbf{A}_{N+1,2})_{3,3} &
\end{matrix} & \begin{matrix}
{\alpha_{N+1}}^{-1} & 0 & 0\\
0 & {\alpha_{N+1}}^{-1} & 0\\
0 & 0 & {\alpha_{N+1}}^{-1}
\end{matrix}
\end{bmatrix} \tag{40}
$$

The original vector for the incident beam containing the removed dipole can be written as:

$$
\mathbf{E}=\begin{bmatrix}\mathbf{E}'\\ \mathrm{E}_{N+1,X}\\ \mathrm{E}_{N+1,Y}\\ \mathrm{E}_{N+1,Z}\end{bmatrix} \tag{41}
$$

The procedure to evaluate the solution of the new system after removing a dipole is similar to the one discussed in the last section by getting rid of the effect of the removed dipole's polarizability in X, Y, and Z directions, and goes as follows:

1. We define a $(3N+2)\times(3N+2)$ matrix $\mathbf{A'}_Z$, which takes the top-left $(3N+2)\times(3N+2)$ block of $\mathbf{A}$, and its inverse matrix ($\mathbf{A'}_Z{}^{\mathbf{-1}}$) will be evaluated based on $\mathbf{A^{-1}}$.

2. Once $\mathbf{A'}_Z{}^{\mathbf{-1}}$ is solved, another matrix of $\mathbf{A'}_{Z,Y}$ which takes the $(3N+1)\times(3N+1)$ top-left block $\mathbf{A}$ will be defined. Its inverse matrix ($\mathbf{A'}_{Z,Y}{}^{\mathbf{-1}}$) will be evaluated based on $\mathbf{A'}_Z{}^{\mathbf{-1}}$.

3. Similar to step 2, once $\mathbf{A'}_{Z,Y}{}^{\mathbf{-1}}$ is solved, the inverse of $\mathbf{A'}$ will be evaluated based on it.

This procedure shrinks the original matrix $\mathbf{A}$ to $\mathbf{A'}$ by deleting a single row and column every time and calculating their corresponding inverse matrices through matrix rank-one decomposition. The corresponding $\mathbf{A'}_Z$, $\mathbf{A'}_{Y,Z}$, and $\mathbf{A'}$ are defined below:

$$
\mathbf{A}=\begin{bmatrix}
\mathbf{A}'_Z & \begin{matrix}(\mathbf{A}_{N+1,1})_{3,1}\\ (\mathbf{A}_{N+1,1})_{3,2}\\ (\mathbf{A}_{N+1,1})_{3,3}\\ (\mathbf{A}_{N+1,2})_{3,1}\\ (\mathbf{A}_{N+1,2})_{3,2}\\ (\mathbf{A}_{N+1,2})_{3,3}\\ \vdots\end{matrix}\\
\begin{matrix}(\mathbf{A}_{N+1,1})_{3,1} & (\mathbf{A}_{N+1,1})_{3,2} & (\mathbf{A}_{N+1,1})_{3,3} & (\mathbf{A}_{N+1,2})_{3,1} & (\mathbf{A}_{N+1,2})_{3,2} & (\mathbf{A}_{N+1,2})_{3,3} & \cdots\end{matrix} & {\alpha_{N+1}}^{-1}
\end{bmatrix} \tag{42}
$$

$$\mathbf{A}'_Z = \begin{bmatrix} & & & & & & & (\mathbf{A}_{N+1,1})_{2,1} \\ & & & & & & & (\mathbf{A}_{N+1,1})_{2,2} \\ & & & & & & & (\mathbf{A}_{N+1,1})_{2,3} \\ & & & \mathbf{A}'_{Z,Y} & & & & (\mathbf{A}_{N+1,2})_{2,1} \\ & & & & & & & (\mathbf{A}_{N+1,2})_{2,2} \\ & & & & & & & (\mathbf{A}_{N+1,2})_{2,3} \\ & & & & & & & \vdots \\ (\mathbf{A}_{N+1,1})_{2,1} & (\mathbf{A}_{N+1,1})_{2,2} & (\mathbf{A}_{N+1,1})_{2,3} & (\mathbf{A}_{N+1,2})_{2,1} & (\mathbf{A}_{N+1,2})_{2,2} & (\mathbf{A}_{N+1,2})_{2,3} & \cdots & \alpha_{N+1}{}^{-1} \end{bmatrix} \tag{43}$$

$$\mathbf{A}'_{Z,Y} = \begin{bmatrix} & & & & & & & (\mathbf{A}_{N+1,1})_{1,1} \\ & & & & & & & (\mathbf{A}_{N+1,1})_{1,2} \\ & & & & & & & (\mathbf{A}_{N+1,1})_{1,3} \\ & & & \mathbf{A}' & & & & (\mathbf{A}_{N+1,2})_{1,1} \\ & & & & & & & (\mathbf{A}_{N+1,2})_{1,2} \\ & & & & & & & (\mathbf{A}_{N+1,2})_{1,3} \\ & & & & & & & \vdots \\ (\mathbf{A}_{N+1,1})_{1,1} & (\mathbf{A}_{N+1,1})_{1,2} & (\mathbf{A}_{N+1,1})_{1,3} & (\mathbf{A}_{N+1,2})_{1,1} & (\mathbf{A}_{N+1,2})_{1,2} & (\mathbf{A}_{N+1,2})_{1,3} & \cdots & \alpha_{N+1}{}^{-1} \end{bmatrix} \tag{44}$$

By removing a column and row from the original matrix, the current inverse matrix can be expressed as a function of the original inverse matrix and will be derived. Once the relation is derived, we apply it repeatedly to sequentially find $\mathbf{A}'_Z{}^{-\mathbf{1}}$, $\mathbf{A}'_{Z,Y}{}^{-\mathbf{1}}$, and $\mathbf{A}'^{-\mathbf{1}}$ starting from $\mathbf{A}^{-\mathbf{1}}$.

1.4.2. Method

We start to derive $\mathbf{A}'_Z{}^{-\mathbf{1}}$ based on $\mathbf{A}^{-\mathbf{1}}$. The relation between $\mathbf{A}'_Z$ and $\mathbf{A}$ is shown in Eq. (42), where $\mathbf{A}'_Z$ is a $(3N+2)\times(3N+2)$ matrix. The inverse matrix $\mathbf{A}'_Z{}^{-\mathbf{1}}$ will be calculated through an auxiliary matrix, which does not consider the interaction between the $(N+1)^{th}$ dipole and the rest of the dipoles:

$$\mathbf{A}'_{Z,aux} = \begin{bmatrix} & & & & & & & 0 \\ & & & & & & & 0 \\ & & & & & & & 0 \\ & & & \mathbf{A}'_Z & & & & 0 \\ & & & & & & & 0 \\ & & & & & & & 0 \\ & & & & & & & \vdots \\ 0 & 0 & 0 & 0 & 0 & 0 & \cdots & \alpha_{N+1}{}^{-1} \end{bmatrix} \tag{45}$$

Its inverse matrix can be evaluated directly as:

$$\mathbf{A'}_{Z,aux}{}^{\mathbf{-1}} = \begin{bmatrix} & & & \mathbf{A'}_{Z}{}^{\mathbf{-1}} & & & & \begin{matrix}0\\0\\0\\0\\0\\0\\\vdots\end{matrix} \\ 0 & 0 & 0 & 0 & 0 & 0 & \dots & \alpha_{N+1} \end{bmatrix}$$

(46)

Thus, once $\mathbf{A'}_{Z,aux}{}^{\mathbf{-1}}$ is calculated, simply taking the top-left $(3N+2) \times (3N+2)$ block matrix will give $\mathbf{A'}_{Z}{}^{\mathbf{-1}}$.

$\mathbf{A'}_{Z,aux}$ can be expressed as the summation of **A** and two rank-one matrices, so that:

$$\mathbf{A'}_{Z,aux} = \mathbf{A} + \begin{bmatrix} \mathbf{0}_{(3(N+1)\times 3(N+1))} & \mathbf{B^T} \\ \mathbf{0}_{(1\times 3(N+1))} & 0 \end{bmatrix} + \begin{bmatrix} \mathbf{0}_{(3(N+1)\times 3(N+1))} & \mathbf{0}_{(3(N+1)\times 1)} \\ \mathbf{B} & 0 \end{bmatrix} \tag{47}$$

where **B** is the negative of the last row without the last element in **A**. Following the same procedure described in the last section, the $\mathbf{A'}_{Z,aux}$ can be evaluated as follows:

First an intermediate matrix ($\mathbf{A'}_{Z,inter}$) is defined as:

$$\mathbf{A'}_{Z,inter} = \mathbf{A} + \begin{bmatrix} \mathbf{0}_{(3(N+1)\times 3(N+1))} & \mathbf{B^T} \\ \mathbf{0}_{(1\times 3(N+1))} & 0 \end{bmatrix}$$

$$= \begin{bmatrix} & & & \mathbf{A}'_{Z} & & & & \begin{matrix}0\\0\\0\\0\\0\\0\\\vdots\end{matrix} \\ (\mathbf{A}_{N+1,1})_{3,1} & (\mathbf{A}_{N+1,1})_{3,2} & (\mathbf{A}_{N+1,1})_{3,3} & (\mathbf{A}_{N+1,2})_{3,1} & (\mathbf{A}_{N+1,2})_{3,2} & (\mathbf{A}_{N+1,2})_{3,3} & \dots & \alpha_{N+1}{}^{-1} \end{bmatrix}$$

(48)

As indicated by Eq. (34), the inverse matrix of $\mathbf{A'}_{Z,inter}$ has the form as follows:

$$\mathbf{A'}_{Z,inter}{}^{\mathbf{-1}} = \begin{bmatrix} & & \mathbf{A'}_{Z}{}^{\mathbf{-1}} & & & \begin{matrix}0\\0\\0\\0\\0\\0\\\vdots\end{matrix} \\ \mathrm{X}_1 & \mathrm{X}_2 & \mathrm{X}_3 & \dots & \mathrm{X}_{3N+2} & \alpha_{N+1} \end{bmatrix}$$

(49)

where X_i is unknown constants that will be solved but not be used further. So, we only need to

calculate ${\mathbf{A}'_{Z,inter}}^{-1}$ and take the top-left $(3N+2)\times(3N+2)$ block to get ${\mathbf{A}'_{Z}}^{-1}$, instead of actually calculating ${\mathbf{A}'_{Z,aux}}^{-1}$ further by adding a second rank-one matrix.

Again, ${\mathbf{A}'_{Z,inter}}^{-1}$ can be evaluated via **Lemma 1**:

$$ {\mathbf{A}'_{Z,inter}}^{-1} = \mathbf{A}^{-1} - \frac{1}{1+\mathrm{D}}\mathbf{C}(\mathbf{A}^{-1})_{-1} \tag{50} $$

where $(\mathbf{A}^{-1})_{-1}$indicates the last row of $\mathbf{A}^{-1}$, D is the trace of $\begin{bmatrix} \mathbf{0}_{(3(N+1)\times 3(N+1))} & \mathbf{B}^{\mathrm{T}} \\ \mathbf{0}_{(1\times 3(N+1))} & 0 \end{bmatrix}\mathbf{A}^{-1}$ and can be simply calculated via $\mathrm{D} = [\mathbf{B}, 0]\cdot((\mathbf{A}^{-1})_{-1})^{\mathrm{T}}$, and $\mathbf{C} = \mathbf{A}^{-1}\begin{bmatrix}\mathbf{B}^{\mathrm{T}} \\ 0\end{bmatrix}$.

By applying this transformation three times, the ${\mathbf{A}'_{Z}}^{-1}$, ${\mathbf{A}'_{Z,Y}}^{-1}$ and $\mathbf{A}^{-1}$ and the corresponding polarizations of dipoles **P** can be obtained.

As an example, the equivalence between the direct implementation of DDA and iterative rank-one decomposition accelerated DDA method (RD-DDA) has been shown for a limited number of dipoles, which is available at https://github.com/croningp/RD-DDA.

1.5. The validation and numerical approximation for replacement, growth, and etching

As described above, we can solve the exact solution when a replacement, addition or removal of dipoles happens through the rank-one decomposition method. However, considering the growth/etching process, the size of the coefficient matrix (A) will increase/decrease at each step, this makes the algorithmic implementation computationally less efficient. Instead, the approach described in Eq.(21)-(22) offers a computationally efficient method for replacement, growth and etching which will be discussed below.

First, by defining the outer bounds, all the possible positions where the dipoles can exist are considered to create the initial **A** matrix. The absence of the jth dipole in a specific position can be approximated by setting its polarizability as τ, where $\tau \approx 0$. During the growth process, when the nanostructure growth front reaches the position of the jth dipole, its polarizability can be changed from τ to α_j. Similarly, during the etching process, when the nanostructure etching front reaches the kth dipole happens, its polarizability can be changed from α_k to τ. In both cases, the system can be solved iteratively according to Eq. (21)-(22).

Here, for good numerical stability in estimating the polarizations of dipoles, the selection of τ is crucial. A series of τs in the case of the growth of Au cubes ($10\times10\times10$ nm^3) to Au nanorods ($10\times10\times30$ nm^3) in the water medium, with a dipole length of 1 nm, were benchmarked. The UV-Vis spectra from the exact solution were calculated from the method described in Section 1.3 as

standard data, where the initial and final dipole numbers were 1000 and 3000 respectively. The complex refractive index data for Au was used based on reference[6]. The calculated extinction efficiency factors (Q_{ext}) with layer wise growth (sequential dipole addition to complete one layer) are shown in **Figure S2**.

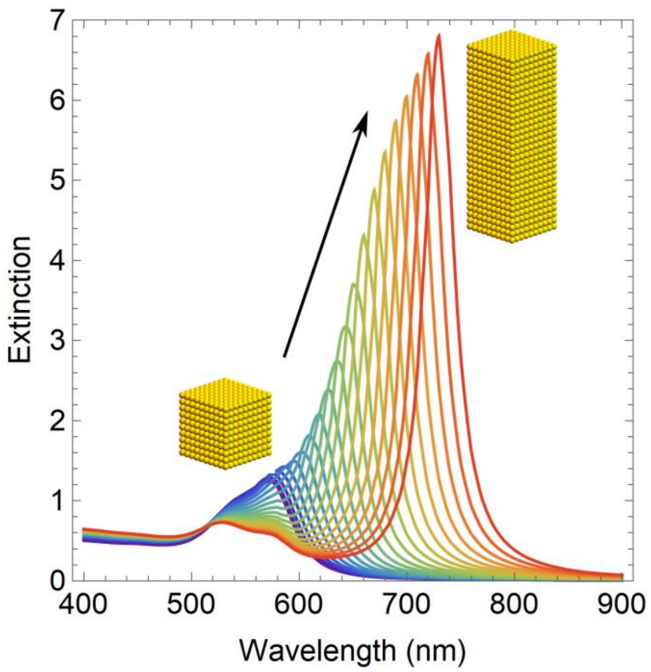

Figure S2. The simulated UV-Vis (extinction efficiency factors) in a growth trajectory. The initial geometry is a cube with a 10 nm length, and the final geometry is a rod with the size of $10 \times 10 \times 30$ nm^3. The growth happens by adding dipoles of 1 nm in length to the initial cube. The blue-to-red transition indicates the growth of the formation of nanorods. The results shown in the figure are sampled after adding a complete layer composed of 100 dipoles.

Then, the same well-defined growth trajectory can be simulated via two possible strategies:

Strategy 1: Select Au cube ($10 \times 10 \times 10$ nm^3) as the initial geometry with outer bounds defined by nanorod geometry ($10 \times 10 \times 30$ nm^3), and the UV-Vis spectra from the same growth trajectory were simulated using a small enough τ for the non-Au dipoles.

Strategy 2: Reverse the growth trajectory to create an "etching trajectory". The corresponding UV-Vis spectra from this etching trajectory with a small enough value of τ for non-Au dipoles can also be simulated. Reverse the sequence of the UV-Vis spectra will give the UV-Vis change in the growth process.

By changing the polarizability of the dipoles from τ to α, the UV-Vis spectra of such growth or etching trajectory can be created. Imaging there is one dipole composed of the medium, whose polarizabilities is $\tau \approx 0$, we can write **A** matrix as below:

$$\mathbf{A} = \begin{bmatrix} \alpha_1{}^{-1} & 0 & 0 & & & & & & \\ 0 & \alpha_1{}^{-1} & 0 & & \cdots & & & \cdots & \\ 0 & 0 & \alpha_1{}^{-1} & & & & & & \\ & & & \tau^{-1} & 0 & 0 & & & \\ & \vdots & & 0 & \tau^{-1} & 0 & & \vdots & \\ & & & 0 & 0 & \tau^{-1} & & & \\ & & & & & & \alpha_N{}^{-1} & 0 & 0 \\ & \cdots & & & \cdots & & 0 & \alpha_N{}^{-1} & 0 \\ & & & & & & 0 & 0 & \alpha_N{}^{-1} \end{bmatrix} \tag{51}$$

When the polarizability of the jth dipole is changed from τ to α, there would be a factor defined as $\frac{\alpha_j{}'^{-1}-\alpha_j{}^{-1}}{1+\left(\alpha_j{}'^{-1}-\alpha_j{}^{-1}\right)(\mathbf{A}^{-1})_{3j-2,3j-2}}$ in both Eq. (21) and (22), which can be calculated as:

$$\frac{\alpha_j{}'^{-1} - \alpha_j{}^{-1}}{1 + \left(\alpha_j{}'^{-1} - \alpha_j{}^{-1}\right)(\mathbf{A}^{-1})_{3j-2,3j-2}} = \frac{\alpha^{-1} - \tau^{-1}}{1 + (\alpha^{-1} - \tau^{-1})(\mathbf{A}^{-1})_{3j-2,3j-2}} \tag{52}$$

Given $\tau \to 0$, we should estimate the value of this term to check if it is divergent or convergent to evaluate the numerical stability. We need to estimate the value of $(\mathbf{A}^{-1})_{3j-2,3j-2}$, which is the $(3j-2)^{th}$diagonal element in $\mathbf{A}^{-1}$. It can be shown $(\mathbf{A}^{-1})_{3j-2,3j-2}$ can be approximated as τ when $\tau \to 0$ as follows:

First, a matrix labelled as $\mathbf{A}_s$ was constructed by sorting the existing dipoles so that the dipoles purely composed of Au are indexed lower and those of the medium are indexed higher. We assume there are N dipoles in total and M of them are composed of pure Au and $(N-M)$ composed of the medium, then the matrix $\mathbf{A}_s$ can be written as:

$$\mathbf{A}_s = \begin{bmatrix} \mathbf{A}_{1,1} & \dots & \mathbf{A}_{1,M} & \dots & \mathbf{A}_{1,N} \\ \vdots & \ddots & \vdots & \ddots & \vdots \\ \mathbf{A}_{M,1} & \dots & \mathbf{A}_{M,M} & \dots & \mathbf{A}_{M,N} \\ \vdots & \ddots & \vdots & \ddots & \vdots \\ \mathbf{A}_{N,1} & \dots & \mathbf{A}_{M,1} & \dots & \mathbf{A}_{N,N} \end{bmatrix} \tag{53}$$

where $\mathbf{A}_{i,j}$ is the 3×3 matrix that describes the interaction between the ith and j^{th} dipole as indicated in Eq. (7). We define the top-left $3M \times 3M$ block of $\mathbf{A}_s$ as $\mathbf{A}'$, which consists of $M \times M$ block matrices. The shape of these small block matrices is 3×3. $\mathbf{A}'$ is identical to an $\mathbf{A}$ matrix constructed from only considering Au dipoles listed in the same order.

$$\mathbf{A}_s = \begin{bmatrix} & & & \cdots & (\mathbf{A}_s)_{1,N} \\ & \mathbf{A}' & & \ddots & \vdots \\ & & & \cdots & (\mathbf{A}_s)_{M,N} \\ \vdots & \ddots & \vdots & \ddots & \vdots \\ (\mathbf{A}_s)_{N,1} & \cdots & (\mathbf{A}_s)_{M,1} & \cdots & (\mathbf{A}_s)_{N,N} \end{bmatrix} \tag{54}$$

For any matrix of $(\mathbf{A}_s)_{i,i}$ where $i > M$, the diagonal element is τ^{-1}, with τ approximating 0. Later, the inverse matrix of $\mathbf{A}_s$ can be estimated as follows:

1. We rewrite $\mathbf{A}_s$ as:

$$\mathbf{A}_s = \begin{bmatrix} \mathbf{A}' & \boldsymbol{B}^{\boldsymbol{T}} \\ \boldsymbol{B} & \mathbf{A}'' \end{bmatrix} \tag{55}$$

where $\mathbf{A}''$ is a $3(N-M) \times 3(N-M)$ matrix identical to an $\mathbf{A}$ matrix constructed from medium dipoles listed in the same order, whose diagonal elements are all equal to τ^{-1}. $\boldsymbol{B}$ and $\boldsymbol{B}^{\boldsymbol{T}}$ consider the interaction terms between the Au dipole set and the medium dipole set.

2. According to the inverse of the block matrix, $\mathbf{A}_s{}^{-1}$ can be written as:

$$\mathbf{A}_s{}^{-1} = \begin{bmatrix} \mathbf{A}' & \boldsymbol{B}^{\boldsymbol{T}} \\ \boldsymbol{B} & \mathbf{A}'' \end{bmatrix}^{-1} = \begin{bmatrix} \mathbf{A}'^{-1} + \mathbf{A}'^{-1}\boldsymbol{B}^{\boldsymbol{T}}\left(\mathbf{A}'' - \boldsymbol{B}\mathbf{A}'^{-1}\boldsymbol{B}^{\boldsymbol{T}}\right)^{-1}\boldsymbol{B}\mathbf{A}'^{-1} & -\mathbf{A}'^{-1}\boldsymbol{B}^{\boldsymbol{T}}\left(\mathbf{A}'' - \boldsymbol{B}\mathbf{A}'^{-1}\boldsymbol{B}^{\boldsymbol{T}}\right)^{-1} \\ -\left(\mathbf{A}'' - \boldsymbol{B}\mathbf{A}'^{-1}\boldsymbol{B}^{\boldsymbol{T}}\right)^{-1}\boldsymbol{B}\mathbf{A}'^{-1} & \left(\mathbf{A}'' - \boldsymbol{B}\mathbf{A}'^{-1}\boldsymbol{B}^{\boldsymbol{T}}\right)^{-1} \end{bmatrix} \tag{56}$$

here, we need to calculate the diagonal elements of this matrix. Since τ approximates 0, the off-diagonal elements of $\left(\mathbf{A}'' - \boldsymbol{B}\mathbf{A}'^{-1}\boldsymbol{B}^{\boldsymbol{T}}\right)$ are infinitely small compared to τ^{-1}, while the diagonal elements can be approximated by τ^{-1}. Thus, we can write:

$$\frac{\left(\mathbf{A}'' - \boldsymbol{B}\mathbf{A}'^{-1}\boldsymbol{B}^{\boldsymbol{T}}\right)}{\tau^{-1}} \approx \boldsymbol{I} \tag{57}$$

where $\boldsymbol{I}$ is the $3M \times 3M$ identity matrix. Based on it, we can have:

$$\left(\mathbf{A}'' - \boldsymbol{B}\mathbf{A}'^{-1}\boldsymbol{B}^{\boldsymbol{T}}\right)^{-1} \approx \tau\boldsymbol{I} \tag{58}$$

It indicates that any diagonal element that corresponds to the dipole composed of medium and whose index is larger than $3M$ in $\mathbf{A_s}^{-1}$, can be approximated by τ.

The original $\mathbf{A}$ matrix can be constructed by swapping the columns and rows of $\mathbf{A_s}$. During this transformation, the inverse matrix $\mathbf{A^{-1}}$ can also be constructed by swapping the corresponding rows and columns in $\mathbf{A_s}^{-1}$. If the jth dipole that constructed $\mathbf{A}$ matrix is composed of the medium, we have:

$$(\mathbf{A^{-1}})_{3j-2,3j-2} = (\mathbf{A^{-1}})_{3j-1,3j-1} = (\mathbf{A^{-1}})_{3j,3j} \approx \tau \tag{59}$$

Thus, $(\mathbf{A^{-1}})_{3j-2,3j-2}$ that correspond to a dipole composed of the medium will be τ, which indicates Eq. (52) can be approximated as:

$$\frac{\alpha^{-1} - \tau^{-1}}{1 + (\alpha^{-1} - \tau^{-1})(\mathbf{A^{-1}})_{3j-2,3j-2}} \approx \frac{\alpha^{-1} - \tau^{-1}}{1 + (\alpha^{-1} - \tau^{-1})\tau} = \tau^{-1} - \alpha\tau^{-2} \tag{60}$$

which approximates infinity intrinsically and gives a large numerical error when τ is too small and the numeric precision is not high enough. It should be noted that it is just an estimation of $\frac{\alpha^{-1}-\tau^{-1}}{1+(\alpha^{-1}-\tau^{-1})(\mathbf{A^{-1}})_{3j-2,3j-2}}$ when the polarizability of the jth dipole in the X direction is changed from τ to α. Later, it can be observed that $\tau^{-1} - \alpha\tau^{-2}$ is good to estimate the order of magnitude of $\frac{\alpha^{-1}-\tau^{-1}}{1+(\alpha^{-1}-\tau^{-1})(\mathbf{A^{-1}})_{3j-2,3j-2}}$ in the subsequent steps: their orders go up similarly when τ decreases. Additionally, the magnitude of $\frac{\alpha^{-1}-\tau^{-1}}{1+(\alpha^{-1}-\tau^{-1})(\mathbf{A^{-1}})_{3j-2,3j-2}}$ increases when smaller τ is used.

However, for strategy 2 where a dipole is removed, we have $\alpha_j = \alpha$ and $\alpha_j{}' = \tau$. The value of $(\mathbf{A^{-1}})_{3j-2,3j-2}$ is not as small as τ, which means $\frac{\alpha_j{}'^{-1}-\alpha_j{}^{-1}}{1+\left(\alpha_j{}'^{-1}-\alpha_j{}^{-1}\right)(\mathbf{A^{-1}})_{3j-2,3j-2}}$ can be approximated as:

$$\frac{\alpha_j{}'^{-1} - \alpha_j{}^{-1}}{1 + \left(\alpha_j{}'^{-1} - \alpha_j{}^{-1}\right)(\mathbf{A^{-1}})_{3j-2,3j-2}} = \frac{1}{\frac{1}{(\tau^{-1} - \alpha^{-1})} + (\mathbf{A^{-1}})_{3j-2,3j-2}} \approx \frac{1}{(\mathbf{A^{-1}})_{3j-2,3j-2}} \tag{61}$$

where $\frac{1}{(\tau^{-1}-\alpha^{-1})}$ is neglected for $\tau \to 0$, which ensures numerical stability during the etching process. In the benchmark, we found out that the inherent numerical errors can negatively impact the first strategy, as suggested by Eq. (60) and will be discussed later in detail.

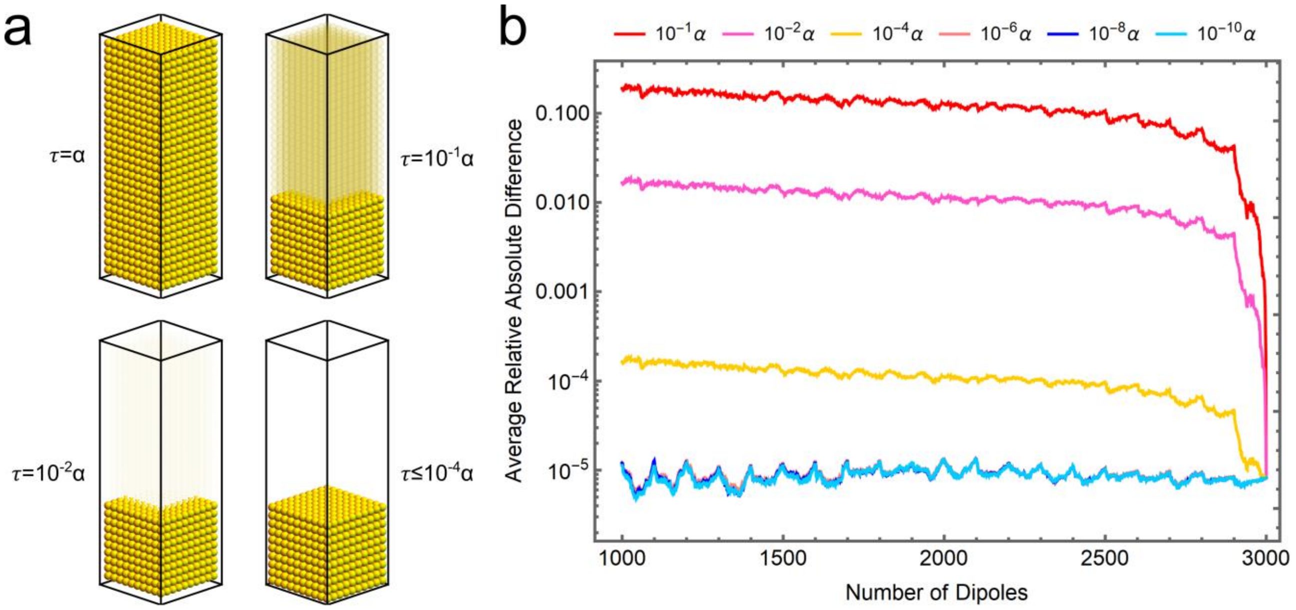

Figure S3. The error between the UV-Vis and the standard UV-Vis data with various τs. (a) The scheme of the nanocube when different τ are used to approximate the polarizability of the medium. α is the polarizability of the Au dipoles. The medium's opacity is changed according to the value of τ. The final Au nanorod was shown first. (b) The errors of the benchmark using different τs. UV-Vis spectra were generated through strategy 2, which means during the calculation, the actual initial geometry is a $10 \times 10 \times 30$ nm^3 rod with 3000 dipoles. The calculations were implemented with 64-digit numeric precision.

In strategy 2, we created a system composed of all 3000 dipoles representing the nanorod. As part of the etching process, we sequentially remove dipoles from the system by changing its polarizability α to τ, which should be close to 0 compared to the original polarizability (α) for Au dipole. The polarizations of dipoles where different τ was used to represent the polarizability of the medium (**Figure S3a**) were solved via Eq. (21)-(22), which give the extinction efficiency factors (Q_{ext}) again. Then we calculated the absolute difference between the efficiency factors solved from applying different τs and the *standard data*. The relative absolute difference is further calculated by dividing absolute difference with the *standard data*. The average of the relative absolute difference among multiple wavelengths from the trajectory is shown in **Figure S3b**. When τ is equal or smaller than 10^{-4}α, the calculated Q_{ext} converged to the *standard data*, which proves that Eq. (21)-(22) can be used as an efficient numerical approximation given that τ is smaller as compared to α ($\tau \sim 10^{-4}$α or less).

Strategy 1 was implemented by initialising the system using various τs as the polarizability of the medium. Sequentially, after changing the polarizability from a small value for τ to a constant value α for Au in the growth process, the numerical instability of the UV-Vis spectra was observed for a given small τ, which is shown in **Figure S4a**. For $\tau \leq 10^{-4}$α, such numerical instability leads to large deviations from the expected values and even unphysical NaN (infinity) values due to the presence of large values during the calculation. To dampen such problems, we reduce the order of

magnitude of **A** by multiplying it by a scale factor, so that the magnitude of the elements of **A** is closer to 1. Here the scale factor (f) is set as:

$$f = kd^3 \tag{62}$$

where d is the dipole length in the unit of meter, and k is a constant which we set as $10^{19}/m^3$ considering the dipole length is in nanoscale to make **A** as close to 1 as possible. By introducing such a scale factor, the UV-Vis spectra from the same trajectory with various τs were simulated again, as shown in **Figure S4b**. For comparison, the errors before and after rescaling **A** is shown in **Figure S4c-h**. Since there is no numerical error for $\tau \geq 10^{-2}\alpha$, rescaling the system does not influence the results (**Figure S4c-d**). By rescaling the system, the numerical instability problem can be damped. However, the errors are still large for $\tau \leq 10^{-6}\alpha$ (**Figure S4f-h**), while the errors for $\tau = 10^{-4}\alpha$ are minimized (**Figure S4e**).

Alternatively, we can increase the numeric precision during the calculation. By using 128-digit precision, the UV-Vis spectra in the growth process were simulated again. The numerical instability problem was not observed, as seen in **Figure S5a**. By applying a scale factor (f), the results did not show obvious difference, as shown in **Figure S5b-h**. Only when τ was set as $10^{-10}\alpha$, small numerical instability is observed and applying the scale factor can slightly influence the results **Figure S5h**.

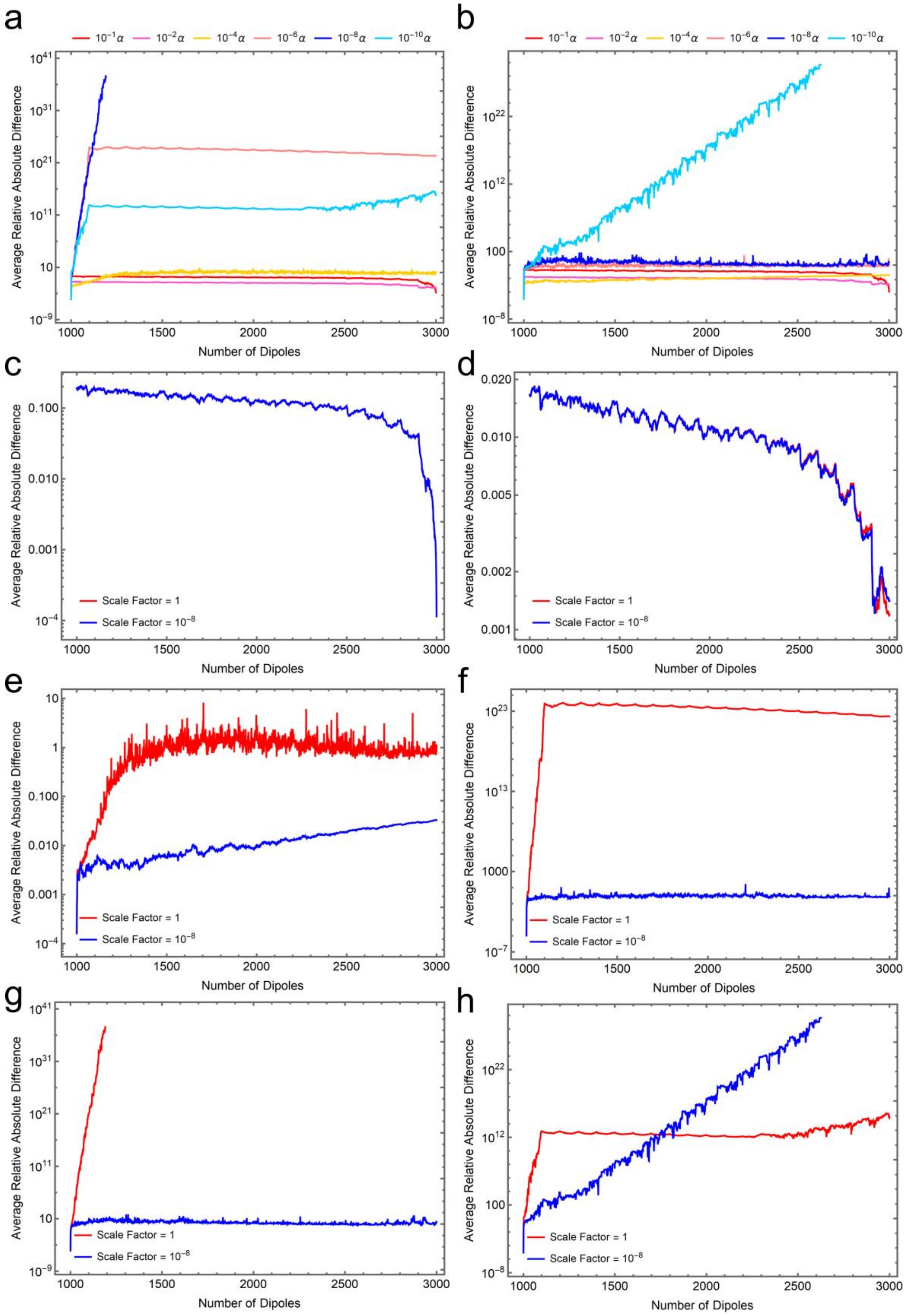

Figure S4. The average relative absolute difference between the UV-Vis and the standard UV-Vis data with various τs. (a) The error when different τ are used to approximate the polarizability of the medium. α is the polarizability of the Au dipoles. 64-digit numeric precision and a scale factor of 1 are used. (b) The error when different τ are used to approximate the polarizability of the medium. 64-digit numeric precision and a scale factor of 10^{-8} are used. The errors before and after scaling $\mathbf{A}$ matrix for $\tau = 10^{-1}\alpha, 10^{-2}\alpha, 10^{-4}\alpha, 10^{-6}\alpha, 10^{-8}\alpha, 10^{-10}\alpha$ are compared in (c) to (h).

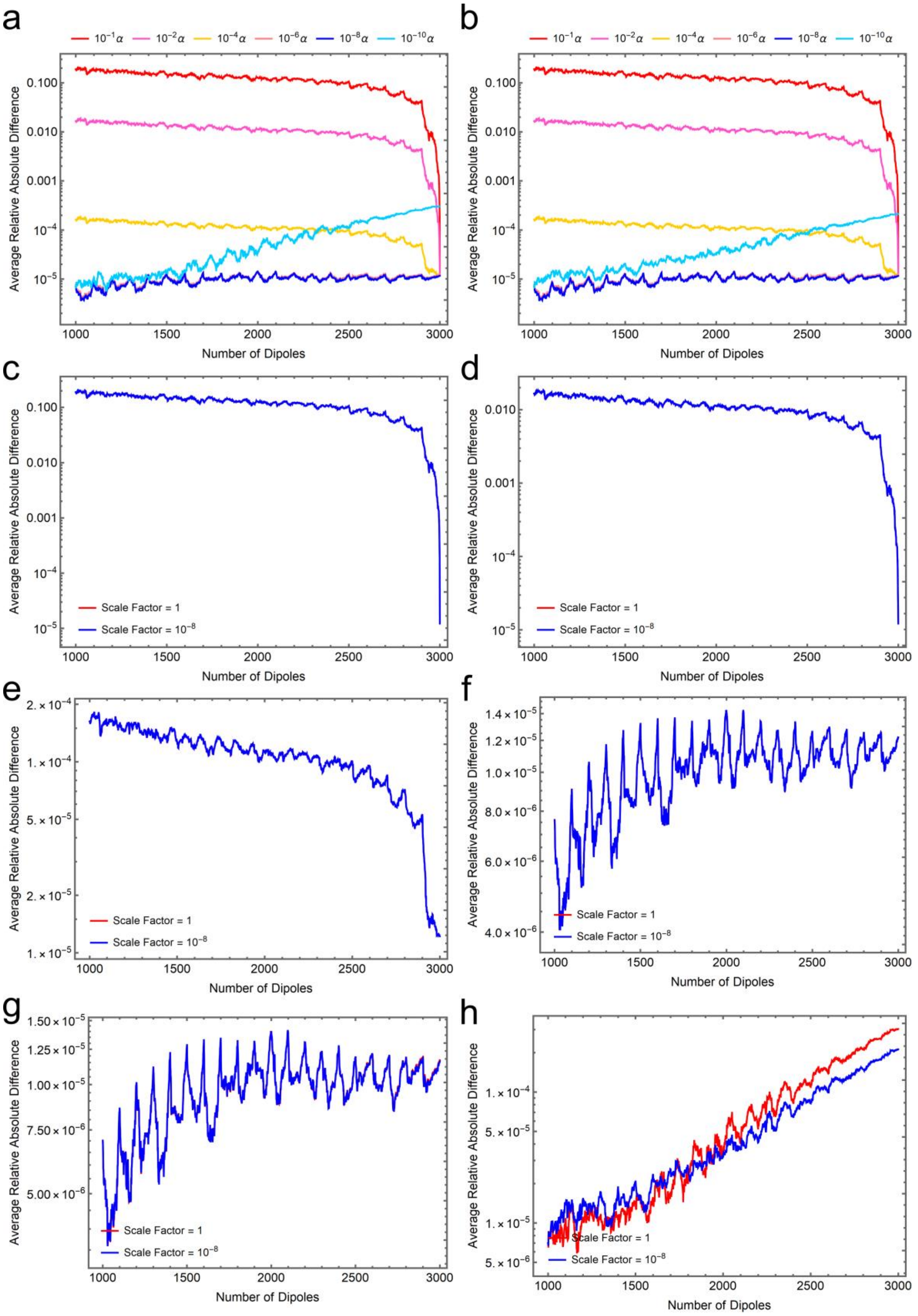

Figure S5. The average relative absolute difference between the UV-Vis and the standard UV-Vis data with various τs. (a) The error when different τ are used to approximate the polarizability of the medium. α is the polarizability of the Au dipoles. 128-digit numeric precision and a scale factor of 1 are used. (b) The error when different τ are used to approximate the polarizability of the medium. 128-digit numeric precision and a scale factor of 10^{-8} are used. The errors before and after scaling **A** matrix for $\tau = 10^{-1}\alpha, 10^{-2}\alpha, 10^{-4}\alpha, 10^{-6}\alpha, 10^{-8}\alpha, 10^{-10}\alpha$ are compared in (c) to (h).

In the benchmark calculations implementing the method described in Section 1.2, $\tau^{-1} - \alpha\tau^{-2}$ can indicates the order of magnitude of $\frac{\alpha^{-1}-\tau^{-1}}{1+(\alpha^{-1}-\tau^{-1})(\mathbf{A}^{-1})_{3j-2,3j-2}}$ for small τ during the growth process, while $\frac{1}{(\mathbf{A}^{-1})_{3j-2,3j-2}}$ is a good estimation of it during the etching process. It should be noted the actual value of $\frac{\alpha^{-1}-\tau^{-1}}{1+(\alpha^{-1}-\tau^{-1})(\mathbf{A}^{-1})_{3j-2,3j-2}}$ should always be used in the calculations, while $\frac{1}{(\mathbf{A}^{-1})_{3j-2,3j-2}}$ and $\tau^{-1} - \alpha\tau^{-2}$ helped to understand the numerical error.

In the etching case, $\tau = 10^{-8}\alpha$ and 64-digit numeric precision was used in the etching process to enable numerical stability. **A** matrix was not rescaled during the calculation. The calculated factor ($\frac{\alpha^{-1}-\tau^{-1}}{1+(\alpha^{-1}-\tau^{-1})(\mathbf{A}^{-1})_{i,i}}$) is almost identical to $\frac{1}{(\mathbf{A}^{-1})_{i,i}}$, with only neglectable differences as shown in **Figure S6a**. The index i corresponds to the matrix indexes relevant to the 6000 updates needed to etch away the 2000 dipoles in the X, Y and Z directions.

For the growth process, we compared the order of magnitude between $\frac{\alpha^{-1}-\tau^{-1}}{1+(\alpha^{-1}-\tau^{-1})(\mathbf{A}^{-1})_{i,i}}$ and $\tau^{-1} - \alpha\tau^{-2}$ when the calculation is numerically stable for a series of τs (i.e., using 128-digit numeric precision). Again the index i corresponds to the matrix indexes relevant to the 6000 updates needed to grow the 2000 dipoles. τ was varied from $10^{-1}\alpha$ to $10^{-10}\alpha$, and its corresponding estimation value ($\tau^{-1} - \alpha\tau^{-2}$) as well as the actual value of $\frac{\alpha^{-1}-\tau^{-1}}{1+(\alpha^{-1}-\tau^{-1})(\mathbf{A}^{-1})_{i,i}}$ during the growth were recorded. It is observed the order of magnitude of $\frac{\alpha^{-1}-\tau^{-1}}{1+(\alpha^{-1}-\tau^{-1})(\mathbf{A}^{-1})_{i,i}}$ increases when smaller τ is applied and shows similar order of ($\tau^{-1} - \alpha\tau^{-2}$), as shown in **Figure S6b**.

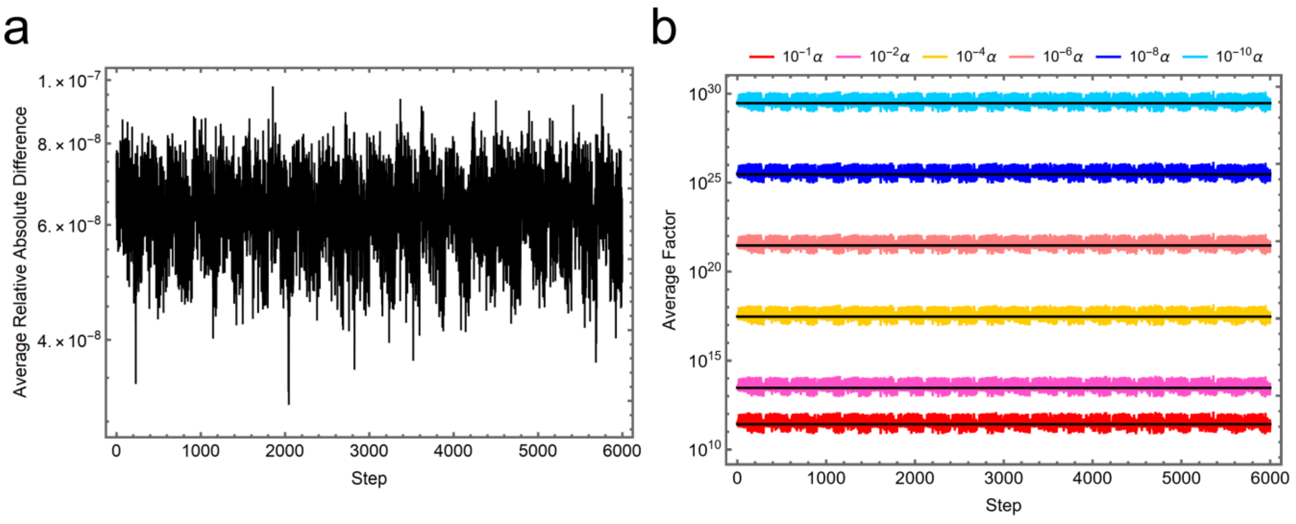

Figure S6. The convergent and divergent behaviour of the factor of $\frac{\alpha^{-1}-\tau^{-1}}{1+(\alpha^{-1}-\tau^{-1})(\mathbf{A}^{-1})_{i,i}}$ in the etching and growth processes respectively. (a) The average relative absolute difference between

the calculated factor $\frac{\alpha^{-1}-\tau^{-1}}{1+(\alpha^{-1}-\tau^{-1})(\mathbf{A}^{-1})_{i,i}}$ and $\frac{1}{(\mathbf{A}^{-1})_{i,i}}$. (b) The logarithmic plot for the averaged magnitudes calculated from the factor $\frac{\alpha^{-1}-\tau^{-1}}{1+(\alpha^{-1}-\tau^{-1})(\mathbf{A}^{-1})_{i,i}}$ (labelled in different colours) and the estimated $\tau^{-1} - \alpha\tau^{-2}$ (black lines) to show the similarity of the orders. In both plots, the value is averaged among 51 wavelengths from 400 nm to 900 nm with an interval of 10 nm.

In summary, we have discussed RD-DDA from various perspectives. For a replacement process, the method described in Section 1.2 gives the exact and computationally efficient strategy to obtain the new solutions. For a growth process, Section 1.3 gives the exact solution to track the dipole change but the change of matrix size may dampen computation efficiency. Implementing the method described in Section 1.2 by setting a small polarizability of the medium can avoid the change of the matrix size, but can also cause numerical instability, so higher (128-digit) precision during the calculation is recommended. For an etching process, the exact solution to track the removal of a dipole is discussed in Section 1.4, with the varied matrix size. The method described in Section 1.2 with a small polarizability of the medium can be a good approximation to the exact solutions and ensures numerical stability in the etching process but not the growth process. However, for any growth process, an equivalent etching process can be created by reversing the trajectory, indicating that the system for the growth process can be solved by solving it as an etching process.

By far, we have discussed both general analytical solutions to track the dipole change under replacement, addition, and removal, and their computationally efficient implementation. The computational time from RD-DDA and from directly solving a new system in every step will be estimated, compared, and discussed below.

1.6. The benchmark of the computational time for the direct solutions from DDA and the iterative solutions from RD-DDA

Considering the chemical reactions that change the morphology of plasmonic nanoparticles in an atomic- or nano-scale, their optical properties can be altered in this process. If the size of the dipole is relatively small compared to the overall size of the nanostructure, the morphological change can be described as an addition, removal or replacement of dipoles. A general strategy to observe the spectral properties in this process is to sample multiple intermediate structures and simulate their spectral properties respectively. RD-DDA accelerates the simulation process dramatically by avoiding solving for polarizations of each intermediate separately. Here, we compared the time efficiency of our algorithm, by estimating the computational time of solving the intermediates directly, as well as using RD-DDA. The benchmark calculations were performed on the same GPU of NVIDIA® TITAN RTX™ with TensorFlow (>2.0).

A system with the growth process of a single surface layer of Au@Ag octahedra was used to

demonstrate the computational efficiency. We estimated the formation of a single layer of Ag on Au@Ag octahedra with an edge length of 20 nm. The Au core is discretized and represented by a series of 1 nm dipoles. Then a thin layer of Ag dipoles with a size of 1 nm was grown on the surface by random addition. The number of dipoles was changed from 3303 to 4089 by the sequential addition of dipoles on the surface randomly.

The polarizations of the dipoles of the intermediates can be solved directly through inversing **A** matrix or other iterative methods[8] or using RD-DDA. For benchmarking, we solved the system directly by inversing **A** matrix as a reference. Instead of solving the intermediates directly, we estimated the time of solving the largest and smallest nanostructures (the initial octahedra and the final octahedra respectively). Since the computational time is dependent on the size of the system, it was used as an estimation for the computation cost for the direct solutions by implementing the DDA directly.

Then we implemented RD-DDA in two different ways with both 64-digit and 128-digit precision:

1. Solving the system analytically by expanding the system as described in Section 1.3, which is labelled as rank-one decomposition iterative solution 1(RS 1).
2. Attributing a small polarizability ($\tau = 10^{-10}\alpha$) to define the dipoles representing the medium. Create an etching process trajectory by reversing the growth trajectory and removing dipoles by changing the corresponding polarizability from α to τ (see Section 1.5), which is labelled as rank-one decomposition iterative solution 2 (RS 2). This setting is due to the numerical instability problem described above.

We defined an acceleration factor (F_{acc}, Eq. (63)) to quantify the computational efficiency of RD-DDA as compared to the direct solution method (implementing the DDA directly):

$$\mathrm{F}_{acc} = \frac{t_D}{t_R} \tag{63}$$

where t_D and t_R defines the computational time for the direct solutions and the iterative solutions from RD-DDA respectively. t_D was calculated from the average of the upper/lower boundary of the time cost of DS. After simulating the overall trajectory, RS 1 and RS 2 showed ca. 46- and 98-times acceleration for 64-digit precision, and 180- and 250-times acceleration for 128-digit precision. Additionally, since 64-digit precision is generally good enough for DDA, the time cost when the direct solution method was implemented using 64-digit precision and RD-DDA was implemented on 128-digit precision respectively was further compared (**Figure S7**), showing 24- and 33- times accelerations for RS 1 and RS 2 respectively.

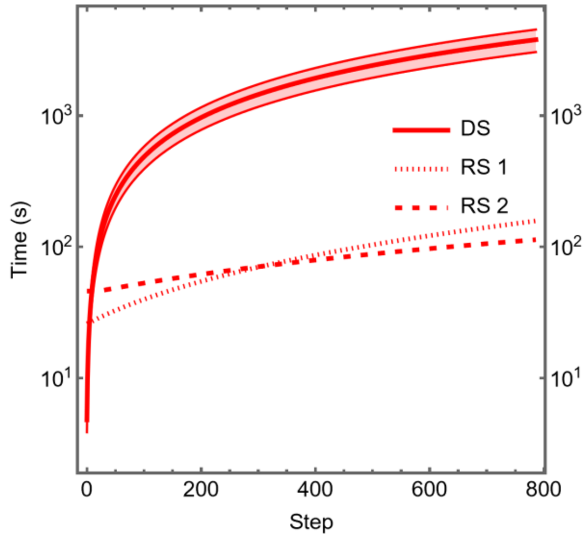

Figure S7. The benchmark of coating Ag layer on Au octahedra. The direct solution was implemented with 64-digit precision and RD-DDA (for both RS 1 and RS 2) was implemented with 128-digit precision.

An additional example to quantify the computational efficiency consists of the growth process of nanospheres from a radius of 10 nm to 11 nm, which was benchmarked similarly as described above. The sequential addition of 1 nm dipoles occurred in the ascending order of the distance between the dipoles and the centre of the initial sphere. The initial and the final number of dipoles were 4169 and 5575 respectively, and the corresponding structures were used to estimate the time cost for the direct solutions. The calculations were performed with a 64-digit precision. RS 1 and RS 2 showed ca. 53 and 101 times acceleration.

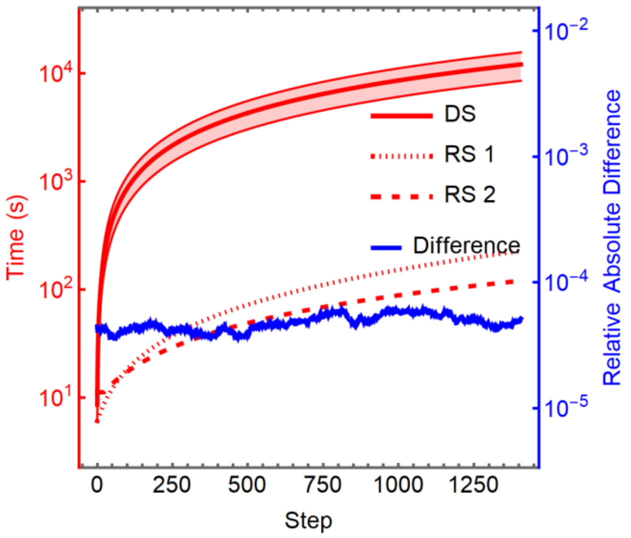

Figure S8. The benchmark of the growth of small nanospheres to large nanospheres. The computational times from different methods are shown.

As demonstrated above, RD-DDA is very powerful when the optical properties of a large number of intermediates need to be calculated. However, for some applications, not as many intermediates are needed and a comparison between using the direct solution method or RD-DDA will be made. If label

the time cost to solve the largest and smallest nanostructures as(t_{lower}, t_{upper}), the time cost to solve M linear systems using the direct solution method can be estimated as $[M \times t_{lower}, M \times t_{upper}]$. For RD-DDA, its time cost is constant if we wish to evolve from the initial structure to the final structure. For the case of coating Au octahedra to form Au@Ag nanostructures as discussed above, the comparison of time cost to simulate N intermediates using the direct solution method and RD-DDA is shown in **Figure S9**, with different numerical precisions. When both methods are set in 64-digit precision, the acceleration factor (F_{acc}, Eq. (63)) is larger than one when the number of intermediates is larger than 18 and 9 for RS 1 and RS 2 respectively. When the methods are set to 128-digit precision, F_{acc} is larger than one if the number of intermediates exceeds 5 and 4 for RS 1 and RS 2 respectively. When RD-DDA is in 128-digit precision and the direct solution method is in 64-digit precision, more intermediates are required to make F_{acc} larger than one, which is 33 and 24 for RS 1 and RS 2 respectively. It should be noted that in this comparison, the total number of the solved systems will be equal to the number of intermediates plus one (which corresponds to the initially solved nanostructure in RD-DDA).

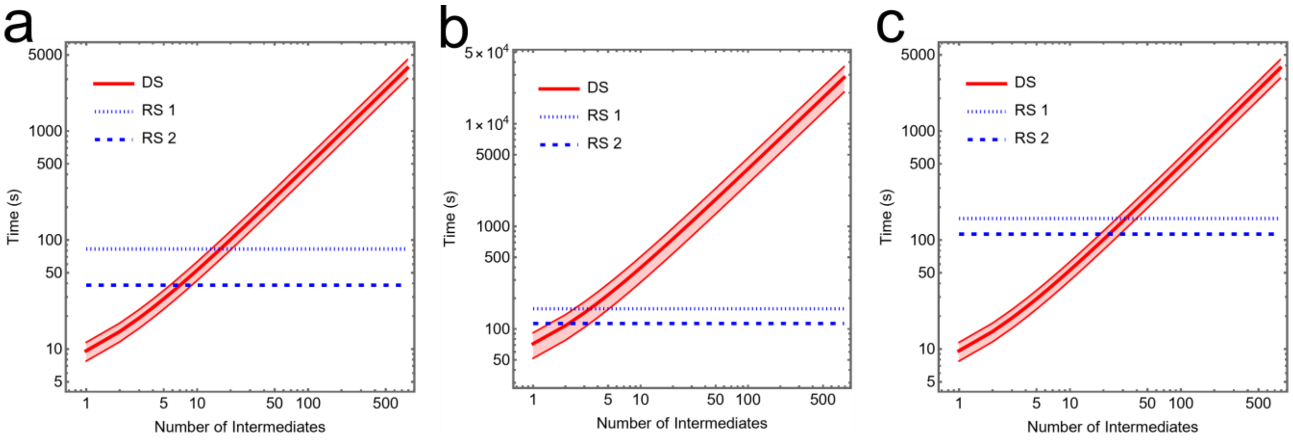

Figure S9. The comparison of the time cost when the different numbers of intermediates are needed for the simulation of Ag coating on Au octahedra. The computational times from different methods are shown. RD-DDA and the direct solution method were implemented in the same 64-digit precision in (a) and 128-digit precision in (b) respectively. In (c), RD-DDA was implemented with 128-digit precision and the direct solution method was implemented with a 64-digit precision.

For the second case of growing Au nanospheres to larger nanospheres, a similar comparison can be made (**Figure S10**). Here, F_{acc} is larger than one if the number of intermediates exceeds 27 and 14 for RS 1 and RS 2 respectively.

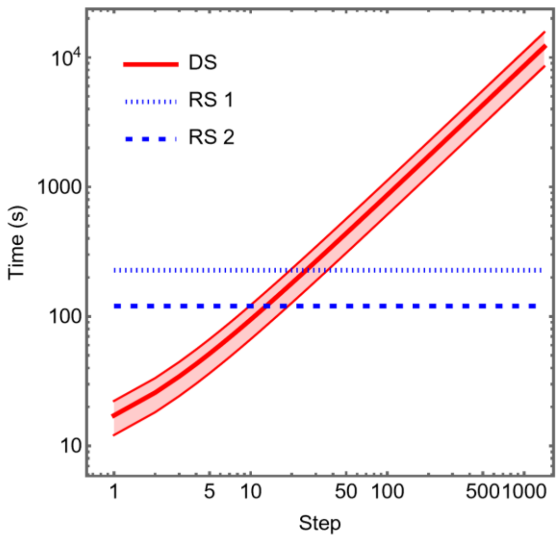

Figure S10. The comparison of the time cost when the different numbers of intermediates are needed for the simulation of Au nanosphere growth. The computational times from different methods are shown. RD-DDA and the direct solution method were implemented using the same 64-digit precision.

1.7. Estimating the time cost of the direct solution method and RD-DDA

In this section, we will discuss a general strategy to estimate the time cost for both methods including the direct solution method and RD-DDA. Since the computation is mainly matrix manipulations, we can assume the time cost for every update in RD-DDA depends mainly on the matrix size (or equivalently, the dipole number).

First, the time cost for the direct solution method can be estimated quite straightforwardly. The largest (assume it consists of N_{upper} dipoles) and smallest structures (assume it consists of N_{lower} dipoles) during the evolving process should first be found. Then DDA can be implemented on them directly with the time cost recorded, which corresponds to the estimated upper/lower boundary of the time cost (t_{upper}, t_{lower}) for one structure using the direct solution. If we have M nanostructures to calculate in total, the time cost will be estimated in the range of $[M \times t_{lower}, M \times t_{upper}]$ for the direct solution.

In the meantime, with the benchmark cases using RD-DDA simulation discussed in the previous section, we observed the time cost to obtain one solution recursively after one dipole change is linearly correlated to the number of existing dipoles in the system (equivalently, the matrix size), as shown in **Figure S11**.

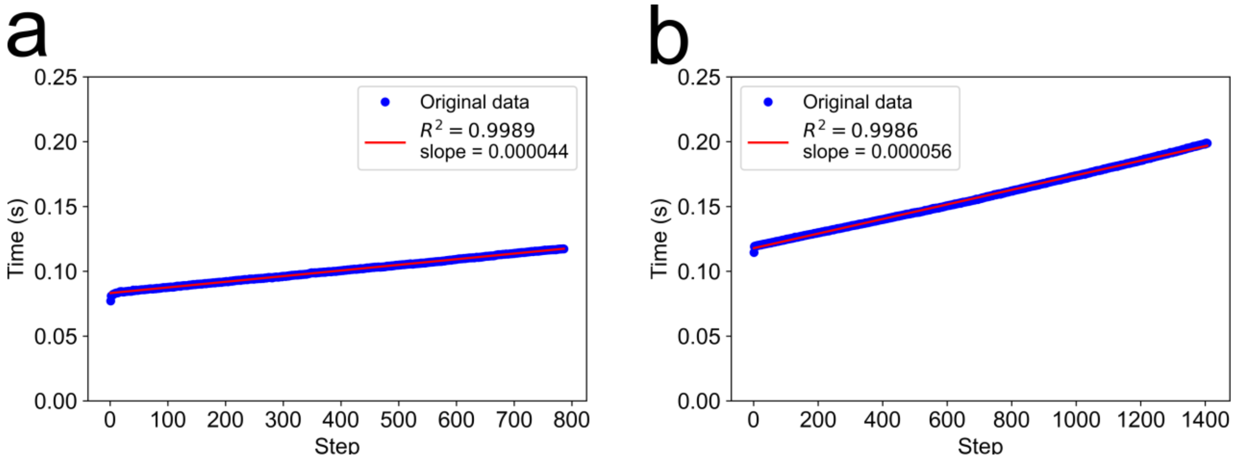

Figure S11. The raw data of the time cost to obtain a new solution in RD-DDA and its corresponding linear fitting. (a) The time cost and its linear fitting to perform one update (i.e., to solve one new system after the change of one dipole) using RD-DDA during the coating of Ag on the surface of Au octahedra with RS 1. The same raw data is used in **Figure 1b** in the manuscript. (b) The time cost and its linear fitting to perform one update in the growth of Au nanospheres with RS 1. The same raw data is used in **Figure S8**.

Thus, by assuming the time cost for one update (after a dipole change) towards a different nanostructure is linearly correlated to the existing dipole number (thus the matrix size), one possible strategy to estimate the time cost for RD-DDA is proposed as follows:

1. If we know the trajectory, we can estimate the lowest and highest time cost for one update by performing the update on the smallest system (with N_1 dipoles) and the largest system (with N_2 dipoles) where the update can happen, which will give the lower/upper time cost estimation of one update as t_1 and t_2, respectively.
2. Then we can create a linear curve using $(N_1, t_1)$and (N_2, t_2). The linear curve will be used to estimate the time cost (t_i) when we perform one update on the intermediate with a dipole number of N_i.
3. When we perform RD-DDA, we will solve the initial structure with a time cost t_{init}. Then the overall time cost for RD-DDA would be $t_{RD-DDA} = t_{init} + \sum_i t_i$, where t_i is the time cost for acquiring the new solution after the change of one dipole in the trajectory, and can be obtained efficiently from the linear curve from Step (2). With this method, t_{init} is precise and $\sum_i t_i$ is estimated.

We will validate if the linear interpolation strategy is good to estimate the RD-DDA time cost in the two benchmark cases described above. Specifically, we will estimate the time cost ($\sum_i t_i$) for all the possible small trajectories from the whole growth trajectories for validation. These small trajectories include at least two updates to create the linear curve.

On the one hand, we have recorded the actual update time cost for these small trajectories (i.e., the

time cost for RS 1 in **Figure 1b** and **Figure S8**). On the other hand, we have also recorded the time cost for one update for the smallest and largest structures (two points), which can be used to create a linear curve to estimate the $\sum_i t_i$ term as described above. By comparing the actual and estimated time cost for all the small trajectories, the relative error due to the estimation can be calculated (see **Figure S12**). The maximal relative error from the estimation is 3.77% for the case of the growth of Ag on the surface of Au octahedra and 2.00% for the case of the growth of nanospheres, respectively.

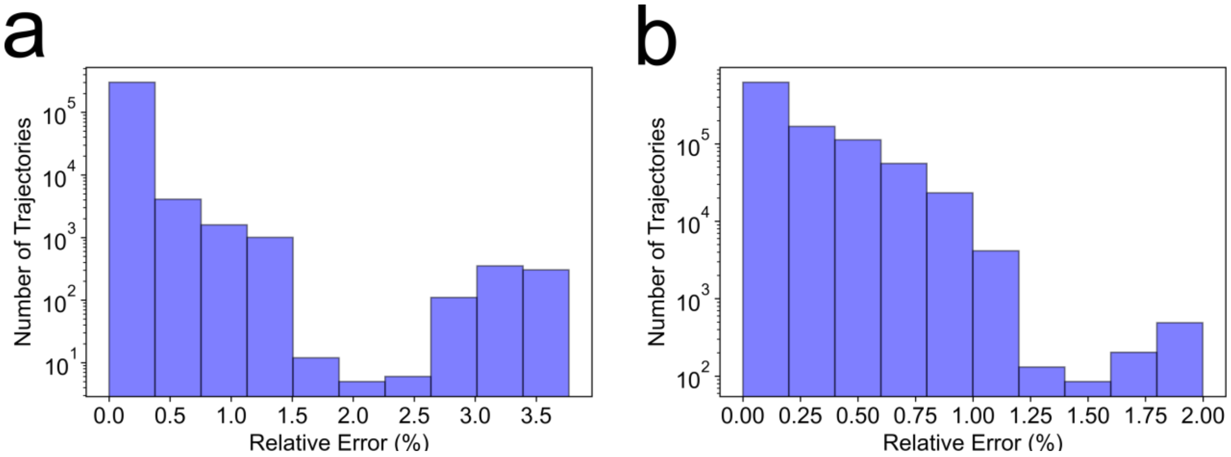

Figure S12. Histogram of the relative error for the time estimation ($\sum_i t_i$) for all possible small trajectories. (a) The relative error distribution of the estimated time for the small trajectories from the growth of a Ag layer on Au octahedra. The total number of small trajectories is 308505, with a maximal relative error of 3.77%. (b) The relative error distribution of the estimated time for the small trajectories from the growth of Au nanospheres. The total number of small trajectories is 987715, with a maximal relative error of 2.00%. It should be noted that if the number of updates is two for the small trajectory, $\sum_i t_i$ will be the precise time cost and will give a relative error of 0. The number of such "two-update" trajectories is 785 and 1405 for (a) and (b), respectively.

Finally, for comparison, if t_{RD-DDA} is smaller than $M \times t_{lower}$ (from the estimation of the direct solution method), RD-DDA will be more efficient. When t_{RD-DDA} is larger than $M \times t_{upper}$, RD-DDA is not suitable for this application. However, when t_{RD-DDA} is in the range of $[M \times t_{lower}, M \times t_{upper}]$, it will depend on the specific intermediates and the algorithm used to solve the linear system. One simple solution is to use the average of $M \times t_{lower}$ and $M \times t_{upper}$ to see if RD-DDA is more efficient. In the meantime, a more precise comparison will need to estimate the time cost to solve the intermediates. For instance, if we assume the time cost to solve a linear system is proportional to n^3 (where n is the matrix size) for simplicity, we need to get the number of dipoles for the intermediates and estimate the time cost for every intermediate by linear interpolation using $({N_{lower}}^3, t_{lower})$ and $({N_{upper}}^3, t_{upper})$. Then we can estimate the time cost for solving these N intermediates more accurately. Eventually, comparing it with t_{RD-DDA} can tell if we should use RD-DDA or the direct solution method.

2. Efficient tracking of UV-Vis spectra using the RD-DDA

In the last section, we formulated a rank-one decomposition method to accelerate the discrete-dipole approximation for dynamically evolving nanostructures. Using this approach, it is possible to efficiently track the spectral properties like UV-Vis spectra of nanostructures for any given morphological change. In this section, we create trajectories defining morphological changes in three different cases:

1. Custom-built trajectories corresponding to different growth modes.
2. A trajectory generated from an empirical crystallographic surface growth model.
3. A trajectory generated from an atomic model using the kinetic Monte Carlo simulation.

2.1. Custom-built trajectories

In this case, we studied the growth of a thin layer of Ag on the surface of Au octahedra. It was achieved by adding a layer of 1 nm Ag dipoles to the surface of the Au core. The Au core is created by a series of dipoles composed of pure Au atoms with a dipole length of 1 nm. The edge length of the final octahedral Au@Ag core-shell nanostructure is 20 nm. By varying the growth strategies, three types of trajectories were generated.

1. Adding the layer of Ag dipoles randomly (labelled as **Random**).
2. Adding the layer of Ag dipoles in ascending order of the distance from the centre of the surface (labelled as **Centre**).
3. Adding the layer of Ag dipoles in descending order of the distance from the centre of the surface (labelled as **Tip**).

The growth trajectories with random dipole addition were generated 10 times to create statistically significant results. During the growth process, the number of dipoles increased from 3303 to 4089. An etching trajectory generated by reversing the growth trajectory was used to calculate the polarizations with a 64-digit precision. The polarizability of the medium is set as $\tau = 10^{-10}\alpha$. It should be noted the changed dipoles were purely composed of Ag, so α specifies the polarizability of Ag dipoles in this case.

The corresponding UV-Vis spectra can thus be simulated using RD-DDA (See Figure 2 in the manuscript). The full details of the spectra are available in Supplementary Information (SI) Video S1-S3.

2.2. An empirical crystallographic surface growth model

Crystallographic surface growth models are usually empirical but powerful to elucidate the morphological changes during crystal growth. Previously, using Transmission Electron Microscopy

(TEM) the morphological transformation of Au arrow-headed nanorods into Au octahedra[9] has been investigated. Inspired by this experimental observation, here we constructed a trajectory using an empirical model by defining the growth of multiple crystal surfaces to describe this transformation. Once the trajectory was generated, the UV-Vis spectra of the intermediates were calculated.

The initial arrow-headed nanorods were enclosed within two types of crystallographic surfaces: (110) and (111)[9]. We set constant growth rates for these surfaces as k_1 and k_2 respectively. The region of the nanostructure is defined by the following enclosed planes in the space:

$$|X| + |Y| \leq k_1 t + b_1 \tag{64}$$

$$|X| + |Y| + |Z| \leq k_2 t + b_2 \tag{65}$$

where k_1 and k_2 are the growth rate for (110) and (111) surfaces, $b_1$and b_2 are the initial conditions. t is a non-negative integer defining the growth step. We set $k_1 = 0.0205\ nm/step$, $k_2 = 0.01k_1$ to ensure that we can capture the atomic layer growth precisely and faster growth rate of (110) surface, and $b_1 = 2.87\ nm$ and $b_2 = 6.56\ nm$ to generate an initial arrow-headed rod shape.

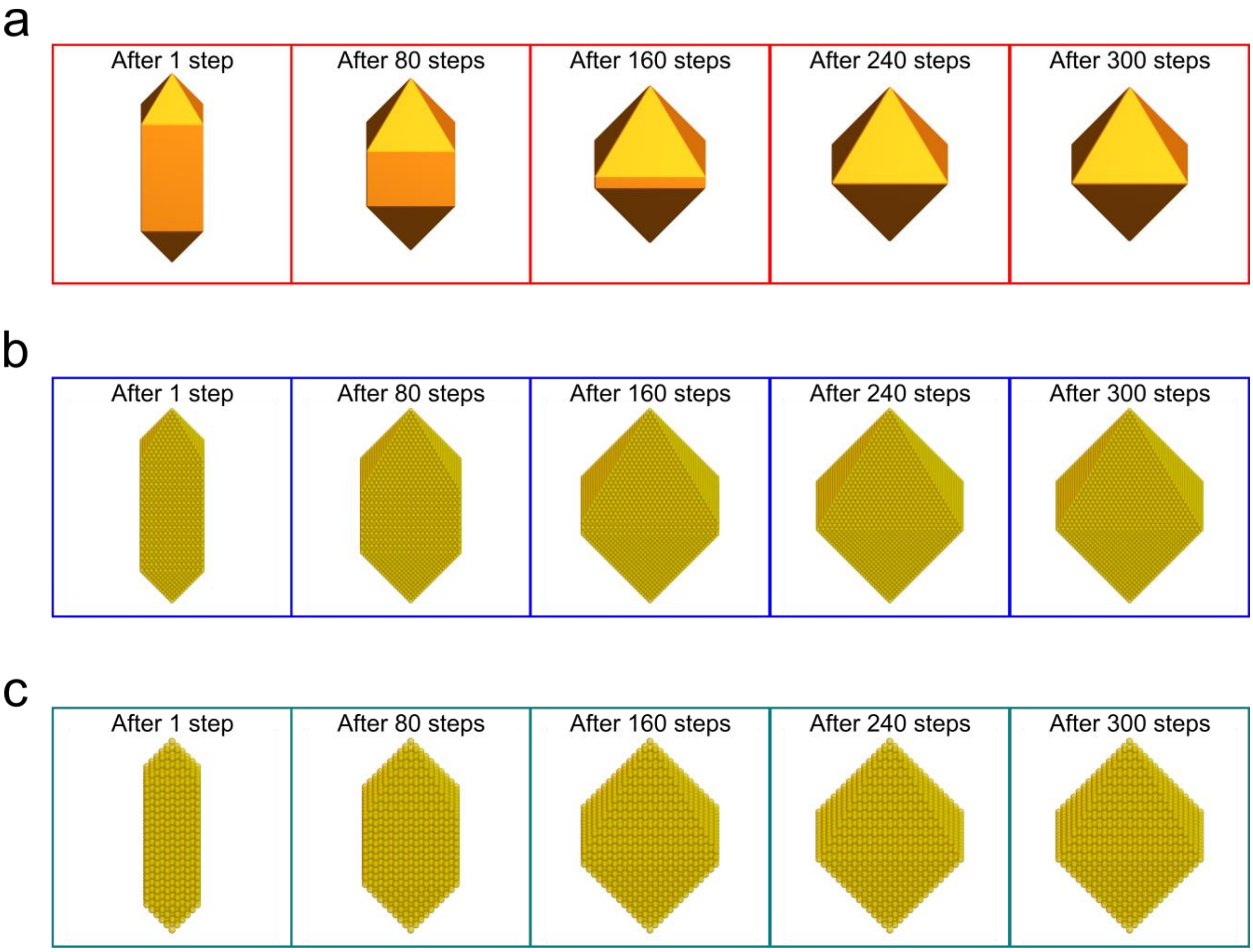

Figure S13. The growth process from Au arrow-headed rods to Au octahedra (a) Continuous region representation of the growth process. (b) The corresponding atomic representation of the

growth process. (c) The corresponding discrete dipole representation with the length of a dipole as 0.41 nm for the same process.

The growth process was simulated up to 300 steps (including the first step where $t = 0$ to generate the initial arrow-headed rod) which were enough to convert the arrow-headed nanorod into an octahedron completely. To calculate UV-Vis spectra using the RD-DDA method, the bounded region representing the nanostructure was discretized into dipoles, with a dipole length of 0.41 nm, which is approximately equal to the lattice constant of Au. This growth process generated a set of intermediate structures which we labelled as $\boldsymbol{S} = \{S_0, S_1, \dots, S_t, \dots, S_{t_{max}}\}$, where S_t indicates the structure at time step t, and $S_{t_{max}}$ represents the final structure. It should also be noted due to precise and fine sampling of intermediate structures in 300 steps, some of the consecutive intermediate structures could share identical atomic distribution.

The fine sampling in the growth model equivalently represents the atomic layer growth which is shown in **Figure S13a-c**, where the intermediates after every 80 growth steps together with the initial Au arrow-headed rods and the final octahedra are shown. However, at the atomic scale, simultaneous growth over the complete crystallographic plane represents an ideal process. In general, at the atomic scale, the crystal growth is a stochastic process, where the order of sequential addition of atoms towards a single atomic layer growth to the crystallographic surface of the nanostructure could potentially create different trajectories. Here, we generate various growth trajectories by shuffling the sequence of the addition of atoms for the same layer to investigate the bounds on the spectroscopic deviation due to stochasticity in the growth process at the atomic scale. The way of generating these trajectories is described below:

1. Since we implemented a continuum crystal surface growth model with atomic layer precision, an extra layer of atoms can be added after one step. We labelled the structure before and after adding this layer as S_t and S_{t-1}.
2. The atomic growth trajectory was then generated by randomly adding the extra atoms one by one sequentially, which enables the transformation from S_{t-1} to S_t. However, the sequence of adding these atoms can be different, which generates different growth trajectories from S_{t-1} to S_t.
3. After the addition of a single atom, the influence on the structure's dipole representation should be checked, which can cause the addition of one dipole or dipoles. If a face-centred lattice is fully occupied after adding the atom, a dipole representing this lattice should be added. If the addition of one atom causes the addition of multiple dipoles, they will be added sequentially with a shuffled sequence. Thus, multiple trajectories composed of the sequential addition of dipoles for the transformation from S_{t-1} to S_t can be generated, which

approximates the atomic growth process.

4. The overall trajectory from S_0 to S_{max} can be generated by combining the trajectories to transform S_0 to S_1, then S_1 to S_2,…, and eventually $S_{t_{max}-1}$ to $S_{t_{max}}$.

For the RD-DDA calculations, the polarizability of the medium was set as $\tau = 10^{-10}\alpha$ with 64-digit numeric precision, where α is the polarizability of a 0.41 nm dipole purely composed of Au. The wavelength was sampled from 400 nm to 900 nm with an interval of 10 nm. We reversed the growth trajectory to avoid the numeric instability problem during the calculation.

In the simulated spectra, the transverse and longitudinal modes of the original Au arrow-headed rods were observed which are typical spectral features for anisotropic nanoparticles. When the arrow-headed rods transformed into the Au octahedra leading to an isotropic shape, these modes merge into a single peak. Their peak prominence also varies over the growth process, as shown in **Figure S14**. It is observed that the complete coating of a compact layer of dipoles on the surface can enhance the extinction of the longitudinal mode. The full details of the spectra from one trajectory are available in SI Video S4-S5.

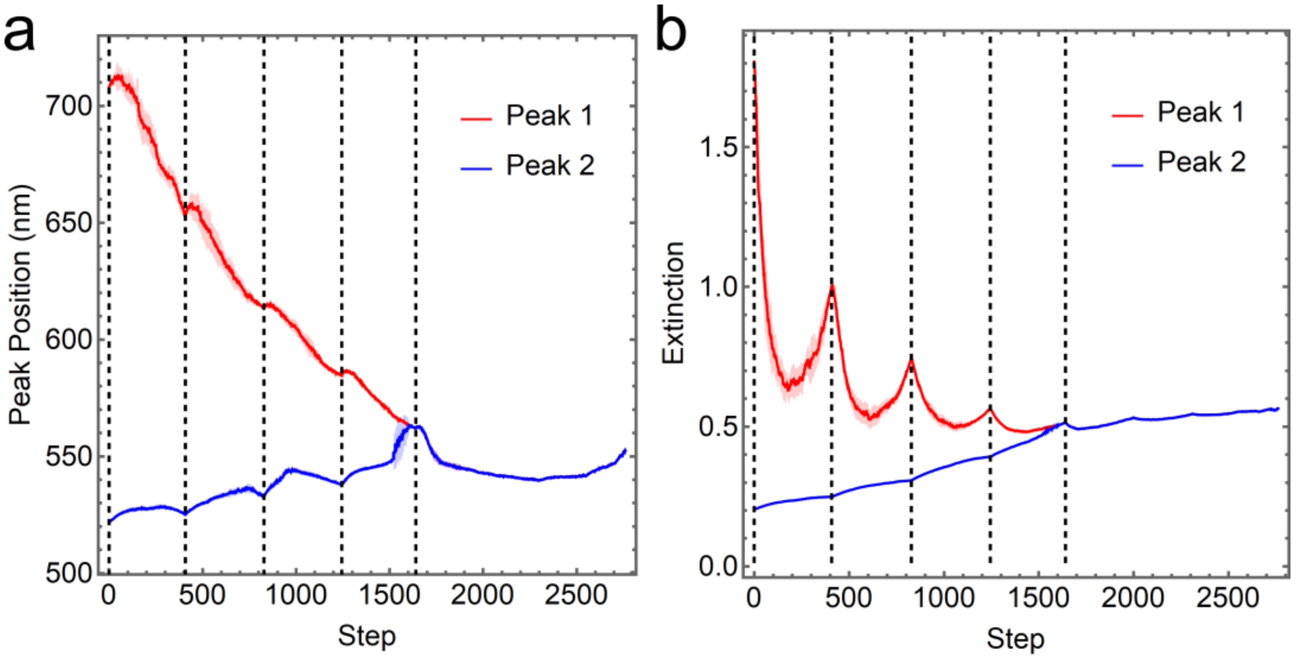

Figure S14. The change of the peak positions and prominences during the growth of Au arrow-headed rods. The peak positions and prominences in the process are shown in (a) and (b) respectively. The results were averaged by 10 trajectories including the addition of 2760 dipoles to the initial arrow-headed rod; thus 2760 steps are shown here. It should be noted these steps are different from the steps in the continuous crystal growth model. Peak 1 and 2 correspond to the longitudinal and transverse peaks respectively.

2.3. Atomic-scale models of nanostructural transformation using kinetic Monte Carlo simulation

2.3.1. Au nanostructures

The Monte Carlo simulation to investigate nanostructural transformations is based on the previous study[10], and only its implementation is been discussed here.

The Monte Carlo method includes two types of possible events:

1. The addition of a single Au atom on the surface vacant sites.
2. The removal of a single Au atom from the surface of the nanostructure.

The probability of these two events depends on the difference between the chemical potentials at the surface and the environment (e.g., the solution phase). Each step in the Monte Carlo Simulation consists of the following sub-steps:

1. Sample the event type with equal probability: addition or removal
2. If an addition event is sampled, select a vacant site from all the available vacant sites. The probability of addition of the atom on the selected site is calculated as:

$$p = \frac{1}{1 + 1/fc} \tag{66}$$

$$fc = \frac{N_{vac}}{N_{surf}} e^{-\beta(-\mu-\varepsilon n)} \tag{67}$$

   where N_{vac} and N_{surf} are the numbers of vacant/surface sites of the current structure, μ is the chemical potential difference of the atom between the solution and the surface of the nanostructure, ε is the binding energy from the neighbouring atom, n is the coordination number of the site after adding one atom, and $\beta = \frac{1}{k_B T}$ with k_B is the Boltzmann constant and T is the temperature.

3. If a removal event is sampled, select a surface site from all the available surface atoms. The probability of removal of the atom on the selected surface site is calculated as:

$$p = \frac{1}{1 + 1/fc} \tag{68}$$

$$fc = \frac{N_{surf}}{N_{vac}} e^{-\beta(\mu+\varepsilon n)} \tag{69}$$

4. Steps 1-3 are repeated until the simulation is finished.

It should be noted that compared to the original method, we used the ratio of the current surface/vacant

sites to approximate the detailed balance to accelerate the simulation. In the original work, they used the sites before/after the Monte Carlo steps for the detailed balance. Due to the relatively large numbers of the sites, this approximation holds. The surface sites were found by selecting atoms with coordination numbers smaller than 12, while the surface vacant sites were in the surroundings of existing atoms. The etching process of Au octahedra with an edge length of ~ 9.3 nm was selected as an example. In the simulation, we set $k_B T = k_B \times 300K = 0.0259\ eV$, $\varepsilon = 0.3275\ eV$ and $\mu = -6\varepsilon = -1.965\ eV$. Approximately two million Monte Carlo steps are required to etch the octahedra into spheres.

Starting with well-defined Au octahedra, the kinetic Monte Carlo simulation was performed three times with the same parameters but with varied random seeds for sampling. An example trajectory, with atomic-scale variations during the Monte Carlo simulation, an equivalent trajectory at dipole scale, and their corresponding UV-Vis change are shown in SI Video S6-S7.

To find the equivalent dipole transformation of the atomic transformation, we first generated a set of dipoles representing the initial Au octahedron. After the Monte Carlo simulation, it was observed that these initial dipoles included all the dipoles involved in the structural transformation. Thus, these initial dipoles were used to calculate the **A** matrix. Then, we recorded the addition/removal of atoms, and if the dipole was not fully occupied, it was regarded to represent the medium, which generate the trajectory of dipole transformation. During the dipole transformation, when a dipole is removed or added, its polarizability is changed between α and τ accordingly.

It should be noted here we directly removed or added a dipole, thus the polarizability is changed between α and τ. However, if the dipole is not fully filled with atoms, a series of intermediate polarizabilities (α_{inter}) can be estimated based on the number of atoms within the dipole. The scattering signals when the polarizability of the dipole is changed among α_{inter}, α and τ can also be tracked with the proposed method above. This strategy will be discussed in detail when the replacement event happens in the example below (Section 2.3.2).

During the simulation, 128-digit precision is used. $\tau = 10^{-6}\alpha$ is used to define the polarizability of the medium. A cubical volume of face-centred cubic (FCC) lattice with a length of 0.41 nm was set as a single dipole, and when the lattice is fully occupied, the polarizability will be set as α, otherwise τ. Using the RD-DDA simulation, we sampled at the wavelength from 450 nm to 700 nm with an interval of 10 nm, with additional points between 520 nm and 570 nm with an interval of 2 nm to precisely capture the peaks. The simulated UV-Vis spectra are shown in **Figure S15**.

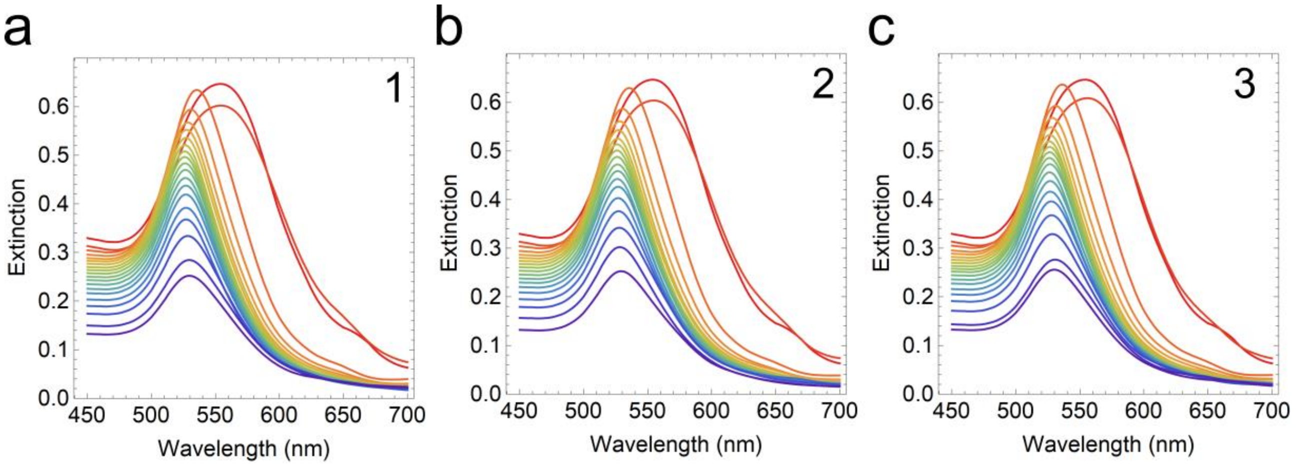

Figure S15. The UV-Vis change from three different trajectories labelled from 1 to 3. The spectra from the trajectory 1, 2 and 3 are shown in (a), (b) and (c) respectively. The three repeats all showed a consistent tendency of the UV-Vis change for the transformation.

2.3.2. Au-M nanostructures

In this section, we extended the kinetic Monte Carlo combined with the RD-DDA approach to multi-metallic systems. In the case of a bimetallic system with gold as one metal, we labelled the first atom type as Au, while the second type as M. We assume that the metal M shares a similar lattice constant and packing type as Au, i.e., face-centred cubic lattice. The chemical potentials of Au/M, as well as the Au-Au, Au-M and M-M binding energy, were considered to influence the evolution of nanostructures.

The Monte Carlo simulation includes three types of events:

1. The addition of a single Au or M atom on the surface vacant sites;
2. The removal of a single Au or M atom from the surface of the nanostructure;
3. The replacement of a single Au (or M) atom on the surface with a single M (or Au) atom.

The steps in the Monte Carlo simulation are listed below:

1. Sample the event type with equal probability: addition, removal or replacement.
2. Determine the type of the atom related to the event according to the rates of $k_{on,Au}$ and $k_{on,M}$. They indicate the relative rates of the kinetic process involving Au and M atoms respectively.
    a. For the addition or removal event, $k_{on,Au}$ and $k_{on,M}$ are proportional to the probability of addition or removal of atom type Au and M.
    b. For the replacement event, we assumed the attachment of a specific type of atom to the surface triggered the replacement process, thus $k_{on,Au}$ and $k_{on,M}$ are proportional to selecting the type of atom that is originally in the solution phase and will be inserted into the nanostructure, e.g., a higher $k_{on,Au}$ will cause more trials to replace the surface site M with Au.

We denote the selected atom type that will be added, removed and replaced as x and the unselected atom type as y for the following descriptions.

3. If an addition event with atom type x is tried, select a vacant site on the surface with uniform distribution. The probability of accepting this event is calculated as:

$$p = \frac{1}{1 + 1/fc} \tag{70}$$

$$fc = \frac{N_{vac}}{N_{surf,x}} e^{-\beta\left(-\mu_x - (\varepsilon_{x-Au} n_{x-Au} + \varepsilon_{x-M} n_{x-M})\right)} \tag{71}$$

   where N_{vac} is the number of vacant sites and $N_{surf,x}$ is the number of the surface sites with atom type x. μ_x is the chemical potential difference in the solution and the nanostructure surface for atom type x. ε_{x-Au} and ε_{x-M} are the binding energy of the added atom with Au and M, while n_{x-Au} and n_{x-M} are the corresponding coordination numbers to Au and M respectively at the site where the atom will be added, and $\beta = \frac{1}{k_B T}$. k_B is Boltzmann constant and T is the temperature.

4. If a removal event is tried, select an occupied surface site with equal probability. The probability of accepting this event is calculated as:

$$p = \frac{1}{1 + 1/fc} \tag{72}$$

$$fc = \frac{N_{surf,x}}{N_{vac}} e^{-\beta\left(\mu_x + (\varepsilon_{x-Au} n_{x-Au} + \varepsilon_{x-M} n_{x-M})\right)} \tag{73}$$

   The corresponding parameters are described above.

5. If a replacement event is tried, select one surface site of atom type x from the nanostructure to be replaced by atom type y. The probability of accepting this event is calculated as:

$$p = \frac{1}{1 + 1/fc} \tag{74}$$

$$fc = \frac{N_{surf,x}}{N_{surf,y}} e^{-\beta\left(\mu_x - \mu_y + (\varepsilon_{x-Au} n_{x-Au} + \varepsilon_{x-M} n_{x-M}) - (\varepsilon_{y-Au} n_{y-Au} + \varepsilon_{y-M} n_{y-M})\right)} \tag{75}$$

   where $N_{surf,x}$ and $N_{surf,y}$ are the numbers of surface sites with atom type x and y respectively. The additional parameters are defined so that μ_y is the chemical potential difference in the solution and the surface of nanostructure for atom type y, ε_{y-Au} and ε_{y-M} are the binding energy of atom type y with Au and M, while n_{y-Au} and n_{y-M} are the

corresponding coordination numbers.

6. Steps 1-5 are repeated until the simulation is finished.

In the simulation, we fix $k_{on,M}$ =1, $k_B T = k_B \times 300K = 0.0259\ eV$ and $\varepsilon_{Au-Au} = 0.3275\ eV$. To study the various nanostructures from different kinetic conditions, we scanned multiple parameters including $k_{on,Au}$, μ_{Au}, μ_M, ε_{Au-M} and ε_{M-M}. Their corresponding ranges were listed below:

1. $k_{on,Au}$ in the range of $[0.1, 1.0]$ with an interval of 0.3 (four choices).
2. μ_{Au} in the range of $[-6\varepsilon_{Au-Au}, -4\varepsilon_{Au-Au}]$ with an interval of $0.2\varepsilon_{Au-Au}$ (11 choices).
3. μ_M in the range of $[-6\varepsilon_{M-M}, -4\varepsilon_{M-M}]$ with an interval of $0.2\varepsilon_{M-M}$ (11 choices).
4. ε_{Au-M} in the range of $[0.1\varepsilon_{Au-Au}, \varepsilon_{Au-Au}]$ with an interval of $0.3\varepsilon_{Au-Au}$ (four choices).
5. ε_{M-M} in the range of $[0.1\varepsilon_{Au-Au}, \varepsilon_{Au-Au}]$ with an interval of $0.3\varepsilon_{Au-Au}$ (four choices).

Thus, $4 \times 11 \times 11 \times 4 \times 4 = 7744$ simulations were performed to get the diversified nanostructures. During the simulation, we selected an elongated truncated octahedron as the initial nanostructure. In the Monte Carlo process, we labelled the first try as step 0, while the simulation finished after step 500000. The growth, etching, and equilibrium with the initial seed were observed. The principal component analysis (PCA) was performed on the data including the five parameters together with the interested property (e.g., surface Au atom number) to demonstrate the variance and was shown in the manuscript.

After the Monte Carlo simulations, it is crucial to convert the atomic transformation into dipole transformation. During this process, from the initial structure, atoms can be added, removed, or replaced. All these atoms (including the atoms from the initial structure) are *relevant* to the structural transformation. Cubic grids representing dipoles were initially created, which included all the *relevant* atoms to the transformation. Here, one dipole can contain multiple atoms. Then, *the overall dipole set* that should be considered in simulating the trajectory was generated according to the *relevant* atoms. For the dipoles from the initial cubic grids, we will judge which dipoles should be added to the *overall dipole set*. Since the existence of a dipole will only be considered when it is fully occupied by atoms, a dipole will be added to *the overall dipole set* if all the atom sites within it are *relevant* during the structural transformation. When the dipole corresponds to the medium, its polarizability was set as $\tau \approx 0$. This approach was implemented in all the Monte Carlo simulations.

Once we find potentially interesting final structures as well as their trajectories, the emerging structures along kinetic Monte Carlo simulations were transformed into their equivalent dipole representations for RD-DDA simulations. The conversion from the atomic model to the dipole model was realized by filling the space of dipoles with a length of 1.5 times the lattice constant of Au. Up to 32 atoms can occupy such dipoles. When all the dipoles were occupied, we assume the dipole

exists, otherwise it does not exist, which defined the addition/removal of dipoles from the system. The polarizability of the dipole was calculated according to the weighted average refractive index of atom Au and M within the dipole. To be specific, the weighted refractive index for the dipole was calculated according to the numbers of Au and M within the dipole first. Then the polarizability was calculated through the FCD method using this refractive index. Thus, the replacement event causes variation in the polarizability of dipoles. By varying the polarizability due to the change in the ratio of two metal atoms within one dipole, the change of UV-Vis signals was also tracked. For demonstration, metal M is assigned as Ag and its relevant properties were used in this simulation.

During the simulation, 128-digit numeric precision is used and $\tau = 10^{-6}\alpha$ is used to define the polarizability of the medium, while α is the polarizability when the dipole is purely composed of Au. The dipole length was set to $0.615\ nm$, which is approximately 1.5 times the lattice constant of the FCC Au. Only when the lattice is fully occupied, the polarizability of it will be changed from medium to α. α is calculated from the weighted refractive index according to the numbers of Au and M atoms within the dipole, thus can vary when the replacement event happens. We sampled the extinction coefficients at the wavelength from 400 nm to 800 nm with an interval of 10 nm.

3. References

(1) Draine, B. T.; Flatau, P. J. Discrete-Dipole Approximation For Scattering Calculations. *J. Opt. Soc. Am. A* **1994**, *11* (4), 1491. https://doi.org/10.1364/josaa.11.001491.

(2) Draine, B. T.; Flatau, P. J. User Guide for the Discrete Dipole Approximation Code DDSCAT 6.1. **2004**, *3* (1994).

(3) Jiang, Y.; Salley, D.; Sharma, A.; Keenan, G.; Mullin, M.; Cronin, L. An Artificial Intelligence Enabled Chemical Synthesis Robot for Exploration and Optimization of Nanomaterials. *Sci. Adv.* **2022**, *8* (40), 2626. https://doi.org/10.1126/sciadv.abo2626.

(4) Piller, N. B.; Martin, O. J. F. Increasing the Performance of the Coupled-Dipole Approximation: A Spectral Approach. *IEEE Trans. Antennas Propag.* **1998**, *46* (8), 1126–1137. https://doi.org/10.1109/8.718567.

(5) Yurkin, M. A.; Min, M.; Hoekstra, A. G. Application of the Discrete Dipole Approximation to Very Large Refractive Indices: Filtered Coupled Dipoles Revived. *Phys. Rev. E - Stat. Nonlinear, Soft Matter Phys.* **2010**, *82* (3), 1–12. https://doi.org/10.1103/PhysRevE.82.036703.

(6) P. B. Johnson and R. W. Christy. Optical Constant of the Nobel Metals. *Phys. Rev. B* **1972**, *6* (12), 4370–4379.

(7) Miller, K. S. On the Inverse of the Sum of Matrices. *Math. Mag.* **1981**, *54* (2), 67–72. https://doi.org/10.2307/2690437.

(8) Yurkin, M. A.; Hoekstra, A. G. The Discrete Dipole Approximation: An Overview and Recent Developments. *J. Quant. Spectrosc. Radiat. Transf.* **2007**, *106* (1–3), 558–589. https://doi.org/10.1016/j.jqsrt.2007.01.034.

(9) Carbó-Argibay, E.; Rodríguez-González, B.; Pacifico, J.; Pastoriza-Santos, I.; Pérez-Juste, J.; Liz-Marzán, L. M. Chemical Sharpening of Gold Nanorods: The Rod-to-Octahedron Transition. *Angew. Chemie - Int. Ed.* **2007**, *46* (47), 8983–8987. https://doi.org/10.1002/anie.200703259.

(10) Ye, X.; Jones, M. R.; Frechette, L. B.; Chen, Q.; Powers, A. S.; Ercius, P.; Dunn, G.; Rotskoff, G. M.; Nguyen, S. C.; Adiga, V. P.; Zettl, A.; Rabani, E.; Geissler, P. L.; Alivisatos, A. P. Single-Particle Mapping of Nonequilibrium Nanocrystal Transformations. *Science (80-.).* **2016**, *354* (6314), 874–877. https://doi.org/10.1126/science.aah4434.
