# Supplementary material for: An Accelerated Method for Investigating Spectral Properties of Dynamically Evolving Nanostructures
Source: J Phys Chem Lett. 2023 Apr 20;14(16):3929–38. doi: 10.1021/acs.jpclett.3c00395 (PMC10150391; doi:10.1021/acs.jpclett.3c00395)
Supplement: Supplementary file 3 — jz3c00395_si_003.pdf [file jz3c00395_si_003.pdf]

jz-2023-00395j.R1

Name: Peer Review Information for "An Accelerated Method for Investigating Spectral Properties of Dynamically Evolving Nanostructures"

First Round of Reviewer Comments

Reviewer: 1

Comments to the Author

The manuscript reports on a modified DDA algorithm to calculate the optical properties of nanocrystals under structural modifications in a recursive way, namely without the need of calculating independently each different structural step each time. There is no benchmark with experiments, although the aim of the manuscript is focused on the algorithm only. Overall quality of the manuscript and supporting material is very good. The best aspect is the combination with kinetic montecarlo, which makes the study useful. I suggest publication after minimal revisions in a more specialized journal of nanomaterials or computational chemistry.

caption of figure 1 does not explain well the chemical process described (which is indeed mentioned in caption of figure 2).

Corrosion processes are not mentioned in the introduction and may be relevant for the code as well (see for instance Nano Lett. 2014, 14, 2569– 2577 and ACS Nano 2020, 14, 10, 12840–12853)

For colloidal dispersions, orientational average is required. This aspect could be commented and explained more in the text.

The mathematical model can stay just in the supp info.

Reviewer: 2

Comments to the Author

The authors present a clever new optical simulation tool, the "rank-one decomposition accelerated discrete-dipole approximation (RD-DDA)", which allows for systematic evolution of a large, but relatively simple, nanostructure to a more complex one---efficiently computing the response for each intermediate state along the way. The authors characterize and benchmark the method, and demonstrate how it can be used (e.g., combing w/ kinetic MC to model the extinction spectra for Au nanospheres etched from octahedra or structural transformations in bimetallic nanostructures).

It's an interesting advance and a clearly written, intellectually stimulating paper that could be of broad interest to the JPC Lett readership.

The following items should be addressed in the manuscript before being considered for publication.

(1) The method provides a way to efficiently generate optical extinction spectra for every intermediate in between an initial and final configuration. However, for many (most?) applications, much less information is needed. This point should be acknowledged. I suspect readers will be interested in comparing the cumulative simulation time needed for RD-DDA versus direct solution when

a. Only the final state is of interest.

b. Only M (e.g., equally spaced) intermediates are of interest. Here M may be as few as 5 or 10

The authors could present such information clearly in a table (picking a few representative M) for the cases studied.

Is there a way to estimate (in advance) how this comparison scales as a function of M and number of dipoles to assess whether it make sense adopt RD-DDA for a given application? I suspect a clear discussion on this point would also increase the impact of the work.

(2) The authors should discuss in the main text the relevance/tradeoffs of using 128-digit numeric precision. It wasn't clear to me that 128-digit precision is needed for the direct solutions. If not, are the comparisons in Fig. 1c the practically relevant ones? Or should the comparison be against a more standardly used precision choices for direct solution by DDA?

Author's Response to Peer Review Comments:

The comments from reviewers and editors are in *italics* and our reply is in normal format.

We highly appreciate the comments from both reviewers and the editorial team. Reviewer 1's comments are focused on modifying the content of the manuscript with additional details to make it more accessible to readers. Reviewer 2's comments focused on the practical usage of RD-DDA, such as describing a selection criteria between the RD-DDA method and direct implementation of DDA. We have answered all the questions from the reviewers and made the required modifications in the manuscript and SI. We believe we have addressed the reviewers' and editor's comments which could substantially increase the impact of this work.

Reviewer(s)' Comments to Author:

Reviewer: 1

Recommendation: This paper is publishable subject to minor revisions noted. Further review is not needed.

Comments:
The manuscript reports on a modified DDA algorithm to calculate the optical properties of nanocrystals under structural modifications in a recursive way, namely without the need of calculating independently each different structural step each time. There is no benchmark with experiments, although the aim of the manuscript is focused on the algorithm only. Overall quality of the manuscript and supporting material is very good. The best aspect is the combination with kinetic montecarlo, which makes the study useful. I suggest publication after minimal revisions in a more specialized journal of nanomaterials or computational chemistry.

We acknowledge Reviewer 1's inspiring comments and believe this work can be an important theoretical tool to study the optical properties of nanomaterials, and will also be of wide interest to researchers in physical chemistry, computational chemistry and material science and is suitable for publication in *Journal of Physical Chemistry Letters*.

caption of figure 1 does not explain well the chemical process described (which is indeed mentioned in caption of figure 2).

We have added the explanation to the caption of **Figure 1** to clarify it is a random coating process which describes the deposition of a thin layer of Ag on the surface of the Au octahedron (see the added sentence in the caption of **Figure 1**, "The deposition of a single Ag layer on the surface of Au octahedra with a random growth process is shown here, forming a Au@Ag octahedron with an edge length of 20 nm.").

Corrosion processes are not mentioned in the introduction and may be relevant for the code as well (see for instance Nano Lett. 2014, 14, 2569– 2577 and ACS Nano 2020, 14, 10, 12840–12853)

We did mention both the growth and etching (corrosion) process in the introduction but did not put any citations there, which can make the readers miss the point. To address the reviewer's concern, we have added relevant citations to the introduction (see "The growth and etching mechanisms of nanoparticles[23,26–28] are complex and usually require *in situ* imaging techniques such as Transmission Electron Microscopy (TEM)[23] and Atomic Force Microscopy (AFM)[28]" in the manuscript).

For colloidal dispersions, orientational average is required. This aspect could be commented and explained more in the text.

Yes, the orientation average is crucial for the simulation of colloidal dispersions indeed. We did not consider any preferred orientations during the simulation. Instead, Tait–Bryan angles with 10 sampling points in each angle dimension to represent the uniform distribution of different orientations of the nanostructure were used. Thus, the same nanostructure will interact with the incident beam in (10×10×10) orientations, with all the spectra simulated. Later, these spectra calculated from 1000 orientations were averaged to get the final extinction spectrum. Here, we change the orientations of the incident beam instead of the nanostructures during the calculation (by doing so, we do not need to write a different $\boldsymbol{A}$ matrix and its inverse for each orientation). If not mentioned otherwise, the spectra that we showed in the paper/SI were all averaged from such 1000 spectra to consider the orientational average. We have added this content to the manuscript (see the end of page 6, "If not mentioned otherwise, …, to calculate an orientation-averaged spectrum through this work.").

The mathematical model can stay just in the supp info.

We have put the majority of the mathematical details in the supplementary information (SI) including complete derivations of the RD-DDA method, but we do believe a few equations that give the readers

a clear picture and show the recursive relation from the original solution to the new solution is essential. Thus, we would like to keep the minimalistic mathematical model in the manuscript for the explanation and refer to SI for details.

Additional Questions:
Urgency: Moderate

Significance: High

Novelty: Moderate

Scholarly Presentation: Top 10%

Is the paper likely to interest a substantial number of physical chemists, not just specialists working in the authors' area of research?: No

Reviewer: 2

Recommendation: This paper may be publishable, but major revision is needed; I would like to be invited to review any future revision.

Comments:
The authors present a clever new optical simulation tool, the "rank-one decomposition accelerated discrete-dipole approximation (RD-DDA)", which allows for systematic evolution of a large, but relatively simple, nanostructure to a more complex one---efficiently computing the response for each intermediate state along the way. The authors characterize and benchmark the method, and demonstrate how it can be used (e.g., combing w/ kinetic MC to model the extinction spectra for Au nanospheres etched from octahedra or structural transformations in bimetallic nanostructures).

It's an interesting advance and a clearly written, intellectually stimulating paper that could be of broad interest to the JPC Lett readership.

We highly appreciate the reviewer's detailed revision of the contents and the reviewer's comments on the work.

The following items should be addressed in the manuscript before being considered for publication.

(1) The method provides a way to efficiently generate optical extinction spectra for every intermediate in between an initial and final configuration. However, for many (most?) applications, much less information is needed. This point should be acknowledged. I suspect readers will be interested in comparing the cumulative simulation time needed for RD-DDA versus direct solution when

a. Only the final state is of interest.

b. Only M (e.g., equally spaced) intermediates are of interest. Here M may be as few as 5 or 10

The authors could present such information clearly in a table (picking a few representative M) for the cases studied.

We acknowledge the reviewer's question about when this method should be implemented from the practical aspect and take advantage of the acceleration. When only the final state is of interest (i.e, only need to simulate for one nanostructure), a direct DDA method should be used. The direct solution will be suitable when we need to perform the scattering simulation for only one/or a few nanostructures. For both cases (a) and (b) here, the direct solution is the more suitable option.

However, RD-DDA is a powerful tool when the simulation of more intermediates is needed such as to investigate the kinetics of morphological transformations. As an example, we selected the case from **Figure 1** (coating of Ag on Au octahedra) to show the time-cost ratio of implementing RD-DDA and direct solution when a different amount of intermediates were required (see the figure below, which was also added to SI as **Figure S9**). When more than 33 (at the most, when RD-DDA is in 128-digit precision and the direct solution is in 64-digit precision) intermediates were used, implementing RD-DDA shows clear acceleration in the algorithmic performance. As suggested by the reviewer, the same figure is used to show the time cost for every possible intermediate number (instead of a table). An additional figure for the case of nanosphere growth is also added to the SI as **Figure S10**.

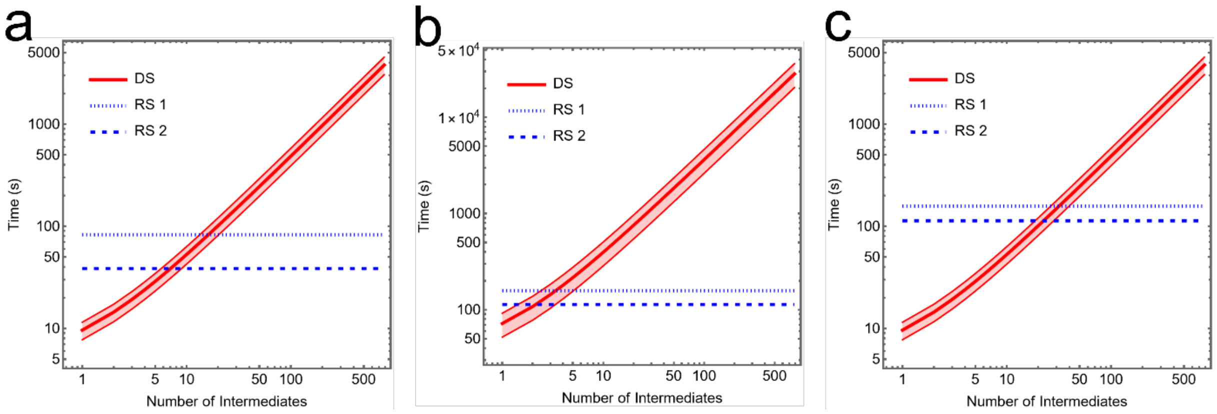

The comparison of the time cost when the different numbers of intermediates are needed for the simulation of Ag coating on Au octahedra. The computational times from different methods are shown. RD-DDA and the direct solution method were implemented in the same 64-digit precision in (a) and 128-digit precision in (b) respectively. In (c), RD-DDA was implemented with 128-digit precision and the direct solution method was implemented with a 64-digit precision.

We agree that some cases may not need the information when the change of “a single dipole” occurs. However, simulating a kinetic process with a small number of intermediates (5–10) is an extreme case – where few spectral information of a few intermediates is too small to give good enough insights into the whole process. For example, to validate a kinetic process describing nanostructural transformations using in-situ spectroscopy, a detailed theoretical simulation with a finer structural resolution is required. So, the more intermediates were used, the more precisely the change in spectral behaviour can be captured – which is crucial for mechanistic insights for nanostructural transformations. RD-DDA makes it possible to track detailed changes and is more efficient as long as the intermediate number is not extremely small (see **Figure S9-10**). Another promising application of RD-DDA is to investigate the influence of the local electric field on the kinetic process. Due to the strong coupling between the local electric field distribution and dynamics at the surface, the minimal structural changes need to be tracked and the kinetic parameters to be updated accordingly. Thus, we believe RD-DDA aligns well with many applications where the optical properties of evolving nanostructures need to be calculated and will be widely applied in the future.

Is there a way to estimate (in advance) how this comparison scales as a function of M and number of dipoles to assess whether it make sense adopt RD-DDA for a given application? I suspect a clear discussion on this point would also increase the impact of the work.

This is a very good point and we highly appreciate the reviewer's critical insights. Here we describe, a general strategy to estimate the time cost for both methods. RD-DDA relies mainly on matrix manipulation, the time cost for every update in RD-DDA scales with the matrix size (or equivalently, the dipole number). We can estimate the time cost for both methods (i.e, RD-DDA and direct solution)

for a given trajectory/intermediates, but it requires solving several linear systems unavoidably (up to five, see below).

First, we can estimate the time cost for the direct solution straightforwardly. We need to find the largest (assume it consists of N_{upper} dipoles) and smallest structures (assume it consists of N_{lower} dipoles) during the process describing the structural transformation. Then we can implement DDA on them directly and record the time cost, which corresponds to the estimated upper/lower boundary of the time cost (t_{upper}, t_{lower}) for one intermediate in the direct solution. If we have M nanostructures to calculate in total, the time cost will be estimated in the range of $[M \times t_{lower}, M \times t_{upper}]$ for the direct solution.

In the meantime, in the benchmark cases of RD-DDA simulation with varied matrix sizes (SI Section 1.6) for Ag coating and nanosphere growth, we observed the time cost for one update is linearly correlated to the number of dipoles (equivalently, the matrix sizes), as shown in the figure below.

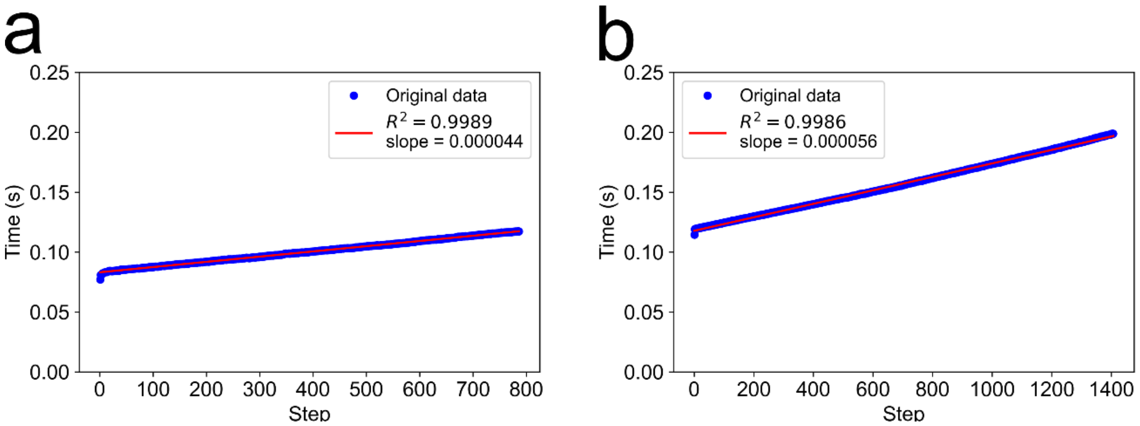

The raw data of the time cost to obtain a new solution in RD-DDA and its corresponding linear fitting. (a) The time cost and its linear fitting to perform one update (i.e., to solve one new system after the change of one dipole) using RD-DDA during the coating of Ag on the surface of Au octahedra with RS 1. The same raw data is used in **Figure 1b** in the manuscript. (b) The time cost and its linear fitting to perform one update in the growth of Au nanospheres with RS 1. The same raw data is used in **Figure S8**.

Thus, by assuming the time cost for one update (after a dipole change) towards a different nanostructure is linearly correlated to the existing dipole number (thus the matrix size), one possible strategy to estimate the time cost for RD-DDA is proposed as follows:

1. If we know the trajectory, we can estimate the lowest and highest time cost for one update by performing the update on the smallest system (with N_1 dipoles) and the largest system (with N_2 dipoles) where the update can happen, which will give the lower/upper time cost estimation of one update as t_1 and t_2, respectively.
2. Then we can create a linear curve using (N_1, t_1) and (N_2, t_2). The linear curve will be used to

estimate the time cost (t_i) when we perform one update on the intermediate with a dipole number of N_i.

3. When we perform RD-DDA, we will solve the initial structure with a time cost t_{init}. Then the overall time cost for RD-DDA would be $t_{RD-DDA} = t_{init} + \sum_i t_i$, where t_i is the time cost for acquiring the new solution after the change of one dipole in the trajectory, and can be obtained efficiently from the linear curve from Step (2). With this method, t_{init} is precise and $\sum_i t_i$ is estimated.

With the procedure described above, the time cost of the estimation process itself will be mainly from solving the large linear systems in advance. Up to five linear systems correspond to (1) the smallest nanostructure in the trajectory, (2) the largest nanostructure in the trajectory, (3) the initial nanostructure for RD-DDA, (4) the smallest nanostructure where the RD-DDA update is performed, (5) the largest nanostructure where the RD-DDA update is performed.

We validate if the linear interpolation strategy is good to estimate the RD-DDA time cost in the two benchmark cases described above. Specifically, we can estimate the time cost ($\sum_i t_i$) for all the possible small trajectories from the whole growth trajectory for validation. These small trajectories include at least two updates to create the linear curve.

On the one hand, we have recorded the actual update time cost for these small trajectories (i.e., the time cost for RS 1 in **Figure 1b** and **Figure S8**). On the other hand, we have also recorded the time cost for one update for both the smallest and largest structures (two points), which can be used to create a linear curve to estimate the $\sum_i t_i$ term as described above. By comparing the actual and estimated time cost for all the small trajectories, the relative error due to the estimation can be calculated (see figure below, which is added to SI as **Figure S12**). The maximal relative error from the estimation is 3.77% for the case of the growth of Ag on the surface of Au octahedra and 2.00% for the case of the growth of nanospheres, respectively.

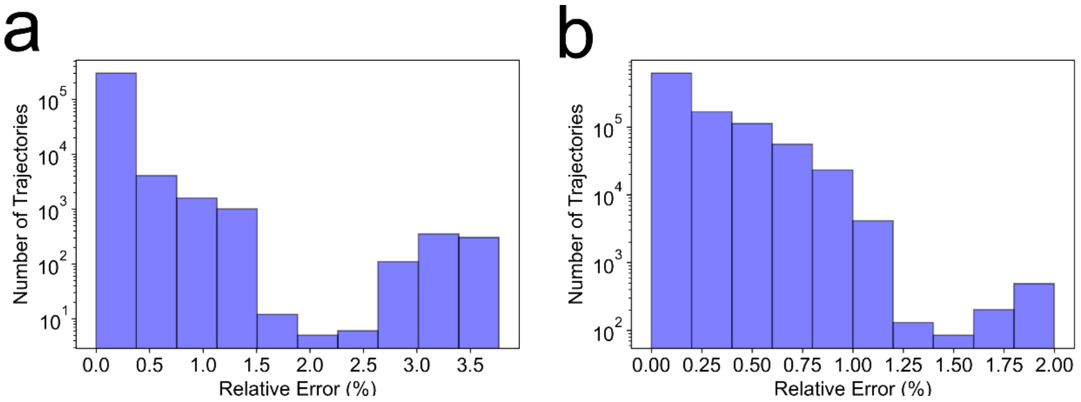

Histogram of the relative error for the time estimation ($\sum_i t_i$) for all possible small trajectories. (a) The relative error distribution of the estimated time for the small trajectories from the growth of a Ag layer on Au octahedra. The total number of small trajectories is 308505, with a maximal relative error of 3.77%. (b) The relative error distribution of the estimated time for the small trajectories from

the growth of Au nanospheres. The total number of small trajectories is 987715, with a maximal relative error of 2.00%. It should be noted that if the number of updates is two for the small trajectory, $\sum_i t_i$ will be the precise time cost and will give a relative error of 0. The number of such “two-update” trajectories is 785 and 1405 for (a) and (b), respectively.

Finally, for comparison, if t_{RD-DDA} is smaller than $M \times t_{lower}$ (from the estimation of the direct solution method), RD-DDA will be more efficient. When t_{RD-DDA} is larger than $M \times t_{upper}$, RD-DDA is not suitable for this application. However, when t_{RD-DDA} is in the range of $\left[M \times t_{lower}, M \times t_{upper}\right]$, it will depend on the specific intermediates and the algorithm used to solve the linear system. One simple solution is to use the average of $M \times t_{lower}$ and $M \times t_{upper}$ to see if RD-DDA is more efficient. In the meantime, a more precise comparison will need to estimate the time cost to solve the intermediates. For instance, if we assume the time cost to solve a linear system is proportional to n^3 (where n is the matrix size) for simplicity, we need to get the number of dipoles for the intermediates and estimate the time cost for every intermediate by linear interpolation using $\left({N_{lower}}^3, t_{lower}\right)$ and $\left({N_{upper}}^3, t_{upper}\right)$. Then we can estimate the time cost for solving these N intermediates more accurately. Eventually, comparing it with t_{RD-DDA} can tell if we should use RD-DDA or the direct solution method.

The relevant contents from the discussion above are added to SI Section 1.7 and we highly appreciate the comment from the reviewer.

(2) The authors should discuss in the main text the relevance/tradeoffs of using 128-digit numeric precision. It wasn’t clear to me that 128-digit precision is needed for the direct solutions. If not, are the comparisons in Fig. 1c the practically relevant ones? Or should the comparison be against a more standardly used precision choices for direct solution by DDA?

The use of 128-digit precision is not needed for direct solutions. While 64-digit is generally good enough, 128-digit precision is needed only when we utilized the approximation in RD-DDA to change the polarizability of a dipole from medium (τ) to the actual material (α_j) for numerical stability, which is discussed in SI Section 1. To address the reviewer’s advice related to the relevance/tradeoff of 128-digit precision, we have added additional description in the manuscript for the usage of 128-digit precision and guided the readers to the relevant content in the SI (see the content from “while 128-digit precision is needed, …, (see SI Section 1.5-1.6).” on page 6 in the manuscript).

The comparison in **Figure 1c** is used to demonstrate the acceleration purely from the mathematical formulation in RD-DDA, which is a comparison based on the method itself. We set both RD-DDA and direct solutions in the same 128-digit precision for a fair comparison. However, as said by the reviewer,

a practically relevant comparison should be made when we put the direct solution method in its standard conditions, which is 64-digit precision. To address the reviewer's concern, we have made this comparison and added the relevant figures/descriptions in **Figure S7**, and have described and referred to it from the manuscript (see "While a high numeric precision (128-digit) is not necessary for the direct implementation of DDA, 24 and 33 times accelerations were observed when the direct DDA was implemented under 64-digit precision and RD-DDA under 128-digit precision (Figure S7)." on page 3 in the manuscript).

Additional Questions:
Urgency: High

Significance: High

Novelty: High

Scholarly Presentation: High

Is the paper likely to interest a substantial number of physical chemists, not just specialists working in the authors' area of research?: Yes

----
Formatting Notes from the Editorial Office:

1. Please resize the TOC graphic per journal guidelines (2 in x 2 in) and remove the caption below it.
Yes, the TOC is resized and the new TOC is added to the manuscript.
2. Please remove the section heading(s) throughout the body of the manuscript (you can leave Methods and Abstract headings).
Yes, the section headings in the manuscript are removed.
*3. In both the main file and the supporting information, fix the style of all references to use JPCL formatting (check all references carefully). ***JPC Letters reference formatting requires that journal references should contain: () around numbers, author names, article title (titles entirely in title case or entirely in lower case), abbreviated journal title (italicized), year (bolded), volume (italicized), and pages (first-last). Book references should contain author names, book title (in the same pattern), publisher, city, and year. Websites must include date of access.*
We have checked the reference format and made sure it is the JPCL format.
4. Please move the Author Information section above the references.

The Author Information has been moved above now.

5. Please number SI pages in the following format: "S1, S2…"

The page of the SI is labelled as requested now.

6. Please label all graphics in the following format: "Figure S1, S2…", "Scheme S1, S2…." or "Table S1, S2…", etc. (not 1.1, 1.2, etc.) and update the in-text citations as needed.

The figures/tables/schemes are labelled as requested now.

7. Please add the captions for the videos to the SI file.

The video captions are added below the Table of Content in the SI file now.

8. Please include annotated version(s) of your revised publication file(s) with colored text or highlights indicating the revisions that you have made, and upload them as "Supporting Information for Review Only." Please also upload "clean" copies for publication. (No highlighting, annotations, or colored text.)

Both files (with and without highlights) are prepared now.

----

jz-2023-00395j.R2

Name: Peer Review Information for "An Accelerated Method for Investigating Spectral Properties of Dynamically Evolving Nanostructures"

Second Round of Reviewer Comments

Reviewer: 2

Comments to the Author

The authors did an outstanding job of addressing the points raised in review.

Author's Response to Peer Review Comments:

Formatting Notes from the Editorial Office:

1. Please remove the Introduction, Results and Discussion, and Conclusions section heading(s) throughout the body of the manuscript (you can leave Methods and Abstract headings).

Yes, the headings are deleted with only one heading of “Main Text” at the start.

2. Please move the Acknowledgments section above the references.

Yes, the Acknowledgement section has been moved.

3. Please explain parts a and b in the Figure S14 caption.

Yes, the additional description of “The peak positions and prominences in the process are shown in (a) and (b) respectively. ” has been added to the caption.

4. Please explain parts a-c in the Figure S15 caption.

Yes, the additional explanation of “The spectra from the trajectory 1, 2 and 3 are shown in (a), (b) and (c) respectively.” has been added to the caption.

5. Please submit your publication files without any markups. Any copies that contain highlights, colored text, or tracked changes should be submitted as "Supporting Information for Review Only."

The revised version of the SI (without markups) has been prepared.
